# Revealing the selection history of adaptive loci using genome-wide scans for selection: an example from domestic sheep

**DOI:** 10.1186/s12864-018-4447-x

**Published:** 2018-01-23

**Authors:** Christina Marie Rochus, Flavie Tortereau, Florence Plisson-Petit, Gwendal Restoux, Carole Moreno-Romieux, Gwenola Tosser-Klopp, Bertrand Servin

**Affiliations:** 1 GenPhySE, Université de Toulouse, INRA, INPT, ENVT, 313 26 Castanet Tolosan, France; 2 UFR Génétique, Élevage et Reproduction, AgroParisTech, Université Paris-Saclay, 752 31 Paris, France; 3 Department of Animal Breeding and Genetics, Faculty of Veterinary Medicine and Animal Science, Swedish University of Agricultural Sciences, P.O. Box 7023, 750 07 Uppsala, Sweden; 4 Génétique Animale et Biologie Intégrative, INRA, AgroParisTech, Université Paris-Saclay, 752 31 Paris, France

**Keywords:** Selective sweeps, Introgression, Allelic heterogeneity, Adaptation

## Abstract

**Background:**

One of the approaches to detect genetics variants affecting fitness traits is to identify their surrounding genomic signatures of past selection. With established methods for detecting selection signatures and the current and future availability of large datasets, such studies should have the power to not only detect these signatures but also to infer their selective histories. Domesticated animals offer a powerful model for these approaches as they adapted rapidly to environmental and human-mediated constraints in a relatively short time. We investigated this question by studying a large dataset of 542 individuals from 27 domestic sheep populations raised in France, genotyped for more than 500,000 SNPs.

**Results:**

Population structure analysis revealed that this set of populations harbour a large part of European sheep diversity in a small geographical area, offering a powerful model for the study of adaptation. Identification of extreme SNP and haplotype frequency differences between populations listed 126 genomic regions likely affected by selection. These signatures revealed selection at loci commonly identified as selection targets in many species (“selection hotspots”) including *ABCG2*, *LCORL*/*NCAPG*, *MSTN*, and coat colour genes such as *ASIP*, *MC1R*, *MITF*, and *TYRP1*. For one of these regions (*ABCG2*, *LCORL*/*NCAPG*), we could propose a historical scenario leading to the introgression of an adaptive allele into a new genetic background. Among selection signatures, we found clear evidence for parallel selection events in different genetic backgrounds, most likely for different mutations. We confirmed this allelic heterogeneity in one case by resequencing the *MC1R* gene in three black-faced breeds.

**Conclusions:**

Our study illustrates how dense genetic data in multiple populations allows the deciphering of evolutionary history of populations and of their adaptive mutations.

**Electronic supplementary material:**

The online version of this article (10.1186/s12864-018-4447-x) contains supplementary material, which is available to authorized users.

## Background

Identifying genes and polymorphisms responsible for the variation of complex traits is a fundamental question in genetics. The most common approach to tackle this problem consists of looking for polymorphisms that affect a recorded trait (i.e. QTLs) using linkage analysis or genome-wide association studies. For traits that affect the fitness of individuals (their ability to produce viable offspring), the diversity around favourable alleles at QTLs will change over time, due to selection. Hence, identifying genome regions that have experienced such effects (selection signatures) can be used to detect past but also currently segregating QTLs. In domesticated livestock species, due to human selection, part of the traits that govern the fitness of individuals should also be associated to their agricultural performances. As such, studying selection signatures in livestock offers a valuable approach to identify genes and polymorphisms that underlie variation in these traits. The power to detect such signatures depends on two things: sample size (both of the number of individuals and populations included in the studies) and marker density [1]. As datasets become larger, it should be possible to generate evolutionary hypotheses on the history of selection signatures. In this work, we exploited a large sheep dataset in a relatively small geographic area (France) to this goal.

Sheep, one of the first domesticated species, originated from a population of *Ovis orientalis* in the fertile crescent in contemporary Iran [2, 3]. From its domestication centre, sheep eventually spread world-wide, adapting to a large range of environmental conditions. In agriculture, sheep are raised for meat, wool and milk products, with commercial breeds usually specialised for one type of production. This history has shaped sheep genetic diversity and makes it an interesting model species to study the evolution of populations under adaptive constraints [4, 5].

In a recent study, the International Sheep Genomics Consortium (ISGC) [6] compiled a dataset of DNA samples from 74 populations of sheep from around the world genotyped for approximately 50,000 SNP markers: the Sheep HapMap dataset (http://www.sheephapmap.org/). Analyses of this dataset showed both clear geographical structure of breeds and strong admixture among breeds. In Western Europe, geographical structure was clear with several population groups identified that corresponded to their area of origin: Italian, Iberian, Swiss, British and Northern European groups [6]. Only one French sheep population, the Lacaune breed, was included in this study. It clustered in the Iberian group, consistent with its origin from the south-western region of France. Besides the Lacaune breed, France harbours many other different sheep breeds across the country and because of its geographical position, France borders many of the geographic areas associated with western European sheep population groups of the Sheep HapMap dataset. The study of French sheep diversity should therefore provide valuable insights on the establishment of genetic structure in European sheep, and in particular inform us on the history of sheep husbandry in Europe. French sheep diversity was recently studied with the genotyping of 21 microsatellites in sheep from 49 breeds [7]. This study confirmed that French sheep breeds show a structure consistent with influences from both northern European, specifically British breeds, and southern European breeds. Hence, French sheep populations are likely to harbour a large part of European sheep diversity in a relatively small geographical region, offering a powerful model for the study of adaptation.

Several studies have identified signatures of selection in sheep: signatures associated with pigmentation [8], fat deposition [9], milk yield [10], adaptations to climate [5, 11] and resistance to parasites [12]. There have also been studies looking at a number of breeds: a study which included only American breeds of sheep [13] and two studies including sheep populations from around the world [6, 14]. These world-wide studies, which used data from the ISGC Sheep HapMap data set, detected signatures containing genes or QTLs associated with pigmentation, morphology, muscling, milk production and wool quality. Studying data sets with many different breeds also allows the study of diversity in selection, the different mutations and genes selected on to reach the same phenotype (ie. colour, muscling etc.). All of these studies however, relied on medium density genotyping using about 50,000 SNP markers. This limits the precision of localization of candidate genes underlying selection signatures as well as the identification of potential haplotypes carrying causal mutations.

The present study aims to enhance our understanding of the mechanisms of adaptation in livestock by providing and analysing a large data set of high density genotyping (around 600,000 SNP markers) in 542 samples from 27 French sheep populations. Our analyses of the genetic structure in this dataset identify clearly two main origins of breeds and show how these breeds complement the Sheep HapMap dataset. Then, in what is the first selection scan based on high-density genotyping in sheep, our results show clear evidence for adaptive introgression between groups along with allelic heterogeneity at some adaptive loci. Finally, we confirm this heterogeneity by re-sequencing a known coat colour gene (*MC1R*) in three populations presenting evidence for selection at this locus, and pinpoint a set of new potential causal mutations for coat colour phenotypes in sheep.

## Methods

### French sheep data

A total of 691 French sheep were genotyped for 653,305 autosomal SNPs (Illumina Ovine Infinium® HD SNP BeadChip) with 27 sheep breeds being represented in this study. The populations chosen represent the majority of commercial breeds present in France but also included some breeds maintained for conservation purposes. Breed names and their abbreviations are listed in Table 1. In each breed, genotyped animals were chosen so as to be as unrelated as possible based on pedigree records. DNA samples from this study came from various French flocks. Blood sampling was not performed specifically for this study. Blood samples were taken from commercial farms. Animals did not belong to any experimental design but were sampled by veterinarians and/or under veterinarian supervision for routine veterinary care. A test for Hardy Weinberg Equilibrium (HWE) was calculated for each SNP within each breed using PLINK [15, 16] and SNPs not in HWE (false discovery rate (FDR) 5% calculated with R package qvalue [17]) in one or more breeds were removed. For each breed, a genomic relationship matrix was computed [18]. The resulting distribution of kinship coefficients was modelled as a mixture of normal distribution, with the major component of the mixture representing pairs of unrelated individuals. Pairs of related individuals were identified as those that did not belong to this component (FDR < 5%). Finally, one individual for each pair was removed until no related individuals remained. All further analyses were performed on this set of unrelated individuals. SNPs with a minor allele frequency (MAF) = 0, SNPs with a missing call rate > 0.01 and sheep with individual missing call rate > 0.05 were removed. After quality control, 527,823 SNP markers remained and were included in analyses.

**Table 1:** Sheep population groups and breeds

Origin	Breed	Abbreviation	N
Mouflon	European Mouflon	EUR	2
	Asian Mouflon	IRN	19
Northern Europe	Berrichon du Cher	BER	19
	Charollais	CHA	24
	Charmoise	CHR	24
	Île de France	IDF	23
	Ouessant (Ushant)	OUE	18
	Romane	RMN	19
	Romanov	ROM	14
	Roussin de la Hague	ROU	23
	Rouge de l’Ouest	RWE	19
	Suffolk	SUF	21
	Texel	TEX	24
	Vendéen	VEN	22
Southern Europe	Blanche du Massif Central	BMC	22
	Causse du Lot	CDL	21
	Corse	COR	16
	Dairy Lacaune	LAC	37
	Meat Lacaune	LAM	36
	Limousine	LIM	19
	Mérinos d’Arles	MER	18
	Mérinos de Rambouillet	RAM	31
	Mourerous	MOU	20
	Manech tête rousse	MTR	25
	Noire du Velay	NVE	22
	Préalpes du Sud	PAS	20
	Rava	RAV	20
	Tarasconnaise	TAR	16

### Analysis of population structure

Three methods were used to study population structure from SNP genotypes: a principal components analysis to visualize patterns in relationships between individuals using PLINK based on the genotype matrix [15, 16]; a model based approach to estimate individual ancestry coefficients, using the software sNMF [19]; a model based approach to infer populations splits and mixtures using the software treemix [20]. We included two outgroup populations in our analyses: Asian Mouflon (*Ovis orientalis orientalis*) and European Mouflon (*Ovis aries*). Summary statistics including allele frequencies from 19 Asian Mouflon were generated and provided by the NextGen consortium (http://nextgen.epfl.ch/), as per the NextGen consortium data sharing agreement. The allele frequencies were used to root the population tree in the maximum likelihood tree analyses. Asian Mouflon were sampled in the geographic area corresponding to the cradle of domestication (i.e. North-western part of Iran [21, 22]) and tissues were collected either from captive or recently hunted animals or from frozen corpses archived by the Iranian local Department of Environment.

We also included two genotyped samples of European Mouflon in the principal component, individual ancestry coefficient and maximum likelihood tree analyses. These two European Mouflon were sampled from Sigean Park (France) from descendants of mouflon originally captured in Bavella (Corsica, France) and reintroduced in the Cadarache area since 1949. For sNMF, the optimal number of ancestral populations (K) was the one that had the lowest cross entropy criterion (Additional file 1) [19]. For treemix, only the 498,651 SNPs genotyped in both Asian Mouflon and French sheep were included. The number of migration events included in the population tree was chosen based on the comparison of the fraction of the variance in relatedness between populations that is accounted for by the model, as explained in the software documentation (Additional file 2).

To study French sheep population structure within other European breeds, a subset of breeds from the Sheep HapMap data set were included in population structure analyses; and both model based approaches, estimation of individual ancestry coefficients and inferred population splits and mixtures. The Sheep HapMap individuals included in this study were the same European sheep used by Fariello et al. [14] including breeds from; Central Europe; Southwestern Europe; North Europe; and Italy. Breeds were removed if they had a sample size smaller than 20, were a result of recent admixture or had recently experienced a severe bottleneck [14]. Shared markers between the French sheep breeds and the Sheep HapMap subset of breeds, 39,976 SNPs, were used to estimate individual ancestry coefficients using sNMF [19]. The same Iranian Asian Mouflon sheep were included in the analysis to root the population tree when estimating population splits and mixtures using treemix [20] where allele frequencies from 30 Iranian Asian Mouflon sheep from the NextGen project were included to root the population tree. For the combination of our dataset with the Sheep HapMap dataset, a total of 38,596 SNPs common to the French sheep, Sheep HapMap subset and Asian Mouflon sheep were used in the analyses.

### FLK and hapFLK genome scans

FLK [23] and hapFLK [24] tests were used to detect signatures of selection. Both tests are aimed at identifying regions of outlying differentiation between populations while accounting for their hierarchical structure. The evolutionary model underlying FLK and hapFLK assumes that SNPs were polymorphic in an ancestral population. In order to consider only SNPs matching this hypothesis, SNPs with estimated ancestral minor allele frequency < 5% were removed, leaving 465 2351 SNPs for use in the analyses. For the FLK analysis, significant SNPs within 1 Mbp from each other where grouped into a common selection signature (FDR 1%). To verify that grouping SNPs within 1 Mbp is appropriate we estimated LD decay in a subset of all populations in our dataset averaged over chromosomes (Additional file 3).

Candidate genes were identified in regions containing 10 or more significant SNPs. For hapFLK, the number of haplotype clusters has to be specified in order to fit the Scheet and Stephens [25] model. This number was set to 30, from results obtained using the cross validation procedure included in the fastPHASE software [25]. *P*-values for hapFLK were obtained by fitting a chi-squared distribution to the empirical distribution as explained by Boitard et al. [1] using the “Scaling_chi2_hapflk.py” script available on the hapFLK software web page (https://forge-dga.jouy.inra.fr/documents/588). For the hapFLK analyses, regions were constructed from significant SNPs (FDR 5%) and grouped together if they were 1 Mbp or less apart. The FLK analysis was run with all breeds and northern and southern breeds separately and these results were compared to determine the regions missed when northern and southern breeds were run separately. The hapFLK statistics were computed for northern and southern groups separately. Candidate genes were searched for in regions with five or more significant SNPs. Regions from both FLK and hapFLK results had all protein coding genes present extracted. The SNP with the lowest *p*-value in each region is referred to as the best SNP in Additional file 4. The distance from each gene to the best SNP was determined by the distance of the midpoint of the gene to the best SNP and then genes were ranked with the closest gene labelled as the best gene. Of all results, nine regions were selected for further analysis based on the candidate genes located within the regions. Local trees were constructed by re-computing Reynold’s distances between populations in a region and re-estimating branch length of the whole genome tree from local Reynold’s distances as in [24]. Local trees from single SNPs and allele frequencies in FLK results were evaluated to determine breeds selected on and for hapFLK results local trees from single SNPs and haplotypes, allele frequencies and haplotype cluster frequencies.

We re-analysed SNPs on chromosome 2 using FLK and hapFLK but without the Texel sheep population. We did this because the very large region under selection in the Texel sheep might mask selection signatures in other breeds and we were especially interested to see signals in other breeds at the *MSTN* gene.

We define allelic heterogeneity as the presence of multiple selected alleles, or haplotypes, at the same genomic location. In our analyses, we identified allelic heterogeneity in selection signatures when we had evidence for (i) more than one breed affected by selection in the same region and (ii) different haplotypes having arisen to high frequency in the selected breeds.

### Quantifying allelic heterogeneity

In order to test for multiple alleles associated to a selection signature (allelic heterogeneity) we used CAVIAR [26], a software program designed to identify independent causal SNPs in signals of association identified by genome-wide association studies. We repurposed this software to identify independent associated SNPs in detected signatures of selection. For each selection signature we detected in the FLK analysis where we included all breeds, SNPs with a FLK statistic with *p* < 0.001 were used in the CAVIAR analysis. If a region had less than 10 SNPs with *p* < 0.001 then the 10 SNPs with the smallest *p*-values were used. If a region had greater than 40 SNPs with *p* < 0.001 then the 40 SNPs with the smallest *p*-values were used. To look for multiple causal variants, CAVIAR exploits a correlation matrix between tests which, for GWAS, is a linkage disequilibrium (LD) matrix. In the case of FLK, the correlation between tests is not due to LD only, but also to more complex events, such as shared history between populations. However, the correlation structure between FLK tests can be recovered by decomposing the FLK signal into loadings on orthogonal components, as explained in supporting information file 1.2 of Fariello et al. [24]. Thus, rather than using a LD correlation matrix for SNPs in CAVIAR, we used the eigen-decomposition based correlation matrix between SNPs selected for the analysis. We then ran CAVIAR by allowing up to 10 associated variants in the region and extracted the posterior mean number of associated variants as a measure of its allelic heterogeneity.

### Additional polymorphism discovery and genotyping at the MC1R locus

The *MC1R* locus (OAR14: 14,228,283–14,235,506 on OARv3.1 assembly) was amplified as overlapping PCR fragments using appropriate PCR conditions regarding the expected length of the product: either conventional amplification using goTaq polymerasae (Promega) or Long-range PCR amplifications using the Long PCR Enzyme Mix provided by Fermentas (https://www.thermofisher.com/us/en/home/brands/thermo-thermo-scientific/molecular-biology/thermo-scientific-molecular-biology-products/fermentas.html) was performed, using 50 ng of genomic DNA as a template and the manufacturer’s protocol. After treatment with 0.5 U of Tsap (Promega) and 10 U of exonucleaseI (Biolabs), 10 to 90 ng of PCR product were used for sequencing with either internal primers or PCR primers used for the amplification. Sequencing reactions were carried out via the BigDye® Terminator v3.1 Cycle Sequencing Kit (http://www.appliedbiosystems.com). The primers are listed in Additional file 5. Sequences from three animals from each breed exhibiting a selection signature were aligned against scaffold 00839 of the *Ovis aries musimon* assembly (GenBank accession HG925721.1), which we found of better quality than the OAR3.1 reference genome, using CLC software and allowed the detection of polymorphisms. The discovered polymorphisms were genotyped for all the animals of the relevant breeds by sequencing purified PCR products with internal primers using the same protocol. The sheep genome browser from EBI (www.ensembl.org) was used to determine conserved regions through 39 eutherian mammals and calculate GERP scores.

## Results

### Population structure of French sheep

We inferred population structure in sheep breeds that represent the majority of commercial breeds plus some conserved breeds in France using three different approaches (see methods): a principal component analysis (PCA) based on the genotype matrix, an unsupervised clustering approach based on Hardy Weinberg Equilibrium within clusters, similar to the STRUCTURE model [27] but with better computational properties on large samples [19] and a model-based approach aimed at reconstructing a population tree with possible admixture events [20].

**Fig. 1:**
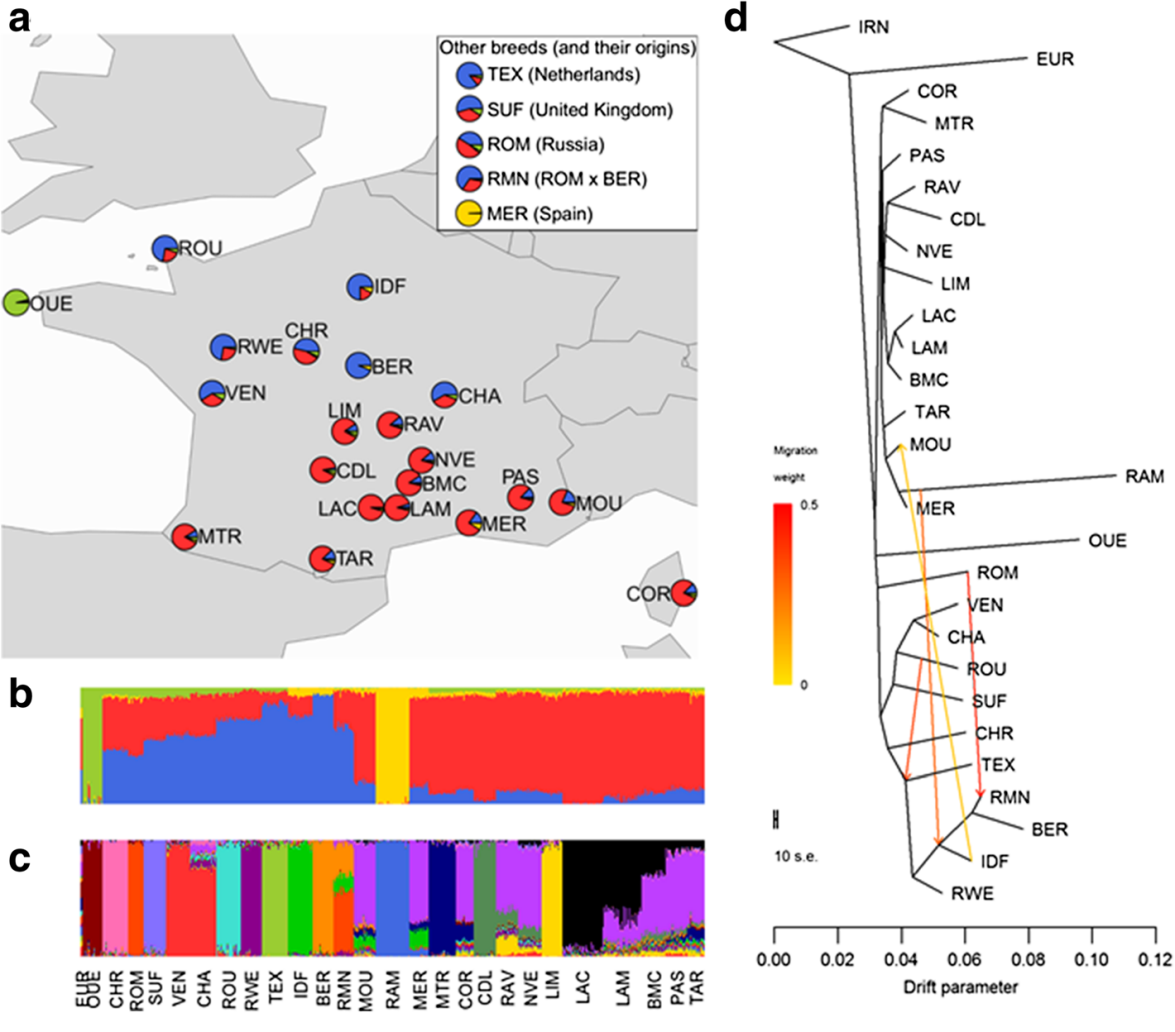
Population structure of French sheep.** a** Average individual ancestry coefficients within breed for four ancestral populations. Breeds are mapped to their geographical origin. Individual ancestry coefficients for (**b**) four ancestral populations and **c** 16 ancestral populations. **d** Maximum likelihood population tree with four migration events

To get insight into the domestication history of the breeds, we included the Asian Mouflon and the European Mouflon as outgroup populations in our analyses. Allele frequencies in the Asian Mouflon, the ancestor of domesticated sheep [28, 29], were used to root the population tree in the maximum likelihood tree analyses. Two genotyped samples of European Mouflon from Corsica were included in the principal component, individual ancestry coefficient and maximum likelihood tree analyses. The placement of the European Mouflon sheep on the maximum likelihood tree analyses (Fig. 1d), after the Asian Mouflon sheep but before any other domesticated breeds, was consistent with the known origin of this population: European Mouflon sheep were domesticated sheep that became feral early after their arrival to Europe and can still be found on Corsica and Sardinia islands in the Mediterranean today [30]. Because the European Mouflon sheep appeared on the tree before any of the French sheep, we used these two animals in selective sweep studies as an outgroup to root the population tree.

The results obtained with the different analytical approaches complemented each other and showed definite structure in French sheep: northern breeds, southern breeds and two highly differentiated breeds (the Ouessant (Ushant) and Mérinos de Rambouillet breeds). Northern breeds had well defined cluster assignment in PCA analysis (Additional file 6), were clearly differentiated from other breeds in the estimation of individual ancestry coefficients analysis and had long branch length in the estimated population tree indicating higher drift and differentiation between breeds. In contrast, southern breeds clusters were very closely grouped in the PCA, had subtler individual clustering in the estimation of individual ancestry coefficients analysis and short branch length in the estimated population tree. Northern and southern breed grouping could be seen in K = 4 (Fig. 1) (where K is the number of ancestral populations). The estimated population tree that best explained genetic relationships between breeds had four mixture events: a mixture event from Romanov to Romane breeds; a mixture event from Mérinos de Rambouillet to the branch including Île de France and Berrichon du Cher sheep; a mixture event from Roussin de la Hague to the branch with Texel, Rouge de l’Ouest, Île de France and Berrichon du Cher sheep; and a mixture event from Île de France to Mourerous sheep. These mixture events were confirmed with f3 tests (Additional file 7).

### Population structure of European sheep

**Fig. 2:**
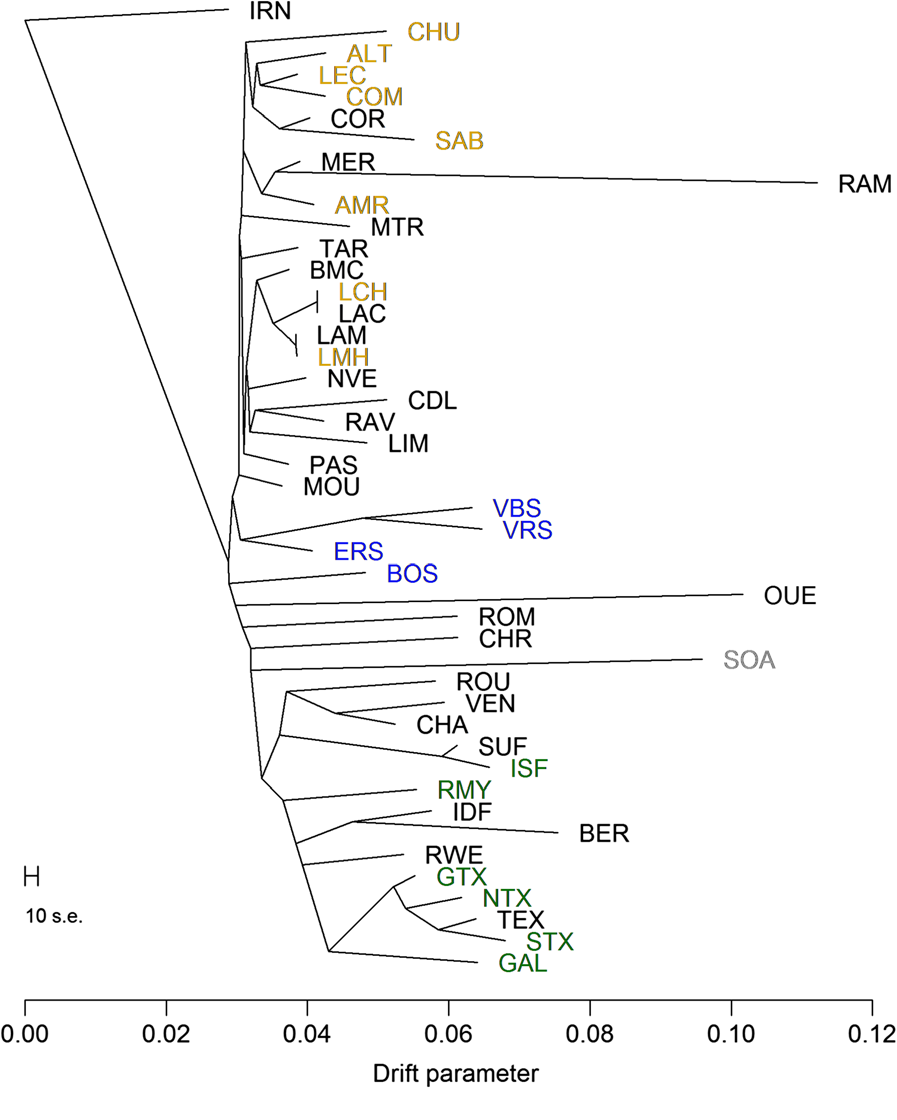
Maximum likelihood population tree of European sheep breeds estimated using 40 K SNPs**.** Breeds from this study dataset are in black. Sheep HapMap breeds are from Northern Europe (in grey), Britain (in green), Switzerland (in blue) and West and Middle Mediterranean (in gold)

To place the population structure of French sheep into the context of European population diversity, population trees with mixture events and individual ancestry coefficients were estimated using European sheep breeds from the Sheep HapMap project and included the French dataset downscaled to medium density SNP information. The maximum likelihood population tree with no migration events (Fig. 2) showed that French sheep complemented the Sheep HapMap dataset in the European sheep population tree. Most of the breeds in our dataset added to the global diversity, as they tended to root in internal branches of the population tree, either within northern or southern breeds (e.g. the Berrichon du Cher/Île de France and Causse du Lot/Rava/Limousine respectively) or at the basis of the population tree (e.g. the Ouessant or Romanov breeds). The other French breeds branched within population groups that are already present in the Sheep HapMap: the Corsican breed is closely related to Italian breeds, and the Sardinian Ancestral Black in particular; the other cases corresponded to breeds that are also found in other countries and the population tree reflects the diversity within these breeds between countries (examples include: the French and Irish Suffolk populations; the German, Scottish, New Zealand and French Texel sheep populations; and the Australian Merino, Mérinos d’Arles and Mérinos de Rambouillet breeds).

As when considering French breeds only, the tree obtained from the inclusion of the Sheep HapMap breeds separated into: southern breeds, which included Spanish and Italian breeds; and northern breeds. Here also, the northern breeds tended to have longer branch lengths and more drift than southern breeds. When estimating ancestral populations at K = 3 there was differentiation between northern European and southern European sheep breeds similar to the analysis with only French breeds at four ancestral populations (K = 4).

### FLK and hapFLK genome scans

We did not consider four of the breeds for our selection scan: three breeds, the Ouessant, Mérinos de Rambouillet and Berrichon du Cher, that have experienced severe bottlenecks, corresponding to very long branch lengths in the population tree (Fig. 1d); and the Romane breed, as it is a recent composite of two breeds.

We detected selective sweeps with all breeds taken together and separately in northern and southern sheep breeds using both FLK and hapFLK (results in Additional file 4). When considering all breeds together, we detected 50 selection signatures. Three of these signatures were only found when analysing all the breeds together, while the other 47 were also found in the within group analyses. These three specific signatures were on chromosome 3, between 129.0 Mb and 130.7 Mb; on chromosome 8, between 80.6 Mb and 83.1 Mb; and on chromosome 24 between 9.9 and 10.6 Mb (see Additional file 8 for detailed figures of all selection signatures).

As only three signatures were specific to analysing all breeds together, we then focused our inference on the analyses performed in each group separately. We had more power to detect signatures of selection when separating breeds into the two groups: for FLK and hapFLK we detected 61 and 26 regions respectively for northern sheep populations and 65 and 42 regions for southern sheep populations. The average size of signatures detected was 1.7 Mbp and they ranged from 23Kb to 22 Mbp in length.

Based on the FLK and hapFLK results, we defined selection signatures as intervals where nearby SNPs were significant (see Materials and Methods). We then listed all the genes in the regions, and in some cases could pinpoint a list of likely candidate genes, based on their distance to the most significant SNP and on previous literature on their functions or selective status. This list of candidate genes includes many genes known to be associated with agricultural and adaptive importance. Many of the regions containing these genes are examples of allelic heterogeneity in selection signatures, including *ADAMST9*, *MSRB3*, *SOCS2*, *RXFP2*, *ABCG2*/*NCAPG*/*LCORL* and *MC1R*.

Initially, we found a large number of selection signatures on chromosome 2, surrounding the myostatin gene. As the Texel population have experienced a large loss of genetic diversity in this region, we hypothesized that some of these signatures could be due to a very long selected haplotype in the Texel breed. Therefore, we re-analysed chromosome 2 with both northern and southern breeds but excluding Texel sheep. The selection signatures found in this analysis are shown in Additional file 9. In contrast to FLK results where all animals were included, there were five fewer regions detected. These five regions spanned from 109.0 to 122.3 Mbp, which included the *MSTN* gene. All the other selection signatures on chromosome 2 were still found. Therefore those five regions should be considered as one single signature with the Texel selected haplotype stretching from 109.0 to 122.3 Mbp. We still find evidence for selection in this new analysis in the Rouge de l’Ouest breed around the *MSTN* gene, but not associated to fixation of the allele, the signal ranging from 117.9 to 118.8 Mbp.

### Quantifying allelic heterogeneity

**Fig. 3:**
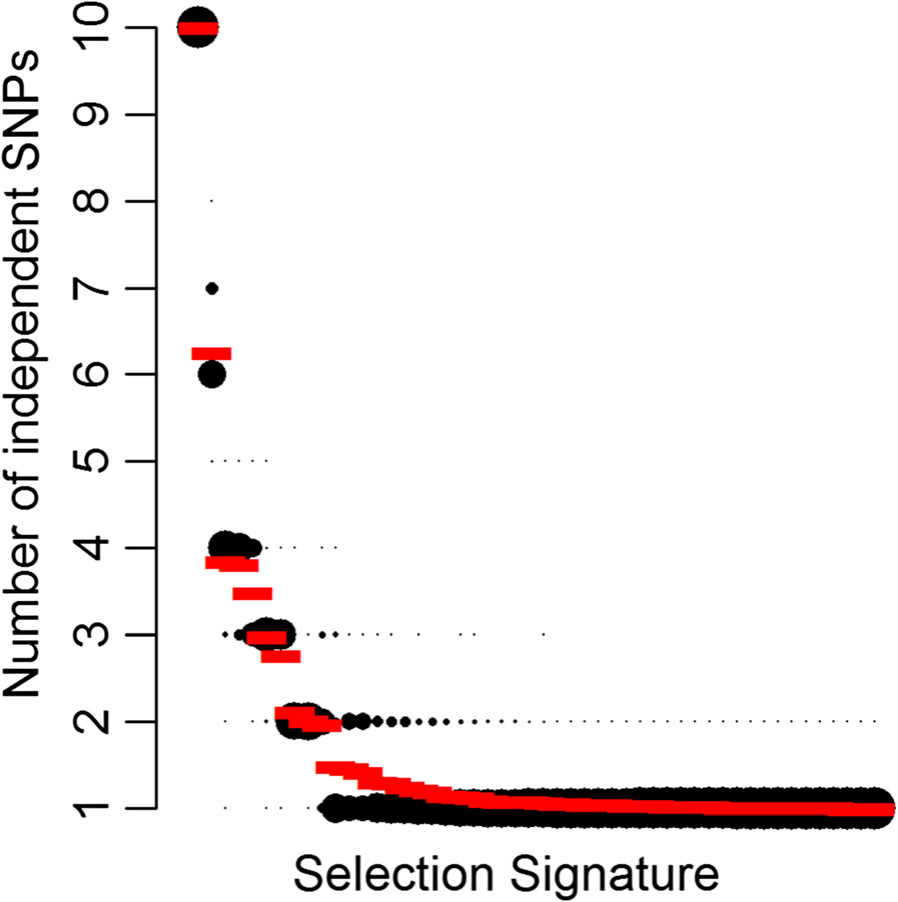
Posterior probabilities of the number of independent SNPs in selection signatures. Posterior mean numbers of independent variants are indicated in red. Selection signatures included in this analysis were detected using FLK and all French breeds

We tested for allelic heterogeneity by adapting a method initially developed for genome-wide association study, implemented in the software program CAVIAR [26] (see Methods). We tested up to 10 independent variants in each region under selection (for regions detected using FLK for northern and southern breeds together only). The posterior probabilities for one to 10 independent SNPs were plotted for all 42 signatures of selection in Fig. 3. Out of 42 regions, 10 had more than one SNP associated and therefore had evidence of allelic heterogeneity. The 10 regions, their candidate genes and the number of independent SNPs are reported in Additional file 10. Visual inspection of the local trees and haplotype frequency cluster plots (Additional file 11) from these 10 regions also showed evidence of allelic heterogeneity.

### Additional polymorphism discovery and large-scale genotyping at MC1R locus

**Fig. 4:**
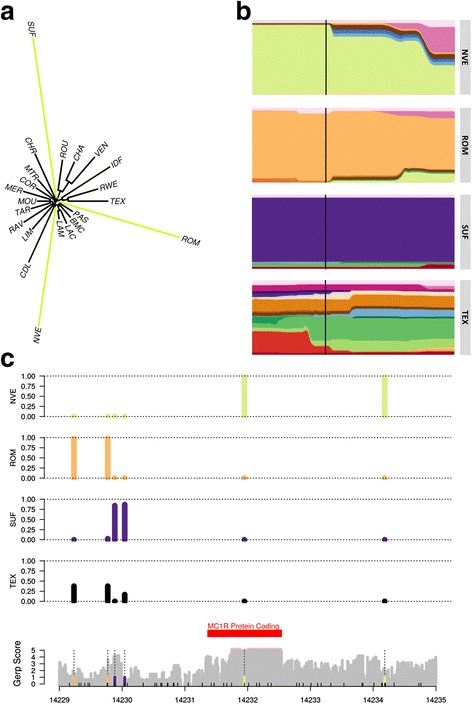
Chromosome 14:14069040 to 14530851. **a** Local tree and **b** haplotype cluster frequency plots for Noire du Velay (NVE), Romanov (ROM), Suffolk (SUF) and Texel (TEX) breeds for chromosome 14:14,069,040 to 14,530,851 (with the position of *MC1R* marked with the black line) and **c** mutation location and their frequencies in Noire du Velay (NVE), Romanov (ROM), Suffolk (SUF) and Texel (TEX) breeds

Our analysis revealed that selection in the region encompassing the *MC1R* gene, a gene associated with pigmentation, affected the only three black-faced breeds in our dataset: the Romanov, the Suffolk and the Noire du Velay (Fig. 4a, b). Furthermore, haplotype diversity plots showed they were selected on different haplotypes (Fig. 4b). To test whether these three breeds were selected not only on different haplotypes but also on different mutations in *MC1R* we re-sequenced individuals from these breeds as well as a breed not found to be selected on in this region, the Texel.

Sequencing of 6894 bp of the *MC1R* region and genotyping of the Noire du Velay, Romanov, Suffolk and Texel breeds allowed us to generate a reference sequence (EMBL accession number LT594967) with 2 remaining N blocks of 270 and 261 nucleotides in the 5′ region. It exhibits a unique open reading frame. We identified a potential TATA box with a “TATAAA” motif (at positions 14,229,176–14,229,181), 2522 bp before the start codon and a potential polyadenylation signal “AATAAA” (at positions 14,233,015–14,233,020). Therefore we can assume that the 5’UTR of the sheep *MC1R* transcript is about 2500 bp long and the 3’UTR around 500 bp long. The deduced *MC1R* sheep protein is 317 amino acids.

Our sequences allowed us to detect and further genotype six SNPs and one 11 bp insertion (Fig. 4c) (dbSNP accession numbers ss# 1996900605 to 1996900611). We found that the three breeds were indeed almost completely fixed around *MC1R*, and that the three main breed haplotypes were different. The Noire du Velay re-sequenced individuals were all homozygous in the region and carriers of the two known mutations for dominant black in sheep. The Romanov individuals were all homozygous for the same haplotype in the region, but did not carry the two known mutations above. We identified two mutations that are not found in Noire du Velay or Suffolk. However, they were found at intermediate frequency in Texel, a breed where all individuals are white. Finally, the Suffolk breed exhibits yet another allele frequency profile different from the two other black-faced breeds. We found two mutations of high frequency in the Suffolk, one of which (rs406233740) is a SNP at position 14,229,883 that is not found in any of the other breeds and lies in a region of elevated GERP score, i.e. highly conserved among mammals.

## Discussion

In this study, we performed a genome scan for selection in a livestock species, sheep, that has adapted to various adaptive constraints imposed by its colonization of new environments and its breeding for agricultural purposes. Below, we discuss to what extent the evolutionary history of European sheep populations and that of their identified adaptive loci can be inferred from the genomic signatures left by drift and selection.

### French sheep populations and the meeting of European sheep domestication routes

Our dataset provided a new sample of populations from European sheep that we have shown to complement the already available samples from the Sheep HapMap project. Our analysis of population structure in French, and more generally European sheep, highlighted how populations have been established in the continent since sheep domestication. In domesticated samples, the three methodological approaches we used showed a clear structure (Fig. 1) in two main groups of breeds plus two highly differentiated breeds, the Ouessant (also called Ushant) sheep and the Mérinos de Rambouillet. The Ouessant sheep breed has historically been isolated and consequently has accumulated substantial drift. Its position in the tree is consistent with it having a distant origin from the other breeds in the data set. The Ouessant breed possibly originated from a first wave of colonization after domestication, as has been shown for other ancient European breeds of sheep such as the Soay sheep [31]. However, its positioning in the population tree might not be very reliable due its long branch length. The other highly inbred breed is the Mérinos de Rambouillet. This breed consists of a single flock of animals that has been raised without the introduction of additional animals since 1786. Although it has a very long branch in the population tree, it clusters with the other Merino population in the dataset, the Mérinos d’Arles.

Apart from the Ouessant and Mérinos de Rambouillet, the populations were divided into two main groups, corresponding to northern and southern origins (Figs. 1 and 2). The two groups present contrasting structure. In the northern group, breeds were clearly separated on the PCA analysis (Additional file 6), individuals had clear cluster assignment (Fig. 1b) and tended to have longer branch length in the population tree (Fig. 1d), indicating higher drift and differentiation between breeds. In contrast, breeds of southern origin showed less differentiation, with PCA clusters being closer, individual clustering subtler and shorter branch length in the population tree. This pattern is particularly clear for breeds originating in the Massif Central (Blanche du Massif Central, both Lacaune populations, and Noire du Velay breeds), but is also true of breeds more geographically distant such as the Manech Tête Rousse and the Préalpes du Sud breeds. Their longer established breeding programs could explain the longer branch lengths of the northern populations compared to southern ones. Apart from this clear geographical structure, the population tree that best explained genetic relationships between breeds had four migration events (Fig. 1D). The migration event with the most weight was from the Romanov to the Romane, which was on the same branch as the Berrichon du Cher. This migration edge is explained by very recent admixture: the Romane breed was created in France in the 1960s by crossing Romanov and Berrichon du Cher animals. The migration edge from the Mérinos de Rambouillet branch to the branch of both Île de France and Berrichon du Cher breeds is likely representative of the crossing of these breeds with Merinos in the nineteenth century to improve wool quality [32]. The migration edge from the Roussin de la Hague branch to the branch with Texel, Rouge de l’Ouest, Île de France and Berrichon du Cher breeds could come from the use of British breeds to crossbreed in the eighteenth century to improve meat production [32]. However, this is difficult to confirm because the main British breeds involved, the Leicester Longwool and Southdown, are not part of our dataset nor of the Sheep HapMap dataset. Finally, the migration branch from the Île de France to Mourerous breed was an unexpected result and might be due to some purported recent crossing of northern breeds with Mourerous sheep to improve meat production. Overall, the pattern of genetic diversity that emerges from our analysis is that of three main lineages for the European populations, an ancestral lineage that is found today in the Ouessant, the Romanov and the Soay breeds and two largely separated geographical lineages (north and south) of modern domestic sheep, with evidence for punctual admixture events between northern and southern populations. It is tempting to relate these two main lineages to the two known domestication routes (Danubian and Mediterranean), but further studies are needed to formally test this hypothesis, possibly exploiting ancient DNA information.

### Selection signatures are mostly detected within geographical groups

We did not find many signatures of selection between the two distinct genetic pools of populations. The only three of signatures of selection found when analysing all the breeds together were found on chromosome 3, between 129.0 Mb and 130.7 Mb; on chromosome 8, between 80.6 Mb and 83.1 Mb; and on chromosome 24 between 9.9 and 10.6 Mb (see Additional file 8 for detailed figures of all selection signatures). The signature on chromosome 3 harbours the *SOCS2* gene recently shown to carry a mutation with adverse pleiotropic effects, positive for growth and negative for mastitis [33]. The pattern of haplotype diversity showed that a haplotype cluster segregated at moderate frequency in many northern breeds (light yellow on page 13 from Additional file 8), that can only be detected as outlying by including southern breeds. Moderate allele frequencies at this common haplotype in many northern breed could be due to balancing selection on adverse pleiotropic effects at *SOCS2*, although it is essentially impossible to test this hypothesis from SNP array data alone. Analysis of the region on chromosome 8 is consistent with selection in Île de France, although the selected haplotype cluster has not reached complete fixation in this breed. It was also found at low frequency in the Texel and Rouge de l’Ouest populations, closely related to Île de France. Hence, when analysing only the northern breeds, this signal can still be consistent with drift alone. However, as none of the southern breeds harboured this haplotype, haplotype diversity patterns in northern breed become unlikely due to drift alone. Finally, the region on chromosome 24 exhibited a very low haplotype diversity in most northern and southern breeds, with a similar haplotype segregating at high frequencies in many breeds (i.e. low differentiation due to general selection on the same haplotype). Only a few northern breeds had some more elevated haplotype diversity, which allowed detecting selection when included with the southern breed. Interestingly, the selection signature in this region looked quite widespread for the same haplotype in most of the breeds in the dataset. These three cases illustrate how increasing the number of populations interrogated in genome scans for selection can increase detection power. In our case however, the few selection signatures detected that are due to differential selection between the two groups show that drift, i.e. independent evolutionary history between the northern and southern groups, still remains the most parsimonious explanation for their genetic differences. It is still possible that adaptive effects that were undetected in our analysis have contributed to these differences. In particular polygenic selection on complex adaptive traits would need to be tested. This would require, however, additional data on these possible traits and their variation between sheep populations. Admixed breeds between the two groups, such as the Romane breed could offer suitable models for such studies.

**Fig. 5:**
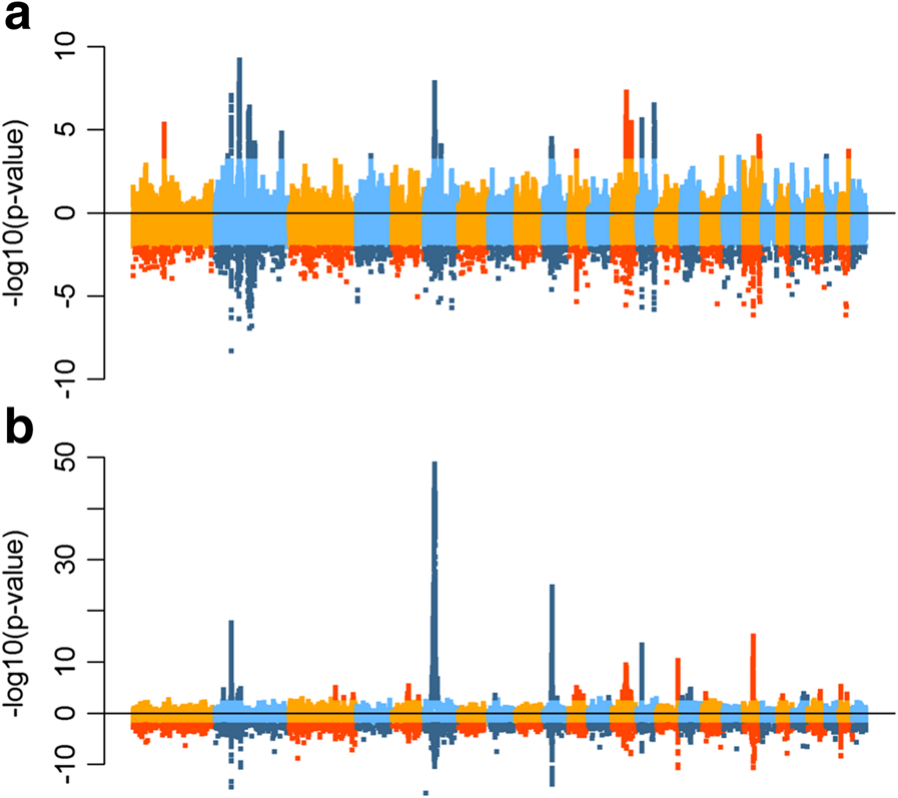
Manhattan plots of hapFLK and FLK results in northern (**a**) and southern (**b**) populations. In each panel, the positive values are the -log10(*p*-value) of the hapFLK test and the negative values are the log10(p-value) of the FLK test. Significant SNPs (FDR < 0.05 for hapFLK and < 0.01 for FLK) are shown in darker colours

As only three signatures were specific to analysing all breeds together, we then focused our inference on the analyses performed in each group separately. We had more power to detect signatures of selection when separating breeds into the two groups: for FLK and hapFLK we detected 61 and 26 regions respectively for northern sheep populations and 65 and 42 regions for southern sheep populations. As shown above, southern French sheep are not highly differentiated from each other and have experienced relatively low drift compared to northern ones. Because of this, we had more power in southern than in northern populations, which could explain the detection of more regions under selection and much smaller *p*-values in the former than in the latter (Fig. 5).

With the high density SNP information, selective sweep regions were on average smaller than those detected by Fariello et al. [14] using medium density (50 K) SNP information. While we acknowledge that it is difficult to make comparisons with Fariello et al. [14] because their study included different sheep populations, regions detected with the 50 K SNP array were 2.8 times larger than those that were detected with the 600 K array (Additional file 11). For those regions detected in both studies, the regions detected with 50 K were 1.7 times the size of those detected with 600 K SNPs. The higher resolution obtained can come from the fact that we had a better haplotype diversity description with higher SNP density and were able to reduce the size of the candidate region by analysing more breeds showing more recombinant haplotypes.

### Overview of candidate genes found in selection signatures

Candidate genes found nearby significant SNPs for FLK and hapFLK analyses include genes for coat colour (*ASIP*, *MC1R*, *TYRP1*, *MITF*, *EDN3*, and *BNC2*), stature and morphology (*NPR2*, *MSTN* (*GDF-8*), *LCORL* and *NCAPG*, *ALX4* and *EXT2*, *PALLD*), milk production (*ABCG2*), horns (*RXFP2*) and wool (*IRF2BP2*) [6, 24, 34]. Other candidate genes that we identified included *SOCS2*, associated with growth and mammary gland development [33], *OXCT1*, associated with milk fatty acid traits in dairy cattle [35], EBF2, involved in brown fat fate and function in mice [36], *ADAMTS9*, under selection in Tibetan pigs and boars living at high and moderate altitudes and *MSRB3*, associated with floppy ear position in dogs [37]. We further discuss some of these selection signatures with candidate genes that have had their function in mammals discussed in peer-reviewed literature and are no further than 150 kbp from the SNP with the smallest *p* value for FLK or hapFLK tests in the region.

### Retracing the origin of a selected allele: Example of the MSTN signature

One of the most widely recognized selection signature in sheep is the selection for a mutation in the myostatin gene in the Texel breed. Indeed, the actual causal mutation is a SNP that has been placed on the HD SNP array used in our study (rs408469734). This offered a great opportunity to get insights into the history of the mutation in the populations considered here. First, we could confirm that the mutation was the SNP showing the most significant FLK signal of the corresponding selection signature, and local population trees highlighted the Texel breed as being the selected population (page 10 of Additional file 8). We could determine that the Texel selected haplotype is very long, likely stretching from 109.0 to 122.3 Mbp, indicating a very strong and recent selective sweep. But we also found this SNP segregating in another meat breed, the Rouge de l’Ouest. After removing the Texel breed from the analysis, there was clear evidence that the mutation was also selected in the Rouge de l’Ouest but was not accompanied by fixation of the allele in this population. The origin of the mutation in the Rouge de l’Ouest could have two explanations: that it was introgressed from a recent Texel population or that it was already present in the ancestral population to the Texel and the Rouge de l’Ouest. To decide between the two hypotheses, we studied the haplotype diversity pattern in the two breeds; while the Texel show a single very long haplotype carrying the mutation, it appeared that the Rouge de l’Ouest exhibited different haplotypes carrying the mutations. This is more consistent with an ancestral origin of the mutation, which was recently highly selected in the Texel while managed in a less intensive way in the Rouge de l’Ouest population, producing a hard-sweep signature in the former breed and a soft-sweep signature in the latter.

### Allelic heterogeneity in selection signatures highlights adaptive hotspots

We define allelic heterogeneity as the presence of multiple selected alleles, or haplotypes, at the same genomic location. In our analyses, we identified allelic heterogeneity in a selection signature when we had evidence for (i) more than one breed affected by selection in the same region and (ii) different haplotypes having arisen to high frequency in the selected breeds. Specifically, we identified regions with evidence for allelic heterogeneity by combining two criteria. First, we adapted the CAVIAR method [26] (see Methods) to determine selection signatures where the estimated number of independent SNPs under selection was at least two in the FLK analysis that included all animals (Fig. 3). Then, we visually inspected the haplotype cluster frequency plots and local trees for regions under selection (Additional file 8) and chose to confirm allelic heterogeneity when the local trees indicated more than two breeds under selection and the corresponding haplotype cluster frequency plot indicated more than one haplotype with high frequency in the set of selected populations. We confirmed allelic heterogeneity in 10 selective sweeps (Additional file 10), including regions containing genes shown to be involved in agriculturally important traits such as *LCORL, MSRB3, MC1R, SOCS2, RXFP2,* and *ADAMST9* (discussed below).

Given our criteria, the allelic heterogeneity we identified can be due to different underlying phenomena. Firstly, the identified heterogeneity could come from independent selection on the same gene but for different mutations in different populations. Secondly, given that the regions detected usually span several genes, it is possible that a heterogeneous signature could come from selection on different genes, and thus different evolutionary pressures. In this case, it also implies that a selective pressure on one gene has impacted the genetic diversity on nearby genes that are adaptive in other populations due to physical linkage. Hence, this selection can have pleiotropic effects on fitness. Finally, because we do not have full sequence data in our samples, the heterogeneity discovered with CAVIAR could be due to insufficient linkage disequilibrium between single markers and the underlying causal mutation, i.e. there is only one underlying causal polymorphism but multiple SNPs are needed to capture it. However, we consider this last possibility usually unlikely given that we used high-density genotyping (one marker every 5Kb on average) and relatively closely related populations. In the following, we discuss some of the signatures with a clear signal of allelic heterogeneity and show how genetic patterns can be used to propose an evolutionary history for their adaptive alleles.

### Selection signatures with multiple adaptive loci

**Fig. 6:**
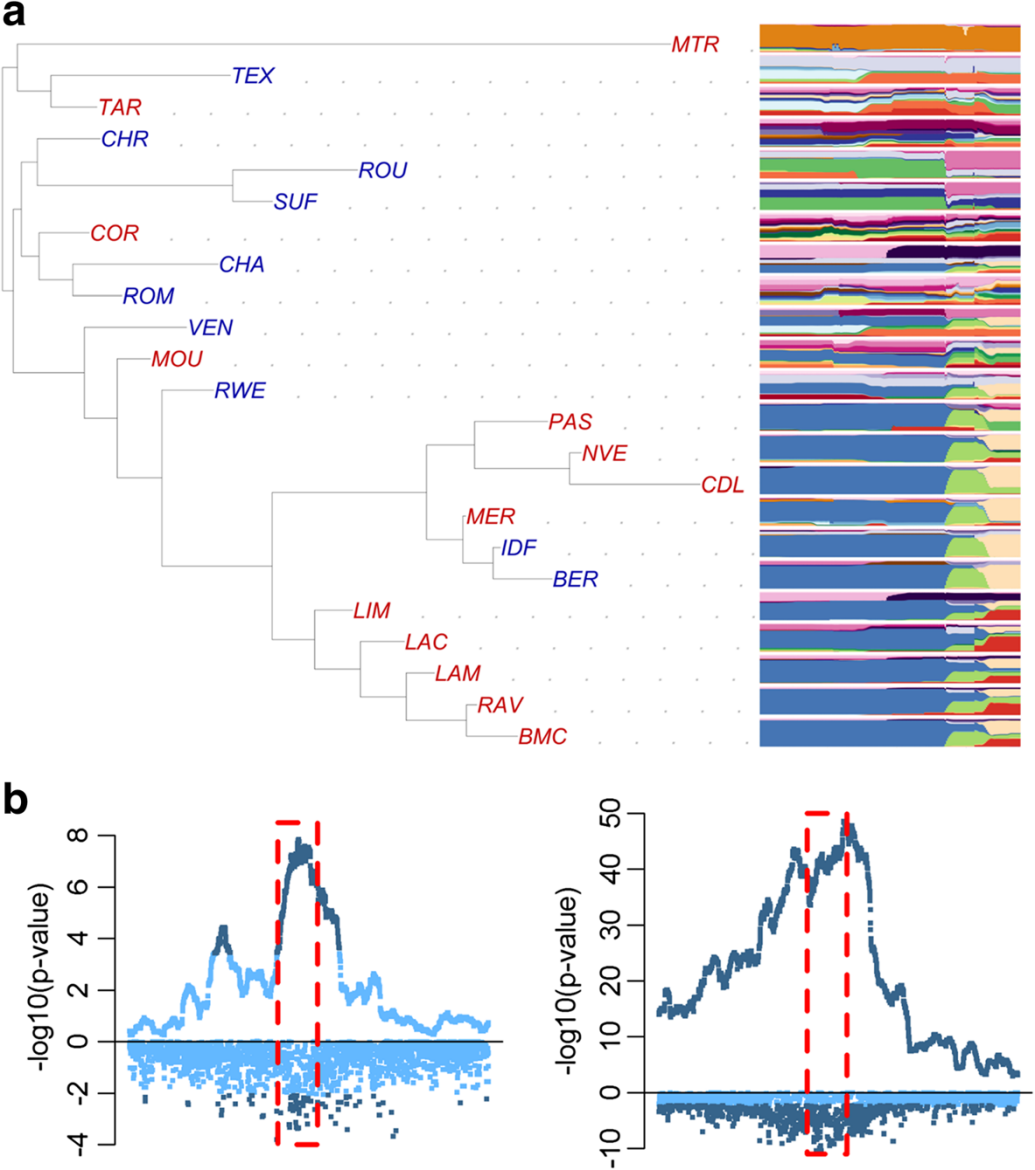
Selection signature around genes *ABCG2*, *NCAPG* and *LCORL* on chromosome 6. **a** Population tree (left) constructed in the region under selection with haplotype cluster plots shown for each breed (right). hapFLK and FLK *p*-values for the selection signature in the northern (**b**) and southern (**c**) populations

The largest signal found in our analysis corresponds to a region that contains two genes known to be associated with agriculturally important traits. This region on chromosome 6 showed clear allelic heterogeneity and was an example of selection on a genome region introgressed after an admixture event (page 17 of Additional file 8). In a previous scan for selection signatures with 50 K SNPs, a similar signature was detected in the same region (between 33.22 to 41.02 Mbp) and the *ABCG2* (OAR6:36,514,210–36,556,824) and *NCAPG/LCORL* (OAR6:37,256,548–37,333,851/OAR6:37,365,236–37,452,332) genes were identified as likely candidate genes in this region [14]. In our study the signature in this region is still large (between 22.85 to 48.63 Mbp), however the most significant single SNPs tests were located in a narrower region of about 2 Mbp (between 35.76 and 37.81 Mbp). We found two clearly distinct haplotypes selected on in this region, one seen only in the Manech Tête Rousse breed and one seen in southern breeds as well as in two northern breeds, the Berrichon du Cher and the Île de France (Fig. 1a). As shown in our analysis of genetic diversity, both these northern breeds have a history of ancient crossbreeding with a Merino-related population (Fig. 1d). When reconstructing a population tree using only SNPs present in this selection signature, these two breeds actually cluster with the Mérinos d’Arles (Fig. 6) within the southern group. Our explanation is that a selected mutation originating from a southern population related to the Merino has been introgressed and selected again in these two northern breeds, thereby producing by far the strongest signal of selection in our analysis. As this selection event affected a large genome region, it is hard to determine which of the two candidate loci has been targeted by selection. Indeed *ABCG2* is associated with milk production traits in cattle [38] and sheep [39, 40] while *NCAPG* and *LCORL* are associated with growth and height related traits in cattle [41], chickens [42], horses [43], humans [44], pigs [45], rabbits [46] and sheep [47, 48]. It is difficult to determine which of the *NCAPG* or *LCORL* gene is causative for these traits because the region exhibits elevated linkage disequilibrium in these species. In the Manech Tête Rousse, a hardy dairy breed used for cheese production in the Pyrenees region, *ABCG2* could be considered a better candidate. For the other, introgressed, haplotype, the fact that the region has been under selection in both meat and milk breed could favour the *NCAPG*/*LCORL* locus as the underlying target. Finally, the CAVIAR analysis selected four SNPs to explain the signal: two of which were closer to *ABCG2* (OAR6:35,847,708 and OAR6:35,762,202), one was closer to *LCORL* (OAR6: 37,236,177) and one was some distance from both genes (OAR6:45,495,598).

Another example of allelic heterogeneity caused by different genes being under selection close together is a region on chromosome 3 (page 14 of Additional file 8). The best SNPs from FLK tests of all sheep and northern sheep were closest to the gene *MSRB3*. *MSRB3* is in a region that has been associated with ear position (floppiness) in dogs [37, 49, 50] and ear size in pigs [51, 52]. In a study of selection signatures in Chinese sheep, researchers detected selection in the breed with the largest ears in their study [53]. In our study, there was a hard sweep in Suffolk in this region and a few other haplotypes at high frequency in the Romanov and Charolais breeds. The breed definition for Suffolk sheep in France from the national organization for selection (GEODE) describes ears as long, thin and facing in a downwards direction [54]. Hence, our results suggest that mutations in *MSRB3* could cause this breed’s phenotype. However, in this region there is also *HGMA2* and in, for example, Suffolk sheep, the selective sweep includes *HGMA2*. This gene is associated with size in mice [55], humans [56, 57], chickens [58], horses [59, 60], dogs [61], and rabbits [46]. It is therefore a possibility that *HGMA2* may be under selection in some breeds. This could be also a case where selection on the morphological effect of *HMGA2* led to the recruitment of another mutation in *MSRB3*, leading to the particular ear phenotype of the breed, but elucidating such an effect would require sequencing data.

### Selection signatures with multiple adaptive alleles

Finally, we found a situation where allelic heterogeneity could have resulted from selection on the same phenotype at different mutations at the *MC1R* gene (page 36 of Additional file 8). *MC1R* is a gene with known causal mutations for black coat colour in sheep and in other mammals [62]. Coat colour and pattern are a result of pigmentation from melanin. Melanocytes are cells that produce melanin in granules called melanosomes [63]. Eumelanin or pheomelanin is produced depending on whether an agonist, the *melanocyte stimulating hormone* (*α-MSH*), or antagonist, the *agouti signalling protein* (*ASIP*), respectively bind to the *melanocortin 1 receptor* (*MC1R*) [64]. The melanosomes transfer melanin to keratinocytes, the most predominant cell type in the outer layer of skin, the epidermis [63]. Keratinocytes can then incorporate melanin [65]. For example, it has been shown in a study of Asiatic sheep that black individuals had only eumelanin present in their wool [66]. Our analysis revealed that selection in this region affected the only three black-faced breeds in our dataset: the Romanov, the Suffolk and the Noire du Velay. Furthermore, haplotype diversity plots showed they were clearly selected on different haplotypes (Fig. 4a, b). When we re-sequenced individuals from these breeds as well as a breed not found to be selected in this region, Texel, we found that the 5’UTR of the sheep *MC1R* transcript is about 2500 bp long and the 3’UTR around 500 bp long. Few data are present for mammalian 5’UTR of *MC1R* transcripts however the NM_002386.3 human transcript also shows a long 5’UTR (1380 bp) and a 766 bp 3’UTR. The deduced *MC1R* sheep protein is 317 amino acids, the same number as in cattle (*Bos Taurus*) with a 96% identity between the two proteins. We found that the Romanov, the Suffolk and the Noire du Velay were indeed almost completely fixed around *MC1R*, and that the three main breed haplotypes were completely different (Additional files 12 and 13). The Noire du Velay re-sequenced individuals were homozygous for the two known mutations for dominant black in sheep. These mutations (named c.218 T > A and c.361G > A in the literature) have been shown to be responsible for dominant black coat in sheep in Norwegian Dala, Damara, Corriedale, Spanish Merino, and Massese breeds [62, 67, 68]. The Romanov individuals were all homozygous for the same haplotype in the region, but did not carry the two known mutations above. We identified two mutations that are not found in Noire du Velay or Suffolk. However, they were found at intermediate frequency in Texel, a breed where all individuals are white. Although the presence of other coat colour modifier loci in the Texel are possible, it is maybe more likely that the Romanov mutation conferring black coat colour lies outside of the 7 Kb region sequenced here, or in the remaining N blocks of the reference sequence. Finally, the Suffolk breed exhibits yet another allele frequency profile different from the two other black-faced breeds. We found two mutations of high frequency in the Suffolk, one of which (rs406233740) is a SNP at position 14,229,883 that is not found in any of the other breeds and lies in a region of elevated GERP score, i.e. highly conserved among mammals. Consistent with the SNP array analysis, the three selected breeds showed almost no between individual variation within breeds, and carried completely different sets of mutations. Assuming *MC1R* is the causative gene, which seems highly likely given the large literature on its effect on coat colour, our results would indicate that only in the Noire du Velay breed coding sequence variants can be the causative mutations. In the two other breeds, regulatory variants are involved, possibly SNP rs406233740 in the Suffolk. CAVIAR results confirmed allelic heterogeneity and indicated six independent SNPs under selection in the study’s population.

Two other examples of multiple adaptive alleles in this dataset were found at the *RXFP2* and *SOCS2* genes. The *Relaxin-like receptor 2* (*RXFP2*) gene has been associated with the presence/absence of horns as well as horn development in different breeds of sheep and is therefore already known for its heterogeneity. In Soay sheep, horn type (horns or scurs) and horn length was associated with the same region on chromosome 10 which was mapped to a single candidate gene, *RXFP2* [69]. Later, a single SNP was found to be highly predictive for horns (dominant when inherited maternally) in Australian Merino sheep on chromosome 10 [70]. A synonymous mutation, p.P375 (c.1125A > G) in Tan and Suffolk sheep was found to be associated with the appearance or absence of horns [71]. When comparing horned and polled animals from seven Swiss breeds of sheep a 1833 bp insertion in a 4 Kb region at the 3 prime end of *RXFP2* was found only in polled animals [72]. Finally, the *RXPF2* region was detected as a signature of selection in different population groups of the Sheep HapMap dataset [6, 14]. The haplotype cluster frequency plots from those breeds under selection highlighted the complexity of selection in this region. The signal in our study was detected in all (page 28 of Additional file 8) and separate analyses of northern and southern sheep with the two most significant SNPs in the southern analyses within *RXFP2* (both intronic mutations) while the signal from hapFLK results of northern sheep peaked 89 Kb before the gene. None of the northern breeds included in our dataset are horned, and the reason for detecting a signature here is because different haplotypes have been selected on within this region. When we looked at the region extending 100 Kb on either side of *RXFP2*, while the SNP frequencies within the gene were similar among northern sheep and other polled southern sheep, in the 100 Kb before the gene there were four distinct haplotypes in polled French sheep. The software CAVIAR detected 10 independent SNPs in this region. These findings demonstrate that it is likely that multiple ancient mutations are affecting polled phenotypes rather than a case of a single mutation being shared by all polled breeds.

*Suppressor of cytokine signalling 2* (*SOCS2*) gene (page 13 of Additional file 8) is thought to play roles in metabolism, somatic growth, bone formation, the central nervous system, response to infections and mammary gland development although the main target of *SOCS2* is believed to be *GH/IGF-1* which is important for somatic growth [73]. In a grand-daughter study of 1009 commercial French dairy rams (dairy Lacaune breed) researchers found a QTL associated with somatic cell count on chromosome 3 and a highly associated SNP in *SOCS2* [33]. The frequency of this SNP in the studied population was 21.7% [33]. In contrast, in our study we detected selective sweeps in this region in mostly northern French breeds raised for meat. Considering both the literature and the results of this study, there is evidence that there is more than one functional mutation found in *SOCS2* in French sheep.

Finally, we found allelic heterogeneity in many signatures of selection for which the underlying selected phenotype is not clear. These regions identified should be studied further as they could be important for adaptation traits. An example is *ADAMTS9* (*A disintegrin-like and metalloprotease (reprolysin type) with thrombospondin type 1 motif 9*) (page 45 of Additional file 8) which is another example of one region with more than one haplotype under selection. *ADAMTS9* has been shown to have been under selection in pigs and wild boars living at high altitudes in Tibet in two studies [74, 75]. The first study was a comparison of Tibetan wild boars with domesticated southern Chinese pigs living at low altitude [74] while the other was a comparison of domesticated southern Chinese pig breeds which lived at high, moderate and low altitudes [75]. In mice, the *ADAMTS9* protease has been shown to be involved both in cardiovascular development and homeostasis [76]. In our study, selection signatures were detected in the region of *ADAMTS9* on chromosome 19 and the SNP with the lowest *p*-value for FLK analysis for all breeds was located within the gene. Two breeds were found to be under selection for this region: the Romanov and the Causse du Lot breeds. The Causse du Lot breed is a hardy breed traditionally raised in plateaus of southern France and the Romanov breed is a European north short tailed sheep breed originating in Russia and hardy in cold temperatures. This suggests that *ADAMTS9* may have a role in the ability of hardy breeds to survive in colder temperatures.

## Conclusion

In this study, we highlight the ability of selection signature studies to retrace the history of selected alleles through the use of large datasets (many individuals from many populations) and high density DNA marker information. The dataset used in this study, high density SNP genotyping of many French sheep breeds, was a powerful tool for studying adaptation and selection. We showed that French sheep populations harbour great genetic diversity, with influences from both southern and northern Europe. We detected a large set of selection signatures that we expect will foster new research on studying effects of variation of these genes on phenotypes and shed light on the history of sheep domestication and breeding in Europe. In particular, we identified selection hotspots seen in many species, introgression of adaptive alleles, and allelic heterogeneity. We identified allelic heterogeneity using a quantitative approach and confirmed the presence of allelic heterogeneity in a selection signature containing the candidate gene *MC1R* by resequencing that region in the breeds under selection. Future studies with the focus on the history of selection can be ameliorated with the use of whole genome sequencing, because sequences would contain the causal adaptive mutations, and also by including ancient DNA from past populations. Finally, we believe that the combination of this dataset with others likely to come, offers great prospects to decipher the history of animal domestication, and its relation to the human neolithic expansion.

## Additional files

Additional file 1: Cross entropy criterion. Cross entropy criterion for estimating number of ancestral populations in French breeds using 500 K SNPs. (PDF 4 kb)

Additional file 2: Fraction of the variance in the sample covariance matrix explained by the estimated sample covariance matrix. Fraction of the variance in the sample covariance matrix explained by the estimated sample covariance matrix for estimating number of migration events in the population tree of French sheep using 500 K SNPs. (PNG 6 kb)

Additional file 3: Linkage disequallibrium decay curves for a subset of populations averaged over all chromosomes. (PNG 166 kb)

Additional file 4: FLK and hapFLK results. FLK and hapFLK regions under selection for FLK and hapFLK results. (XLS 102 kb)

Additional file 5: PCR and sequencing primers for sequencing MC1R gene. (PDF 185 kb)

Additional file 6: Principal component analysis. PCA using 500 K SNP genotypes of all French breeds in this study except Mérinos de Rambouillet and Ouessant sheep. (PDF 19 kb)

Additional file 7: F3 test results for migration events detected in the population tree estimated using treemix. (PDF 111 kb)

Additional file 8: FLK results. FLK regions under selection, SNP frequencies, haplotype cluster frequencies and SNP and haplotype local trees. (PDF 14210 kb)

Additional file 9: FLK results for chromosome 2 analysis without Texel sheep. Regions under selection. (XLS 29 kb)

Additional file 10: Selection signatures with more than one independent SNP. Posterior probabilities for one to 10 independent SNPs. (XLS 26 kb)

Additional file 11: Size of signatures of selection detected. Regions detected using 50 K SNPs in blue and regions detected using 500 K SNPs in pink. (PNG 123 kb)

Additional file 12: Mutations in MC1R. Mutations found in MC1R region in Noire du Velay, Romanov and Suffolk sheep. (PDF 187 kb)

Additional file 13: Frequency of mutations in MC1R by breed. MC1R mutation frequency in Noire du Velay, Romanov, Suffolk and Texel sheep and mutation location. (PNG 40 kb)
