# Supplementary figures and images for: Revealing the selection history of adaptive loci using genome-wide scans for selection: an example from domestic sheep
Source: BMC Genomics. 2018 Jan 23;19:71. doi: 10.1186/s12864-018-4447-x (PMC5778797; doi:10.1186/s12864-018-4447-x)

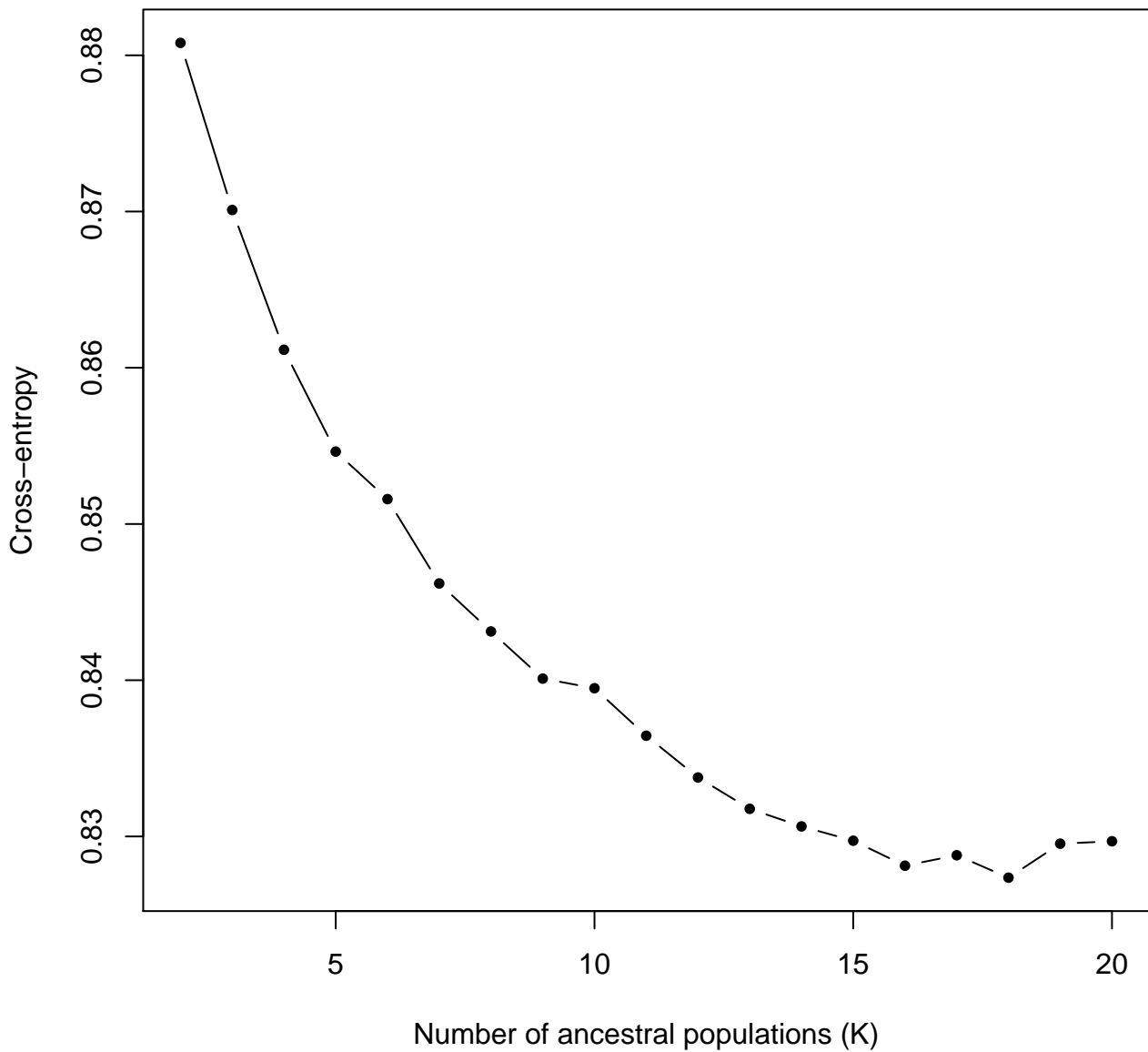

Supplement: Supplementary file 1 — Cross entropy criterion. Cross entropy criterion for estimating number of ancestral populations in French breeds using 500 K SNPs. (PDF 4 kb) [file 12864_2018_4447_MOESM1_ESM.pdf]

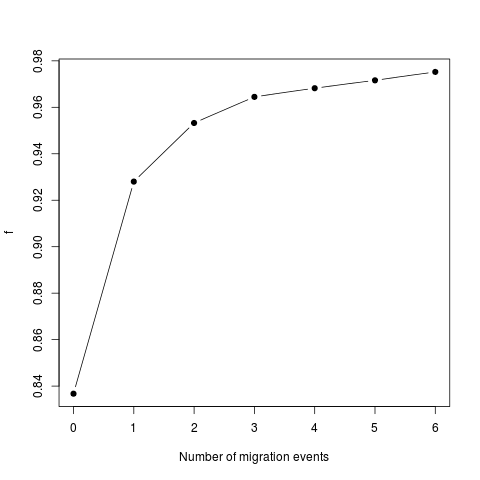

Supplement: Supplementary file 2 — Fraction of the variance in the sample covariance matrix explained by the estimated sample covariance matrix. Fraction of the variance in the sample covariance matrix explained by the estimated sample covariance matrix for estimating number of migration events in the population tree of French sheep using 500 K SNPs. (PNG 6 kb) [file 12864_2018_4447_MOESM2_ESM.png]

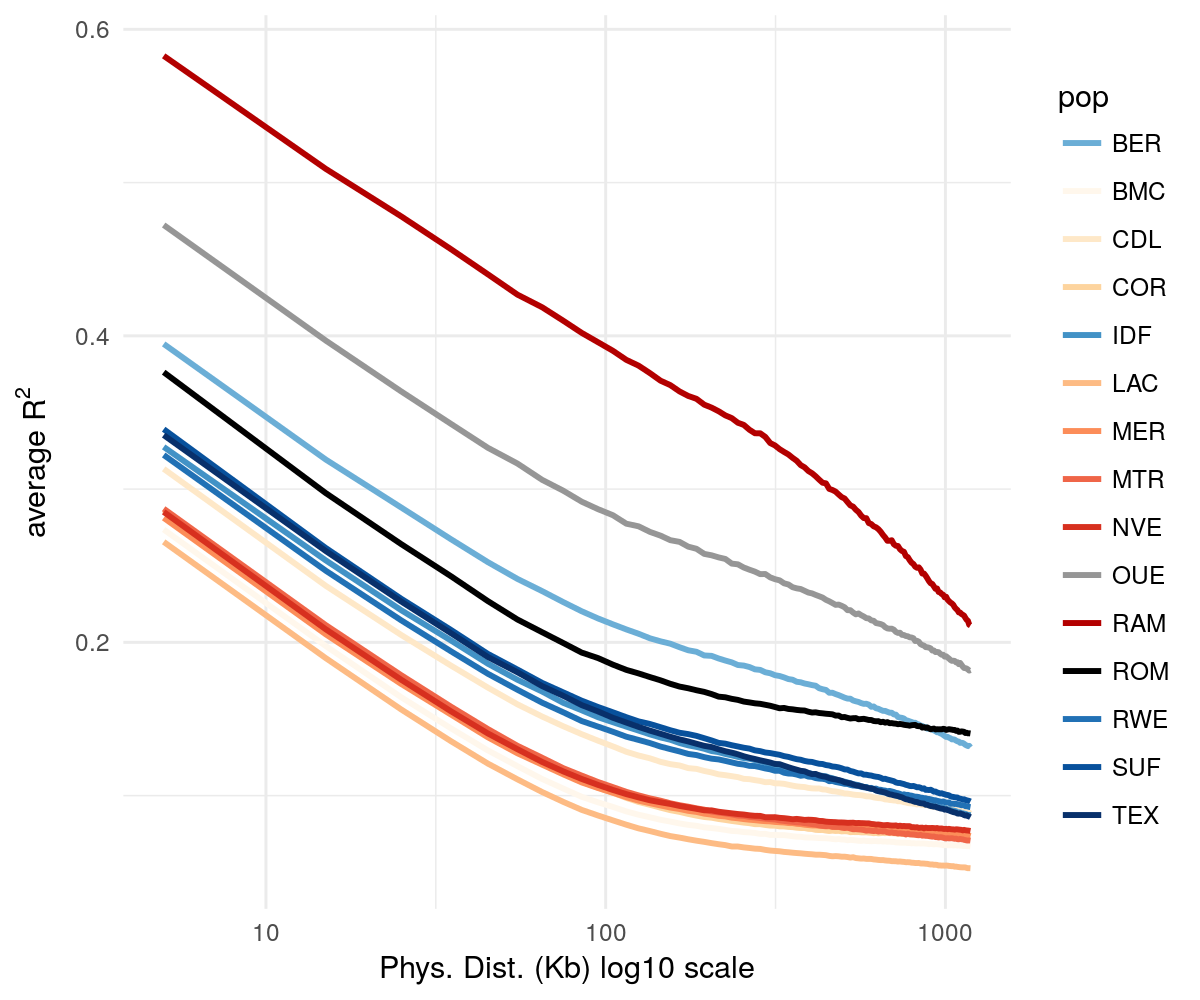

Supplement: Supplementary file 3 — Linkage disequallibrium decay curves for a subset of populations averaged over all chromosomes. (PNG 166 kb) [file 12864_2018_4447_MOESM3_ESM.png]

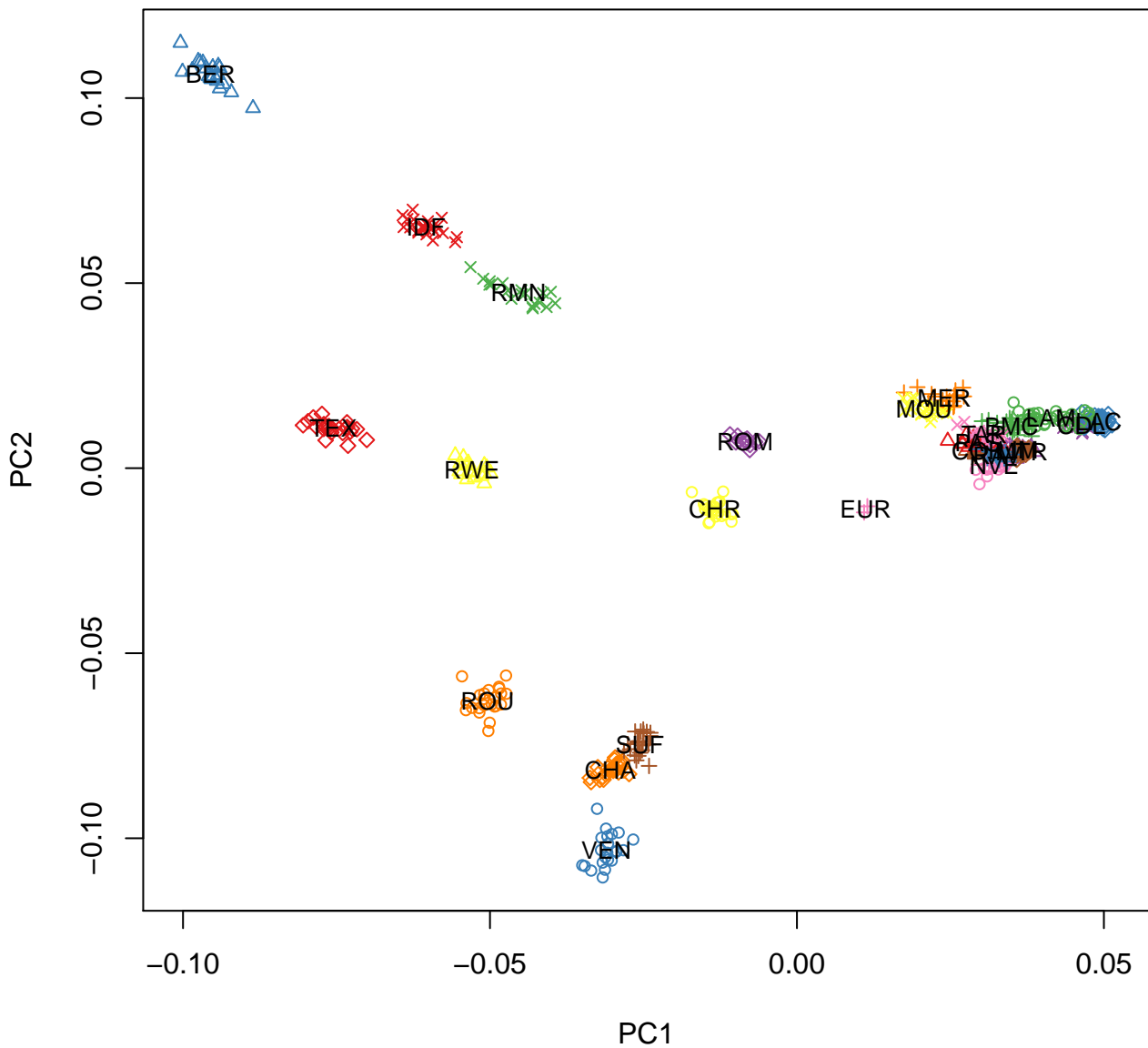

Supplement: Supplementary file 6 — Principal component analysis. PCA using 500 K SNP genotypes of all French breeds in this study except Mérinos de Rambouillet and Ouessant sheep. (PDF 19 kb) [file 12864_2018_4447_MOESM6_ESM.pdf]

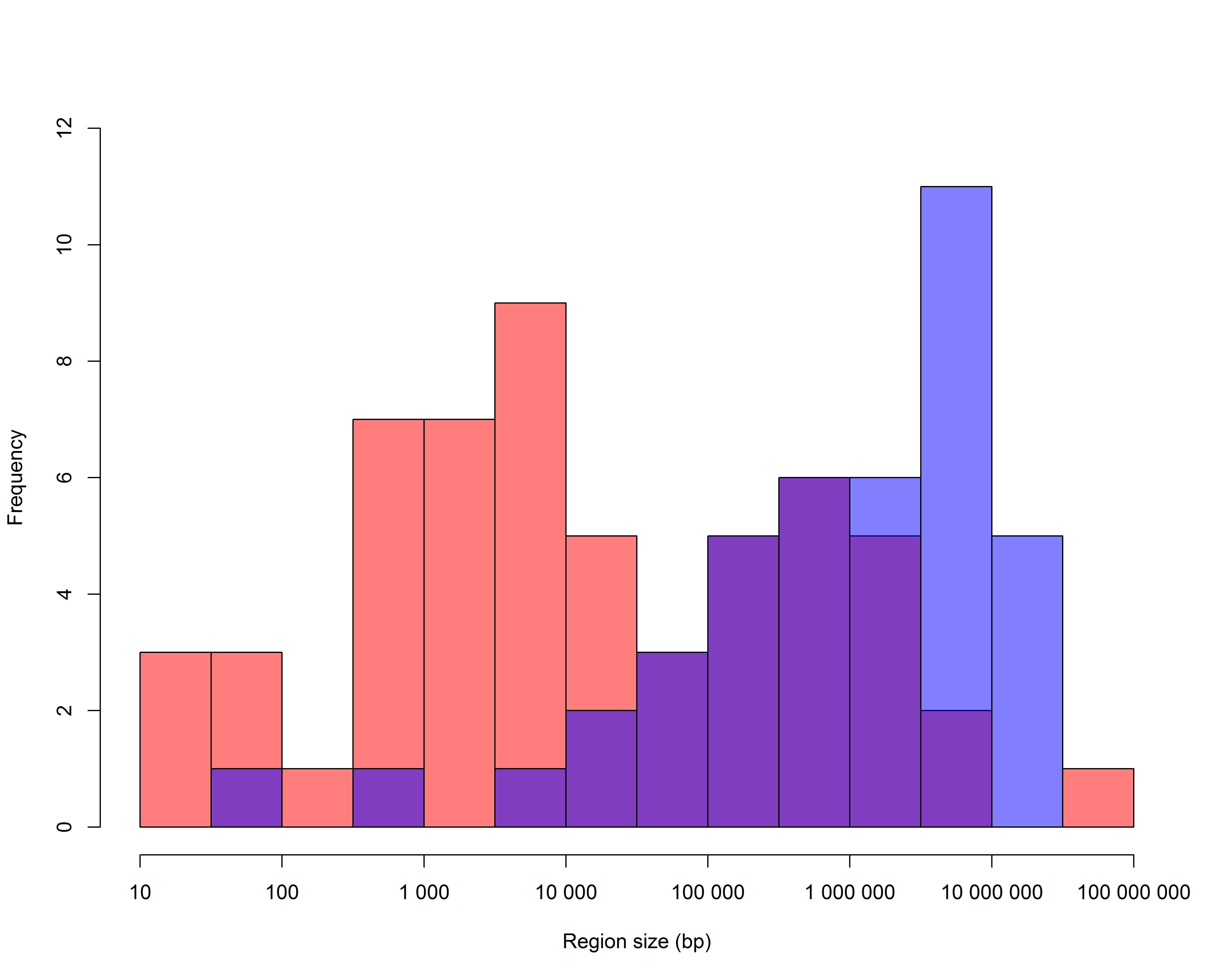

Supplement: Supplementary file 11 — Size of signatures of selection detected. Regions detected using 50 K SNPs in blue and regions detected using 500 K SNPs in pink. (PNG 123 kb) [file 12864_2018_4447_MOESM11_ESM.png]

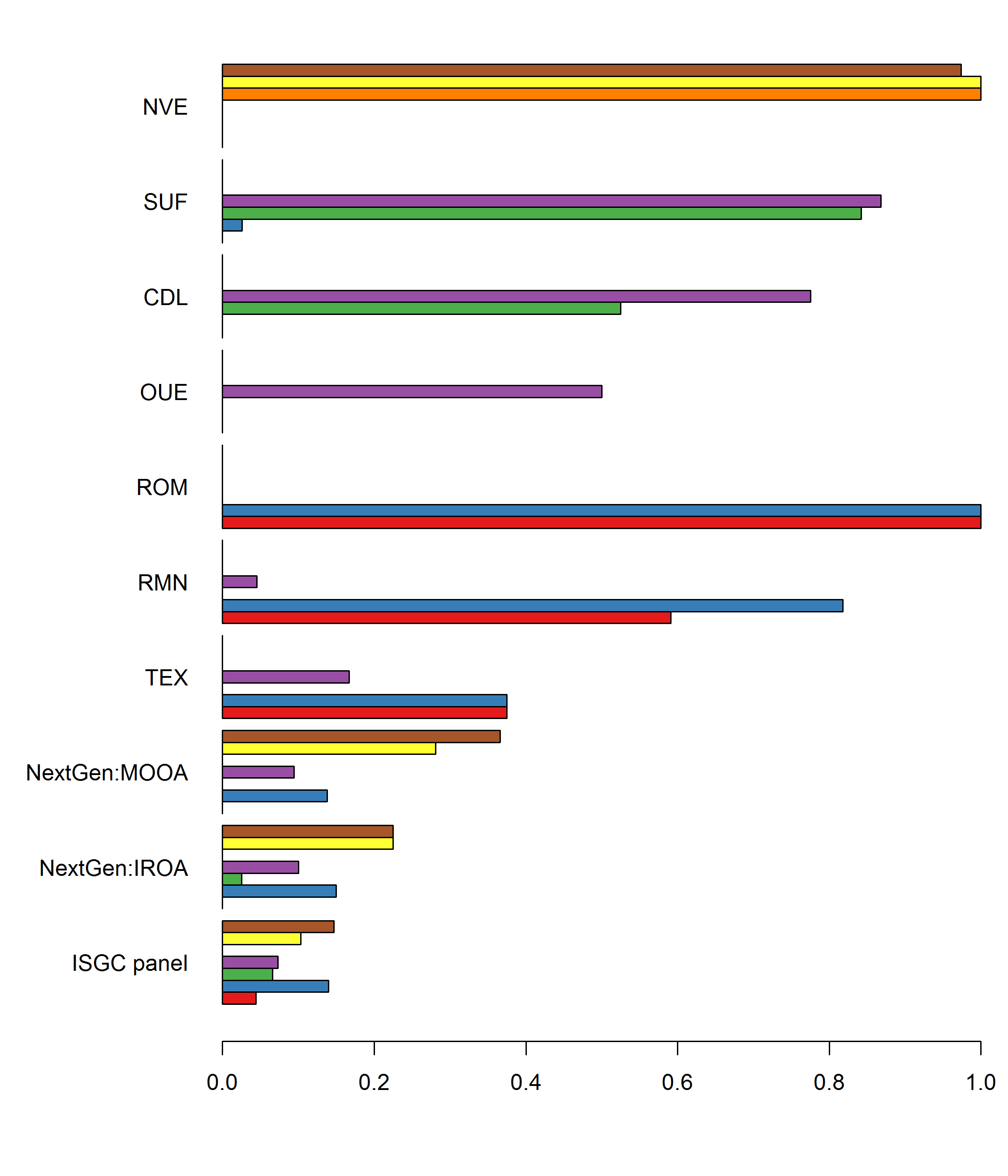

Supplement: Supplementary file 13 — Frequency of mutations in MC1R by breed. MC1R mutation frequency in Noire du Velay, Romanov, Suffolk and Texel sheep and mutation location. (PNG 40 kb) [file 12864_2018_4447_MOESM13_ESM.png]
