# Supplementary material for: Revealing the selection history of adaptive loci using genome-wide scans for selection: an example from domestic sheep
Source: BMC Genomics. 2018 Jan 23;19:71. doi: 10.1186/s12864-018-4447-x (PMC5778797; doi:10.1186/s12864-018-4447-x)
Supplement: Supplementary file 5 — PCR and sequencing primers for sequencing MC1R gene. (PDF 185 kb) [file 12864_2018_4447_MOESM5_ESM.pdf]

Primer	Sequence (5’3’)	Strand	Location on OAR14 v3.1	Comments
MC1R_R2	CAGGACACCAGCCTCCAG	minus	14231888-14231905	MC1R PCR amplification
MC1R_F2	AGCCATGAGTTGAGCAGGAC	plus	14231640-14231659	MC1R PCR amplification and sequencing
MC1R_R3	ACATAGAGGACGGCCATCAG	minus	14232191-14232210	MC1R PCR amplification and sequencing
MC1R_F3	GTGAGCGTCAGCAACGTG	plus	14231846-14231863	MC1R PCR amplification and sequencing
MC1R_R4	TGGTCTAGCGATCCTCTTTG	minus	14232595-14232614	MC1R PCR amplification
MC1R_F4	GCCTGGTTGGCTTCTTCATA	plus	14232159-14232178	MC1R PCR amplification and sequencing
MC1R_tot_R	CCTCTTTGTCAAGGGACTGC	minus	14232583-14232602	MC1R PCR amplification and sequencing
MC1R_tot_F	CTGAGAGCAAGCACCCTTTC	plus	14231661-14231680	MC1R PCR amplification and sequencing
MC1R_tot_R2	GGGCCGACATTTGTCCAG	plus	14231587-14231604	MC1R PCR amplification and sequencing
MC1R_regTot_F	GGAGTGAACGAGGCAGAGAC	plus	14228283-14228302	MC1R long range PCR amplification and sequencing
MC1R_regTot_R	TCTCCTGGGTTTCGTAGTGG	minus	14235487-14235506	MC1R long range PCR amplification and sequencing
MC1R_reg5_1_F	GTCTGTTGGGGATCTTCTGG	plus	14228907-14228888	MC1R sequencing
MC1R_reg5_2_F	ACATGGTCTCACGTGGTGTG	plus	14229619-14229638	MC1R sequencing
MC1R_reg5_3_F	CAGGAACAGGCAAACAGTCC	plus	14230160-14230141	MC1R PCR amplification
MC1R_reg5_4_F	TAGGAGGGTTTGTCGTCAGC	plus	14230754-14230773	MC1R long range PCR amplification and sequencing
MC1R_reg5_5_F	ACCTTCAGGAGGAGGGACTC	plus	14231361-14231342	MC1R long range PCR amplification and sequencing
MC1R_reg3_1_F	TGAGAGGGGAATCACAGGAC	plus	14232651-14232670	MC1R sequencing
MC1R_reg3_2_F	TTCTGTCTGTGGTGCAAACC	plus	14233259-14233278	MC1R sequencing
MC1R_reg3_3_F	AGCTGGAAACCAAACACAGG	plus	14233974-14233993	MC1R long range PCR amplification and sequencing
MC1R_reg3_4_F	CCACTGCAGAGAGGAGCAG	plus	14234589-14234607	MC1R sequencing
MC1R_reg5Tot_R	CTCAGGGTCCTGCTCAACTC	minus	14231646-14231665	MC1R PCR amplification
MC1R_reg5Tot_F	ACAGGTGTGACCCAGTCCTC	plus	14228594-14228613	MC1R long range PCR amplification and sequencing
MC1R_reg3_3_R	CTTTGTCAGAAGGGGCAGAC	minus	14234781-14234800	MC1R PCR amplification and sequencing
MC1R_reg5_3_R	ACCTCCCTTCCCTGTCTAGG	minus	14230588-14230607	MC1R long range PCR amplification and sequencing
MC1R_reg3_2_R	TCAGCCACAGCGTGTATAGC	minus	14234127-14234146	MC1R PCR amplification and sequencing
