# Supplementary material for: Revealing the selection history of adaptive loci using genome-wide scans for selection: an example from domestic sheep
Source: BMC Genomics. 2018 Jan 23;19:71. doi: 10.1186/s12864-018-4447-x (PMC5778797; doi:10.1186/s12864-018-4447-x)
Supplement: Supplementary file 7 — F3 test results for migration events detected in the population tree estimated using treemix. (PDF 111 kb) [file 12864_2018_4447_MOESM7_ESM.pdf]

Populations	f3_statistic	f3_SE	z-score
BER;RAM,TEX	0.0292914	0.000607718	48.199
RAM;BER,TEX	0.0818687	0.000955433	85.6875
TEX;BER,RAM	0.0260161	0.000578346	44.9836
BER;RMN,ROM	0.0399587	0.000523378	76.3477
RMN;BER,ROM	-0.0109204	0.00020695	-52.7685
ROM;BER,RMN	0.0306937	0.000418983	73.2576
IDF;TEX,MOU	0.0166587	0.000310984	53.5675
MOU;TEX,IDF	0.0115723	0.000272959	42.3959
TEX;MOU,IDF	0.026107	0.000439397	59.4156
