# Supplementary material for: Revealing the selection history of adaptive loci using genome-wide scans for selection: an example from domestic sheep
Source: BMC Genomics. 2018 Jan 23;19:71. doi: 10.1186/s12864-018-4447-x (PMC5778797; doi:10.1186/s12864-018-4447-x)
Supplement: Supplementary file 8 — FLK results. FLK regions under selection, SNP frequencies, haplotype cluster frequencies and SNP and haplotype local trees. (PDF 14210 kb) [file 12864_2018_4447_MOESM8_ESM.pdf]

Supporting information: FLK results by region: SNP frequencies, local trees and haplotype cluster frequencies

Breeds:

Breed	Abbreviation
Berrichon du Cher	BER
Blanche du Massif Central	BMC
Causse du Lot	CDL
Charollais	CHA
Charmoise	CHR
Corse	COR
European Mouflon	EUR
Île de France	IDF
Asian Mouflon	IRN
Lacaune (dairy)	LAC
Lacaune (meat)	LAM
Limousine	LIM
Mérinos d’Arles	MER
Mourerous	MOU
Manech tête rousse	MTR
Noire du Velay	NVE
Ouessant	OUE
Préalpes du Sud	PAS
Mérinos de Rambouillet	RAM
Rava	RAV
Romane / INRA 401	RMN
Romanov	ROM
Roussin de la Hague	ROU
Rouge de l’Ouest	RWE
Suffolk	SUF
Tarasconnaise	TAR
Texel	TEX
Vendéen	VEN

Population tree estimated from all SNPs

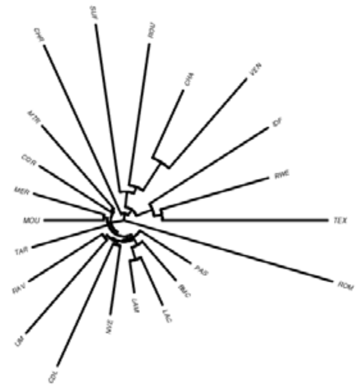

*In SNP frequency and haplotype cluster frequency plots the red dotted line is the SNP with the smallest p value and the two black lines are the beginning and end of candidate genes.

Region		
**Chr**	**Begin**	**End**
1	27383208	30143421

Best SNP: 28596396
Located in *SSBP3* [1].

SNP frequencies

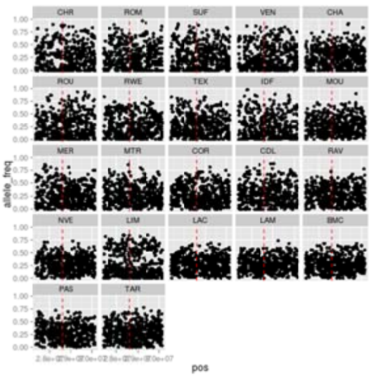

SNP local tree

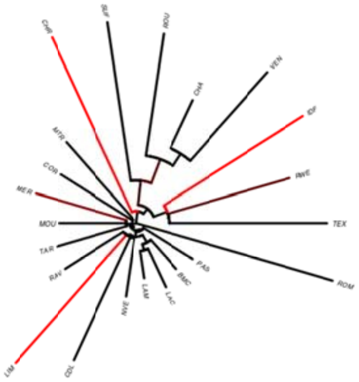

Haplotype local tree

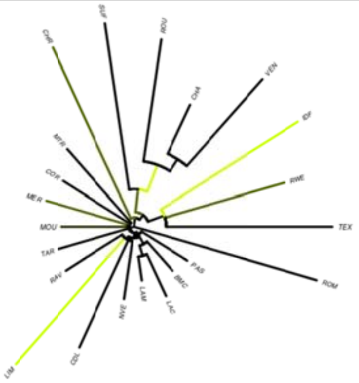

Haplotype cluster frequencies

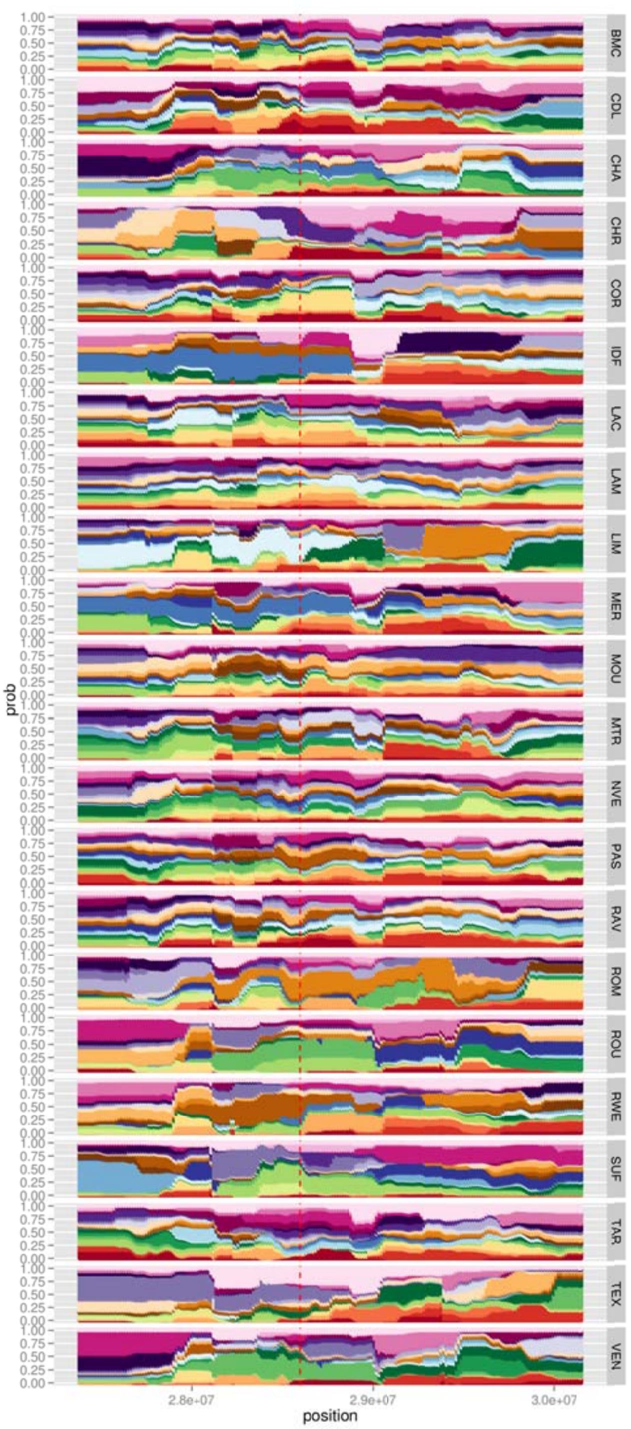

Region			SNP frequencies	Haplotype cluster frequencies
**Chr**	**Begin**	**End**		
1	38836829	39720090		

Best SNP: 39170368

Located in *ROR1* [1].

SNP frequencies

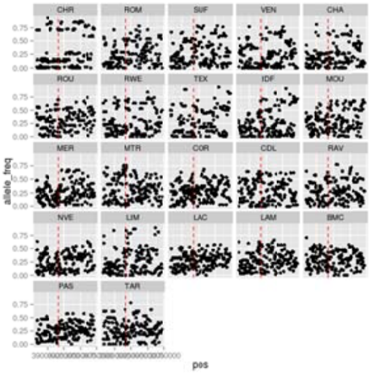

Haplotype cluster frequencies

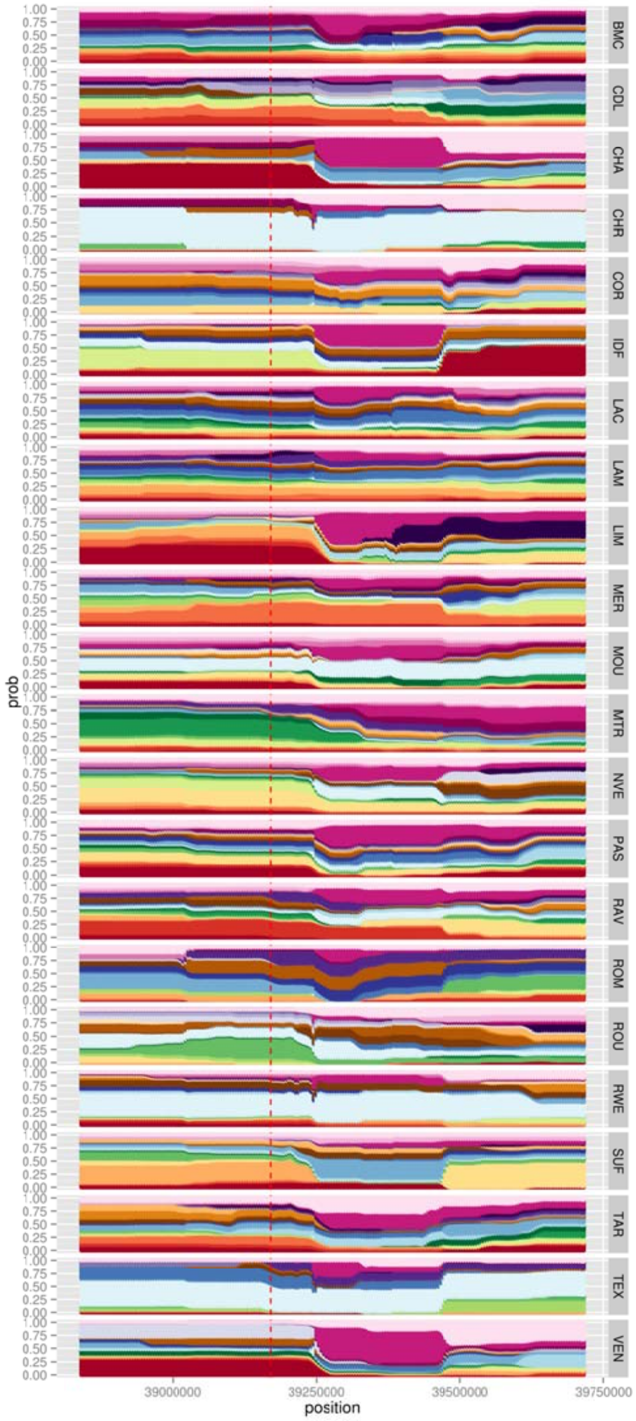

SNP local tree

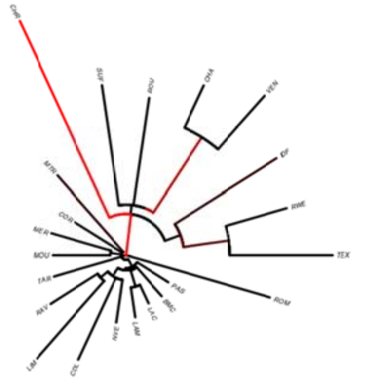

Haplotype local tree

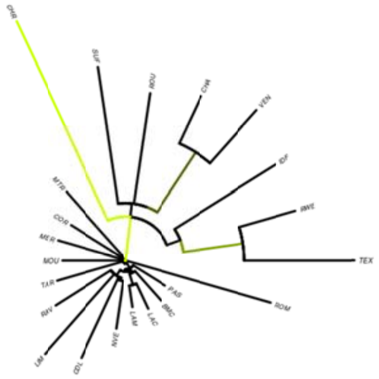

Region		
Chr	Begin	End
1	102312201	105337456

Best SNP: 103432309
Located in *ADAR1* [1].

SNP frequencies

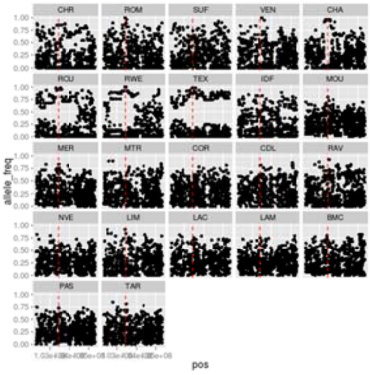

SNP local tree

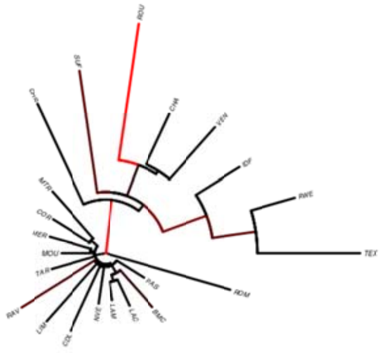

Haplotype local tree

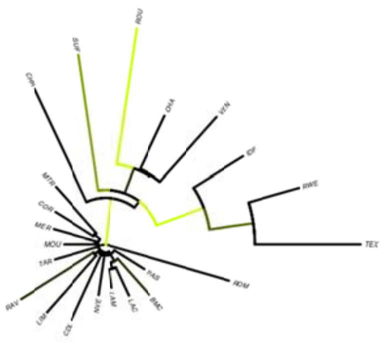

Haplotype cluster frequencies

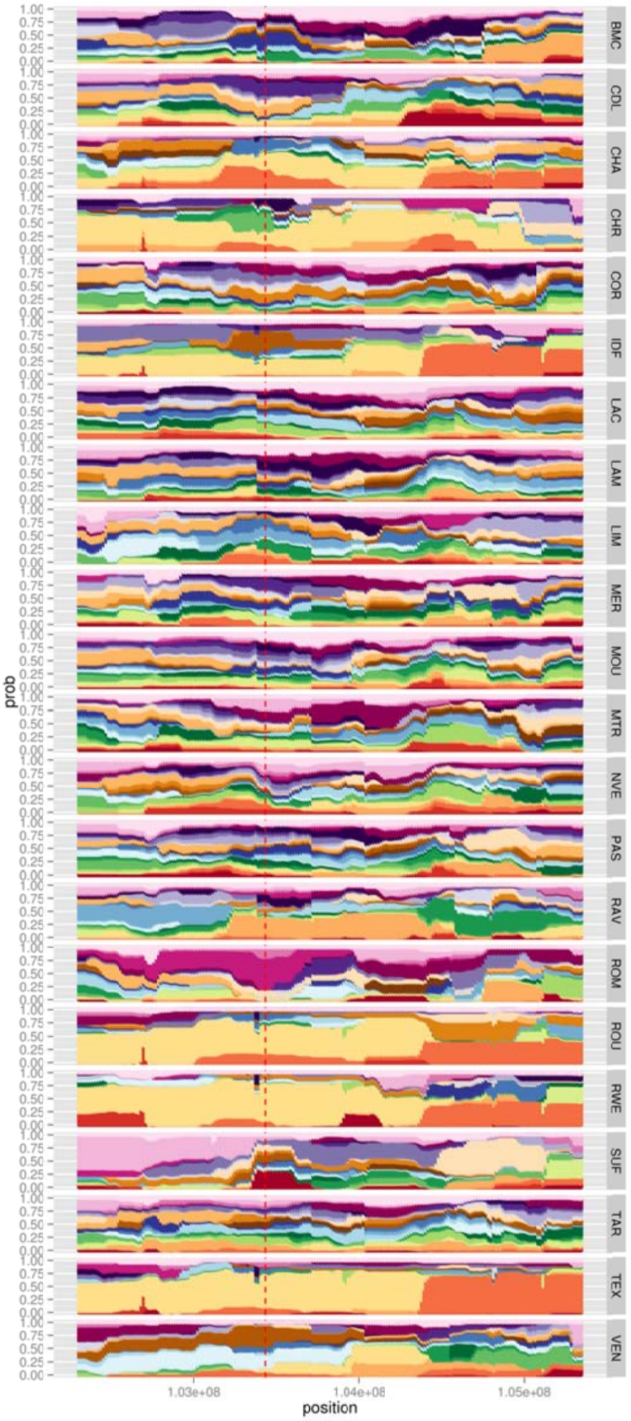

Region		
**Chr**	**Begin**	**End**
1	119252967	120575235

Best SNP: 120554658
Located in *IFNAR2* [1].

SNP frequencies

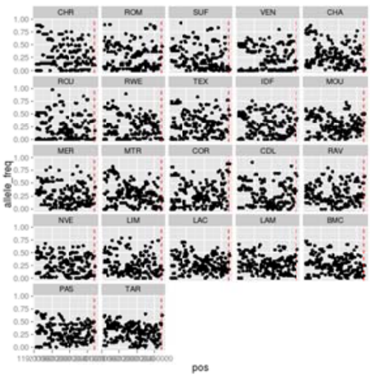

SNP local tree

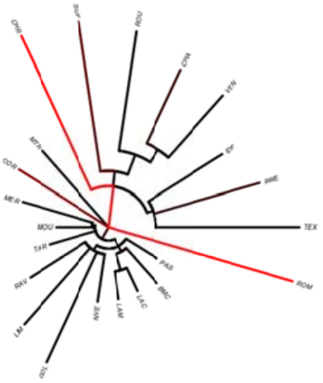

Haplotype local tree

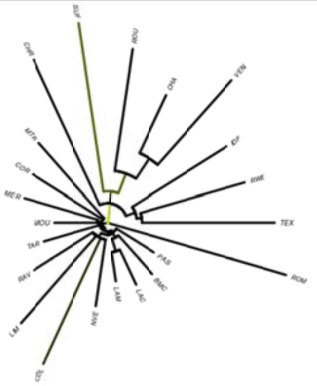

Haplotype cluster frequencies

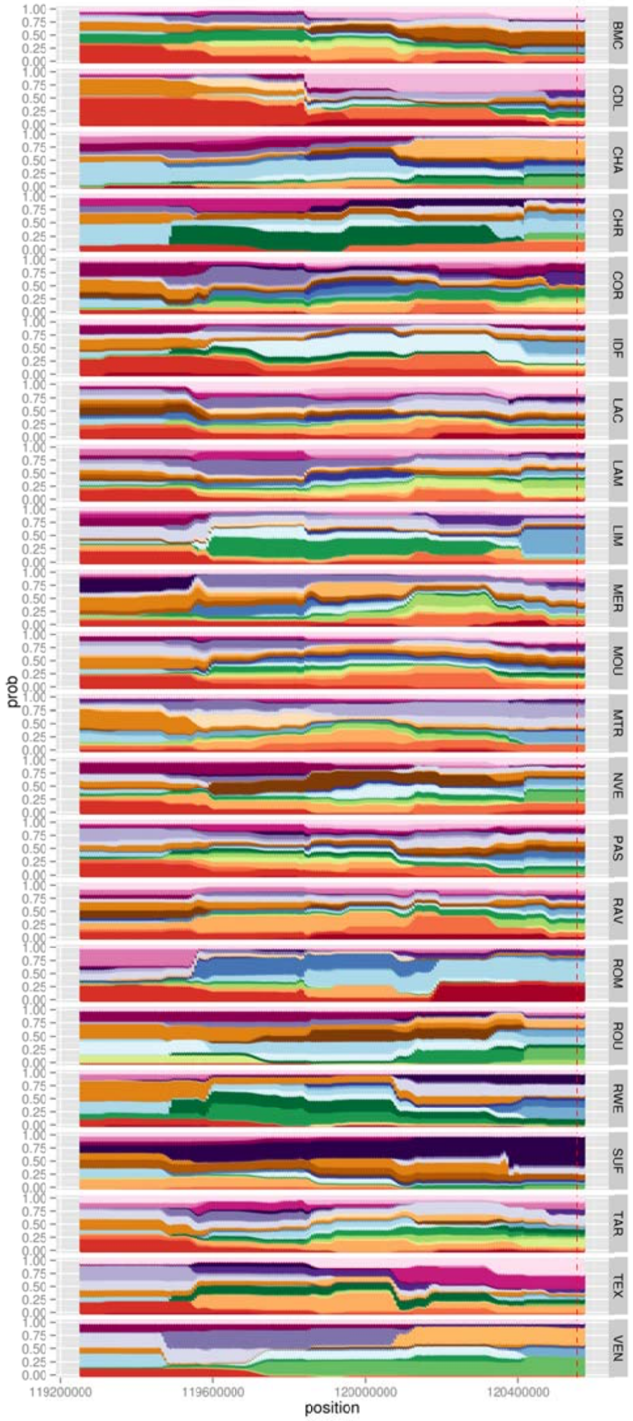

Region		
Chr	Begin	End
2	38096770	42417880

Best SNP: 39454479
Located in *EBF2* [1]
(involved in brown fat fate and function in mice [2]).

SNP frequencies

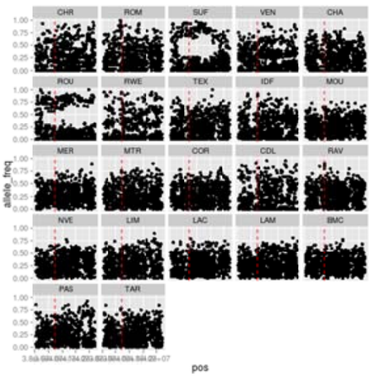

SNP local tree

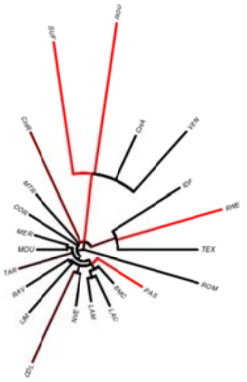

Haplotype local tree

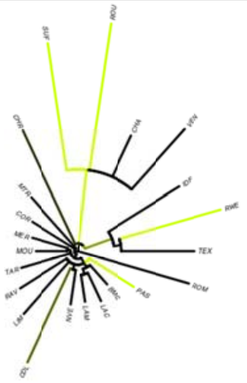

Haplotype cluster frequencies

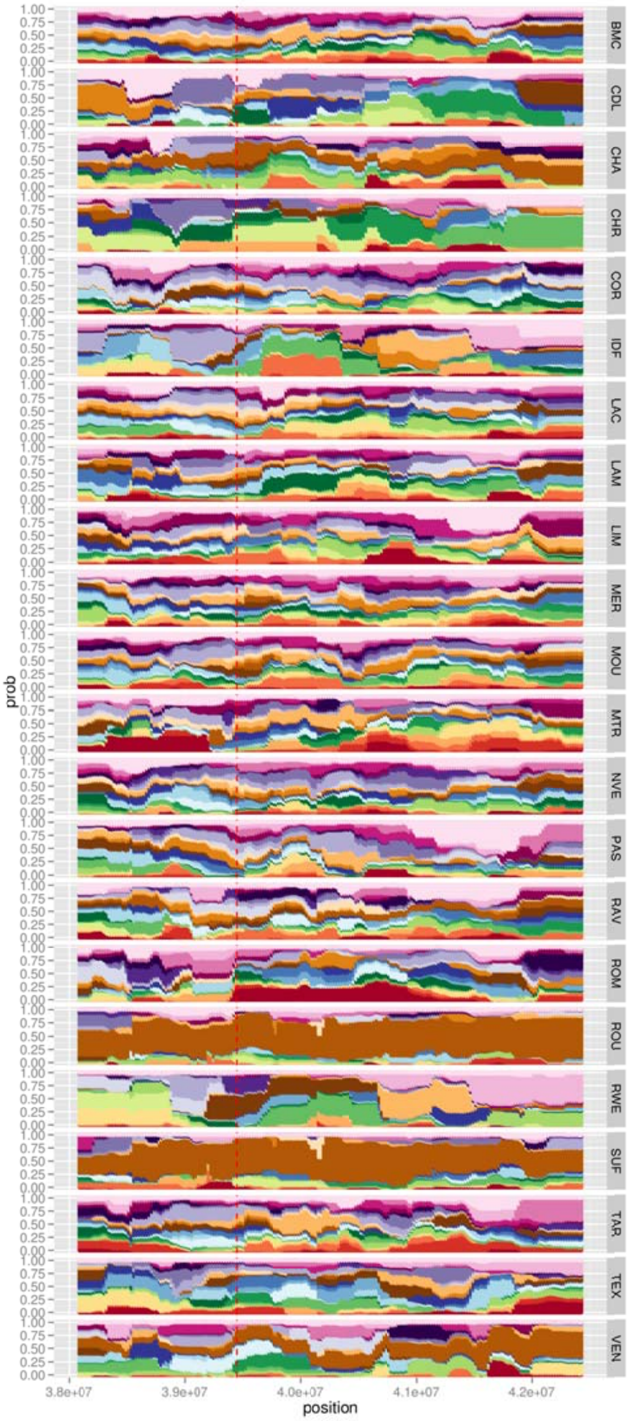

Region		
**Chr**	**Begin**	**End**
2	51788434	52911940

Best SNP: 52429848
Located in *NPR2* [1]
(mutations in *NPR2* associated with height in humans [3]).

SNP frequencies

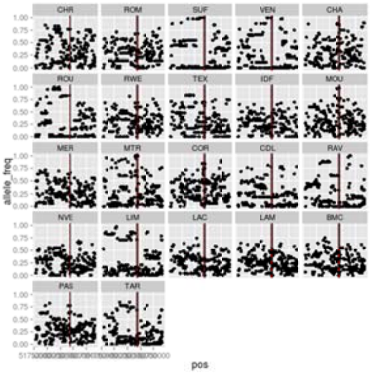

SNP local tree

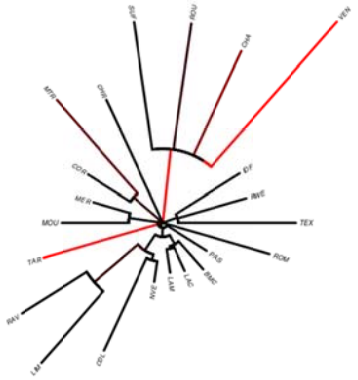

Haplotype local tree

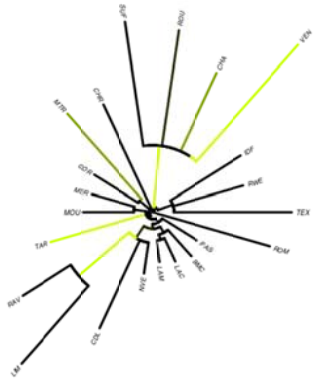

Haplotype cluster frequencies

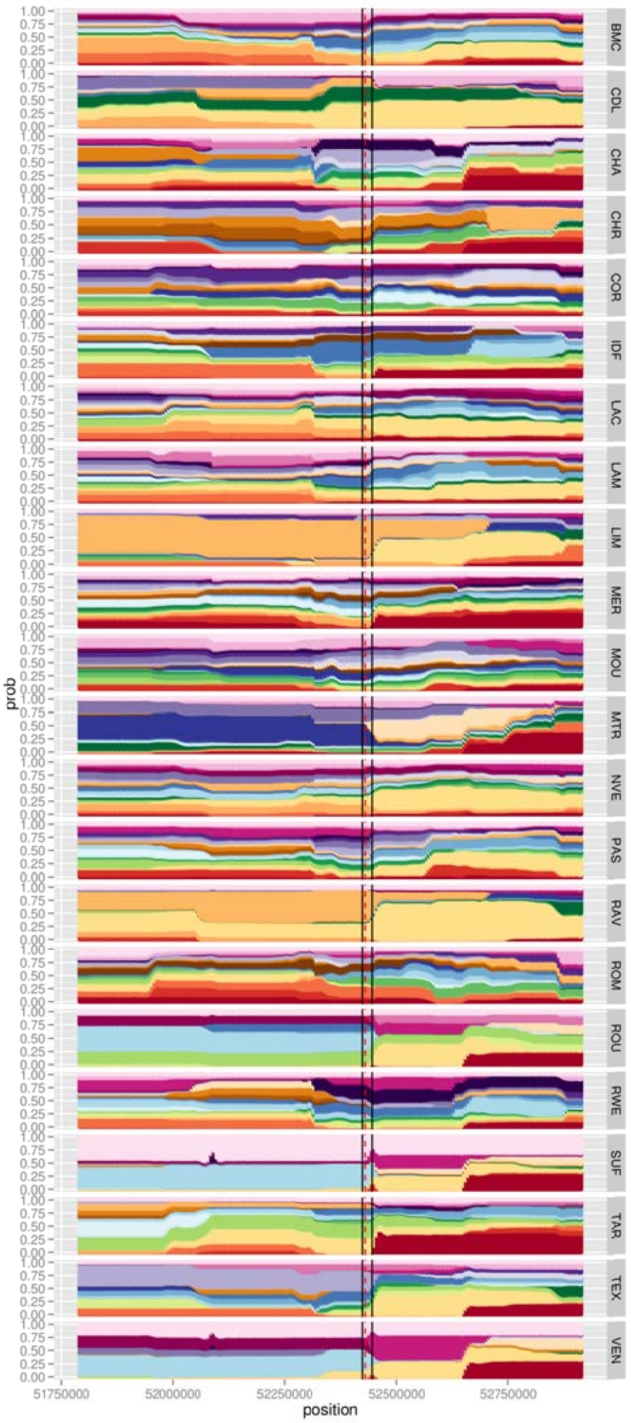

Region		
Chr	Begin	End
2	73868893	81869098

Best SNP: 78924564
Candidate gene: *TYRP1* (a mutation in *TYRP1* in Soay sheep is associated with light coat colour [4]).

SNP frequencies

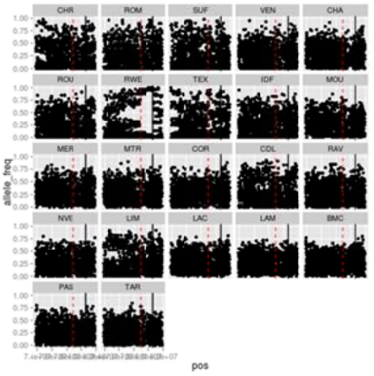

SNP local tree

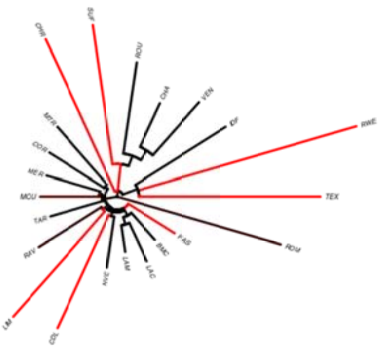

Haplotype local tree

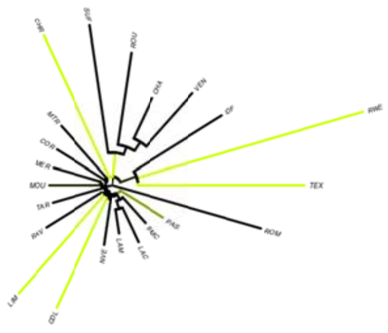

Haplotype cluster frequencies

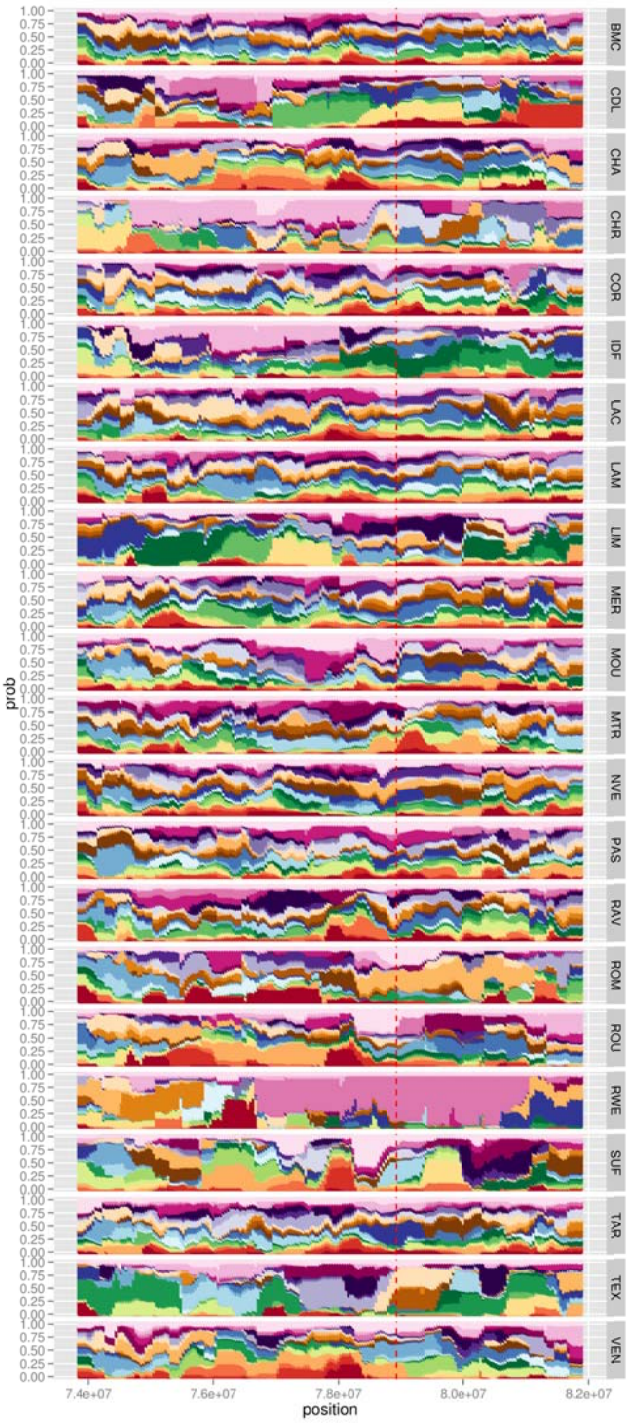

Region		
**Chr**	**Begin**	**End**
2	83470886	85199700

Best SNP: 84698214
Candidate gene: *BNC2*
(mutations in *BNC2* in humans is associated with skin colour in humans [5]).

SNP frequencies

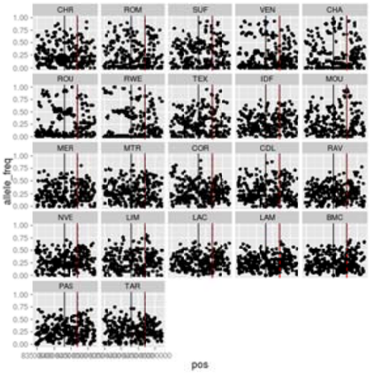

SNP local tree

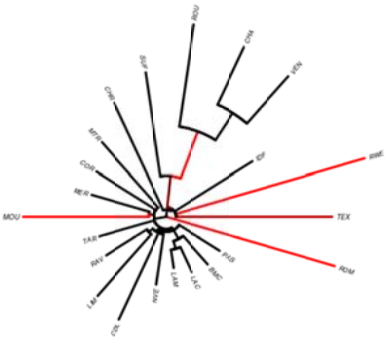

Haplotype local tree

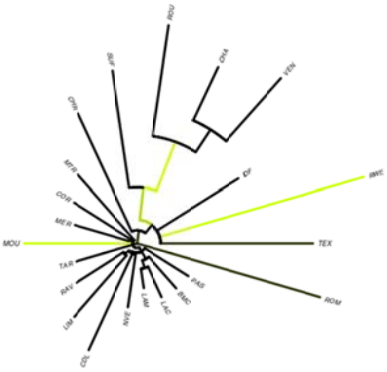

Haplotype cluster frequencies

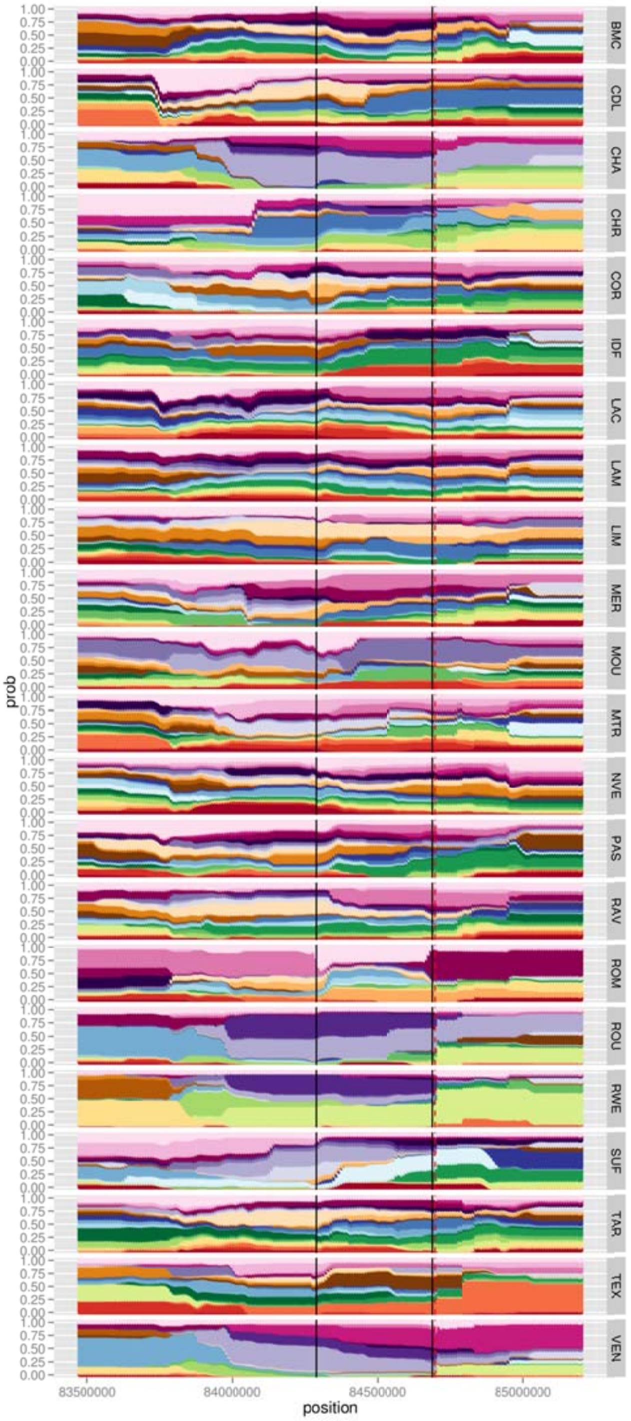

Region		
**Chr**	**Begin**	**End**
2	108979649	122258246

Best SNP: 118150665

SNP frequencies

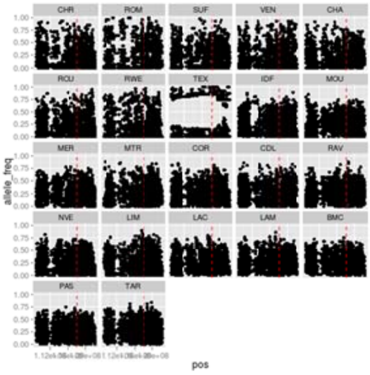

SNP local tree

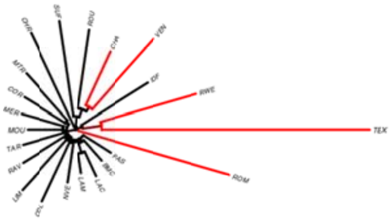

Haplotype local tree

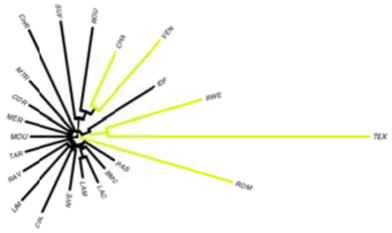

Haplotype cluster frequencies

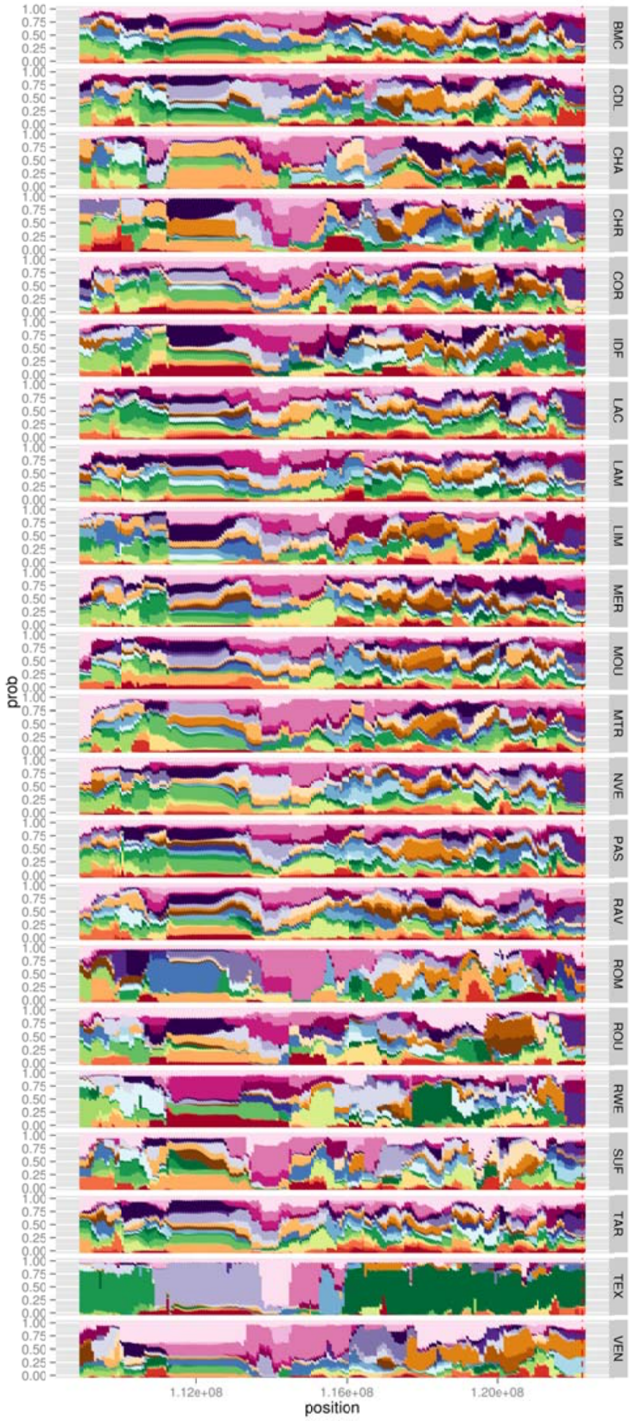

Region		
**Chr**	**Begin**	**End**
2	133339051	134985148

Best SNP: 133339051

SNP frequencies

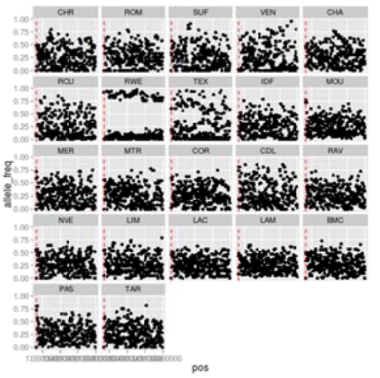

SNP local tree

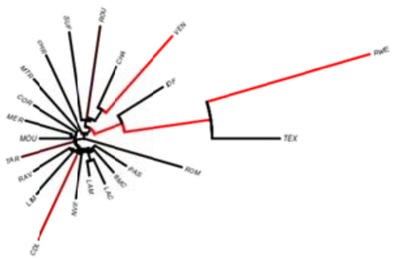

Haplotype local tree

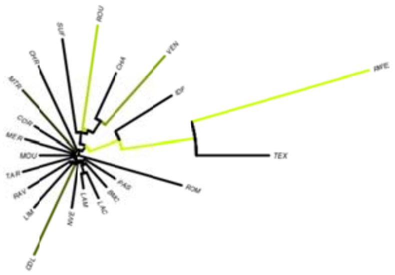

Haplotype cluster frequencies

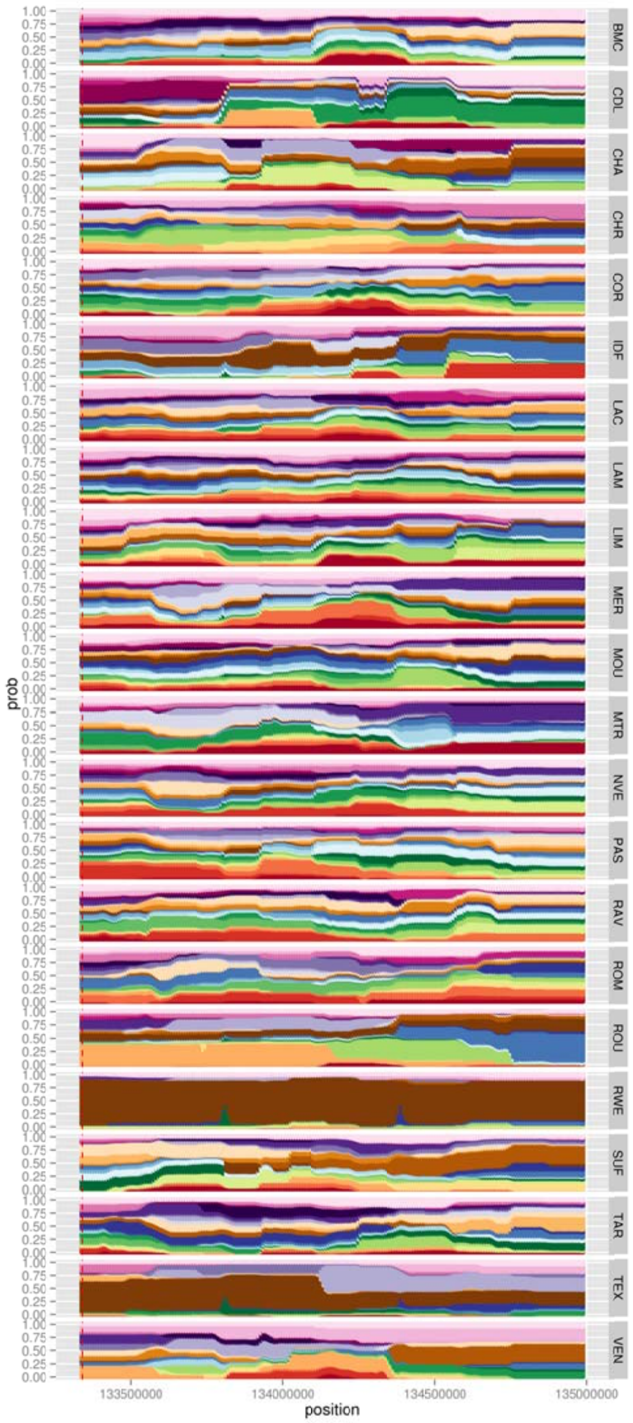

Region		
**Chr**	**Begin**	**End**
3	61876792	62174495

Best SNP: 62099364

SNP frequencies

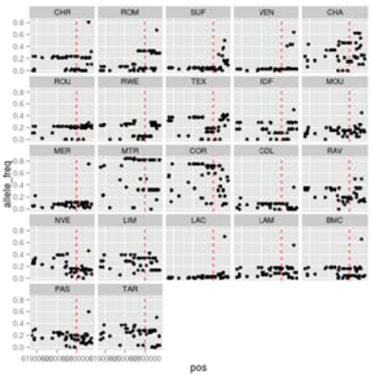

SNP local tree

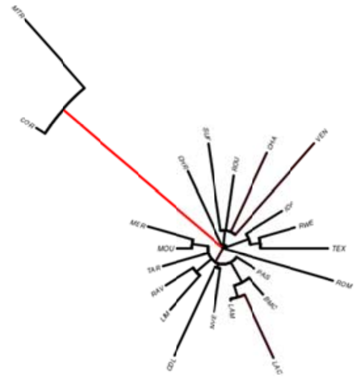

Haplotype local tree

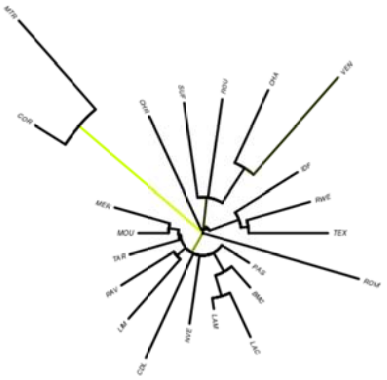

Haplotype cluster frequencies

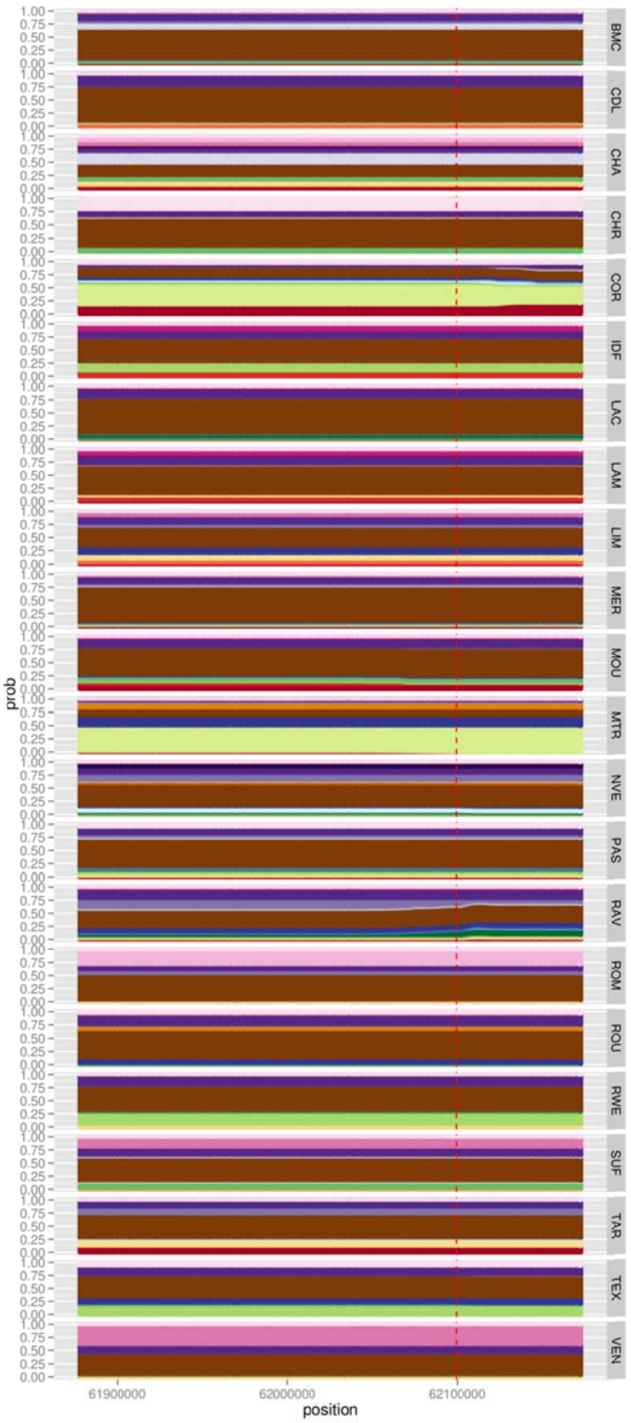

Region		
Chr	Begin	End
3	129042736	130667605

Best SNP: 129722385
Located in *SOCS2* [1] (plays roles in metabolism, somatic growth, bone formation, the central nervous system, response to infections and mammary gland development although the main target of *SOCS2* is believed to be *GH/IGF-1* which is important for somatic growth [6] and has been associated with growth and mastitis in Lacaune sheep [7]).

SNP frequencies

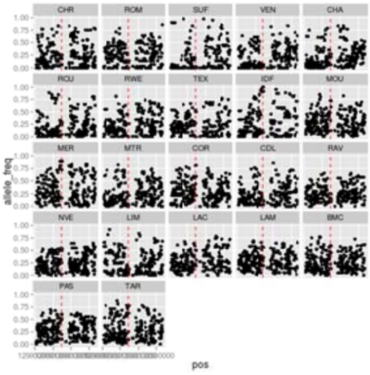

SNP local tree

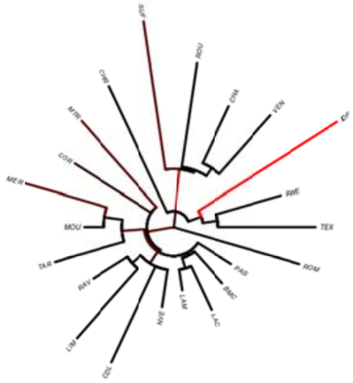

Haplotype local tree

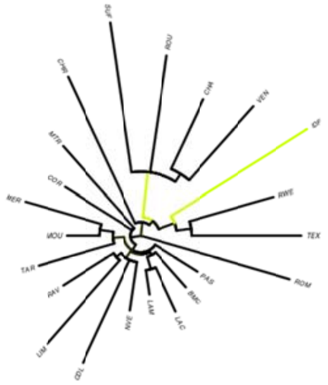

Haplotype cluster frequencies

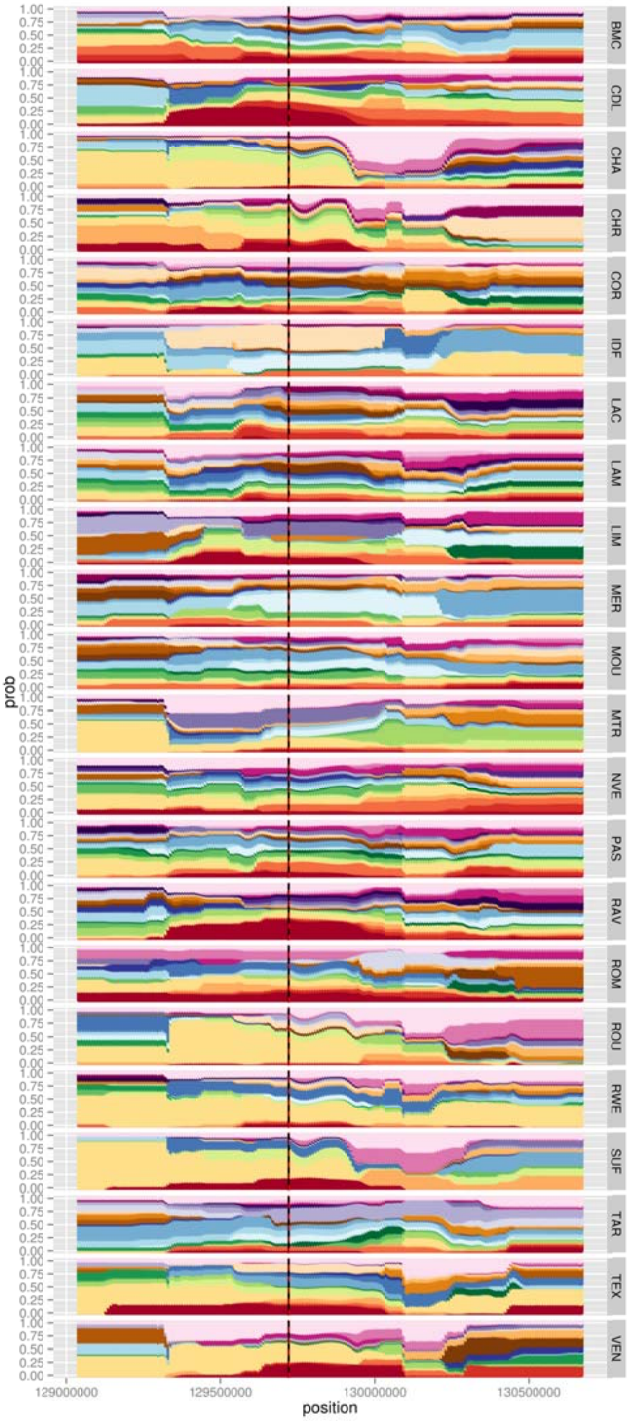

Region		
**Chr**	**Begin**	**End**
3	153596226	154607238

Best SNP: 154214608
Candidate gene: *MSRB3* (associated with ear position in dogs [8] and ear size in pigs [9]).

SNP frequencies

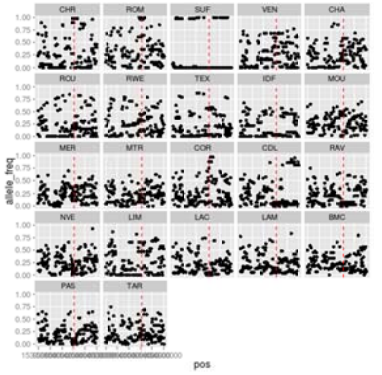

SNP local tree

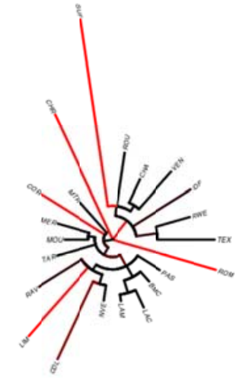

Haplotype local tree

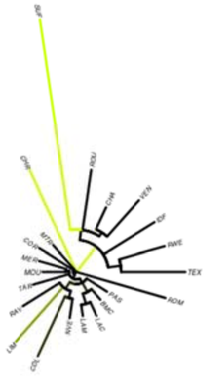

Haplotype cluster frequencies

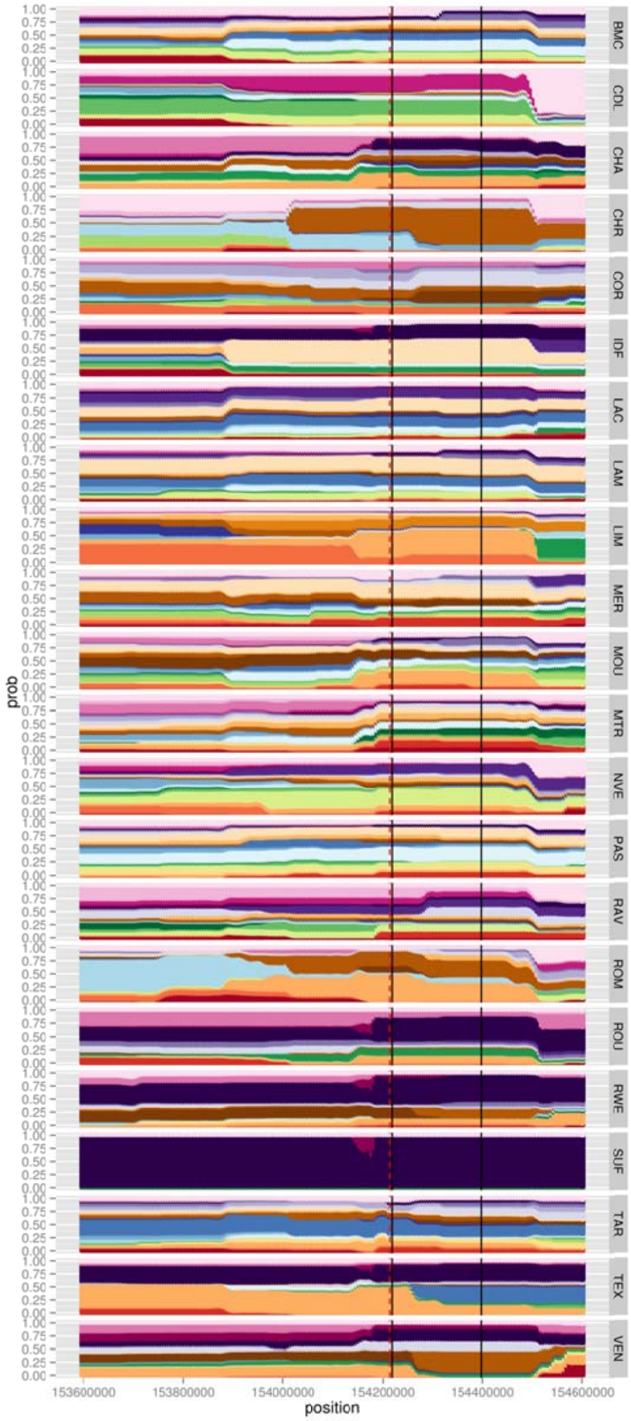

Region		
Chr	Begin	End
3	209486703	211328458

Best SNP: 210938692
Located in *FKBP4* [1].

SNP frequencies

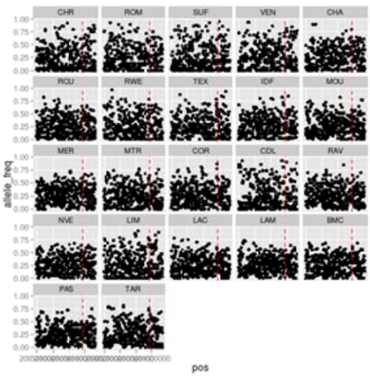

SNP local tree

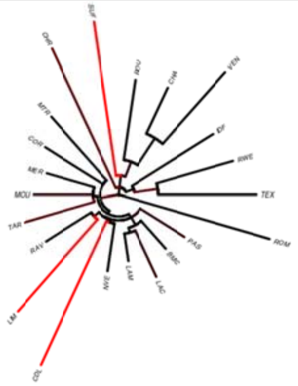

Haplotype local tree

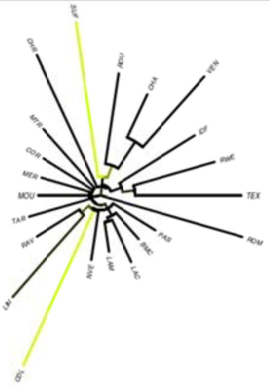

Haplotype cluster frequencies

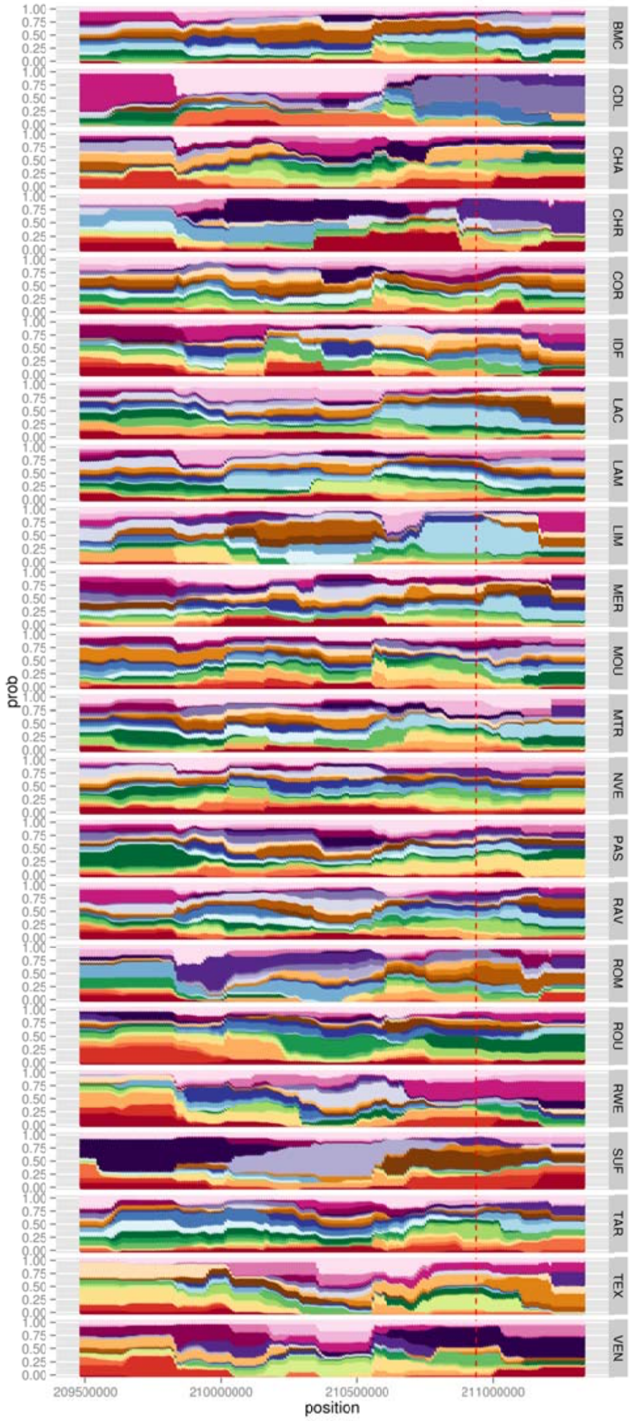

Region		
**Chr**	**Begin**	**End**
5	51641560	57831147

Best SNP: 56909778

SNP frequencies

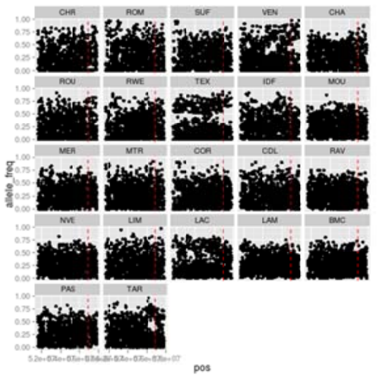

SNP local tree

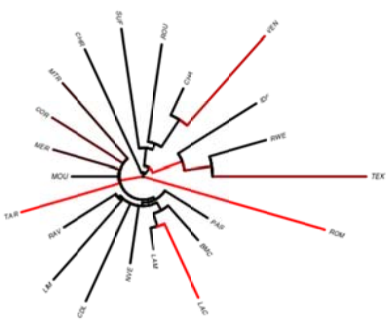

Haplotype local tree

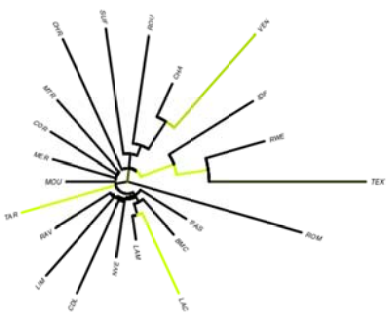

Haplotype cluster frequencies

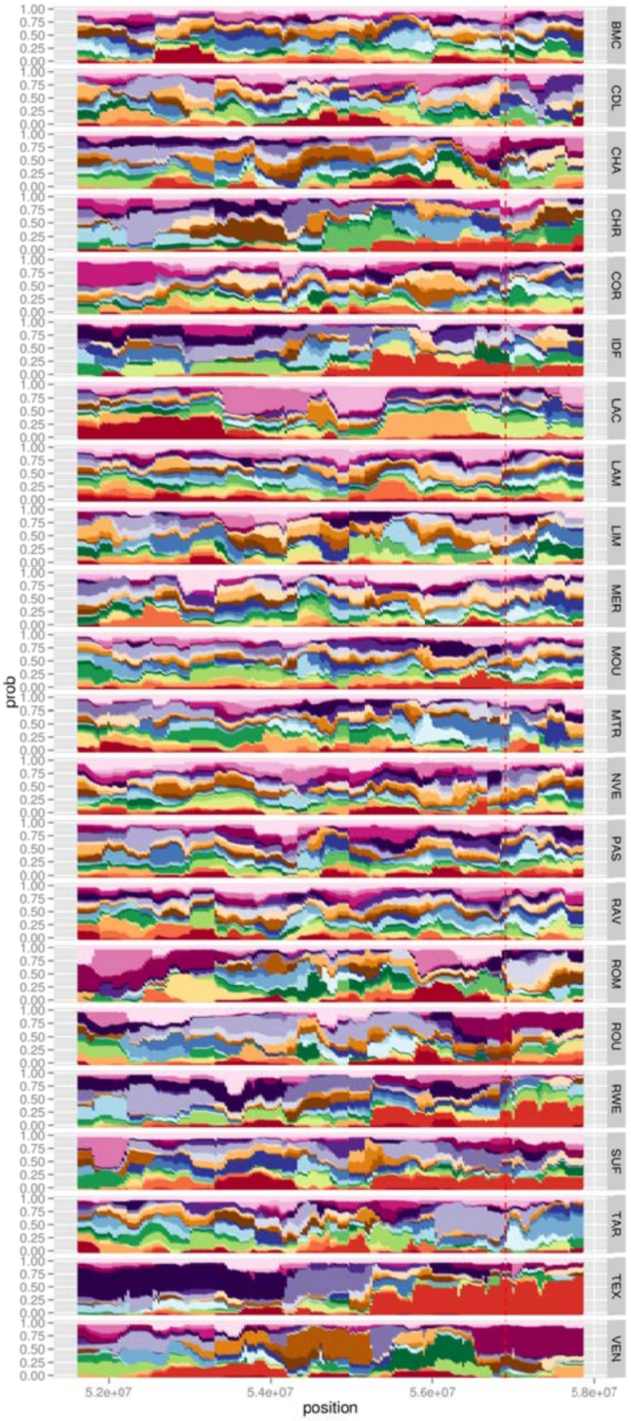

Region			SNP frequencies	Haplotype cluster frequencies
**Chr**	**Begin**	**End**		
6	26112692	47828792		

Best SNP: 35762202
Located in *FAM13A* [1].
Candidate genes: *ABCG2* (associated with milk production in sheep [10]) and *NCAPG2*/*LCORL* (associated with height in cattle [11]) outlined in black.

SNP frequencies

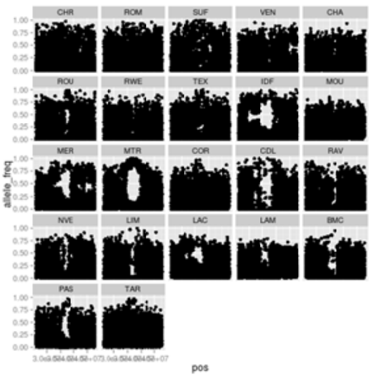

SNP local tree

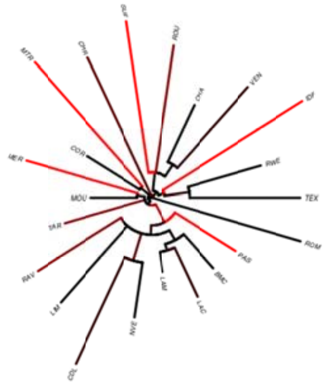

Haplotype local tree

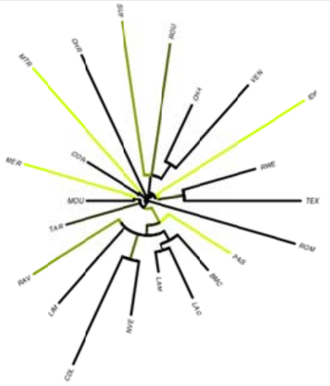

Haplotype cluster frequencies

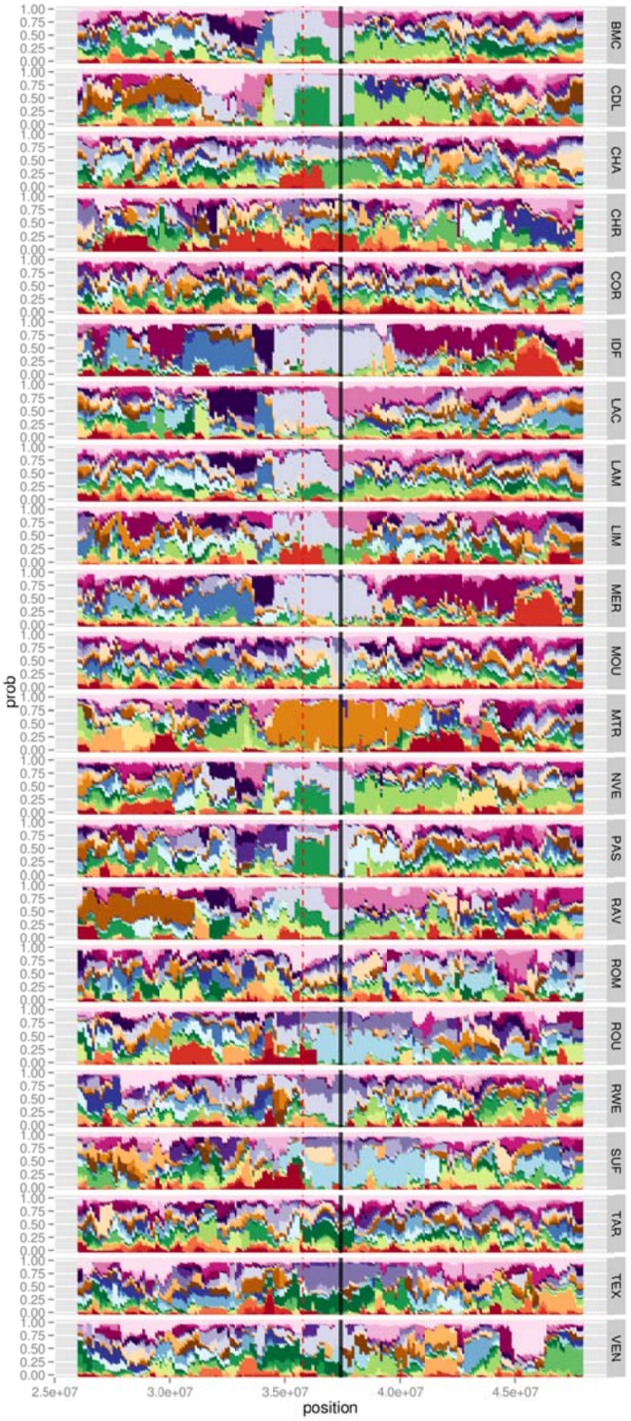

Region		
**Chr**	**Begin**	**End**
6	57693565	59301412

Best SNP: 59252594

SNP frequencies

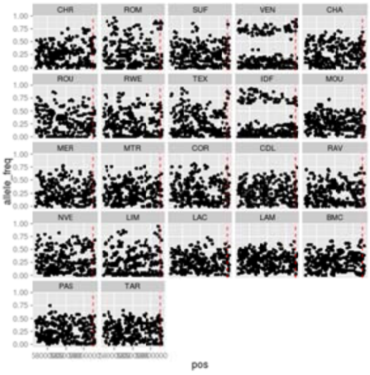

SNP local tree

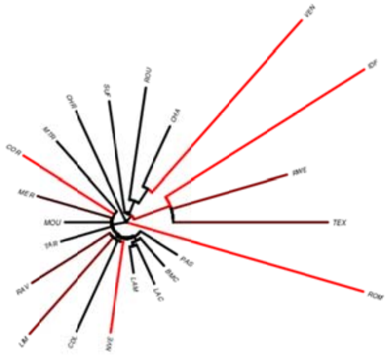

Haplotype local tree

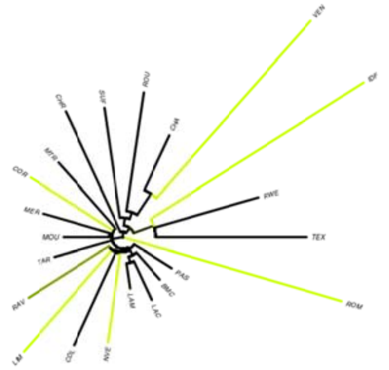

Haplotype cluster frequencies

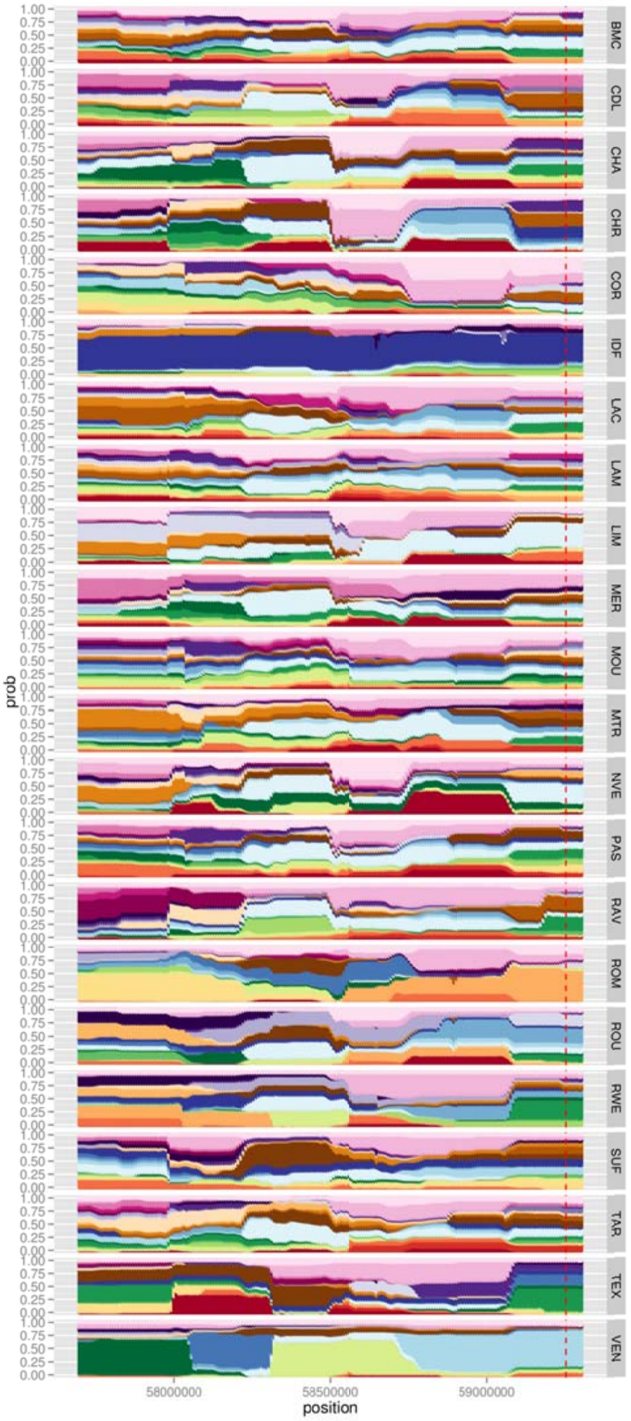

Region		
**Chr**	**Begin**	**End**
6	94242119	96008214

Best SNP: 95193314
Located in *C4orf22* [1].

SNP frequencies

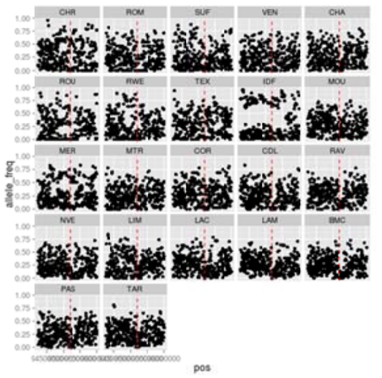

SNP local tree

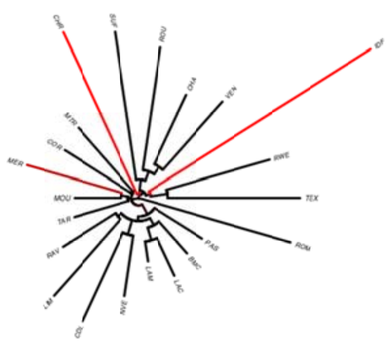

Haplotype local tree

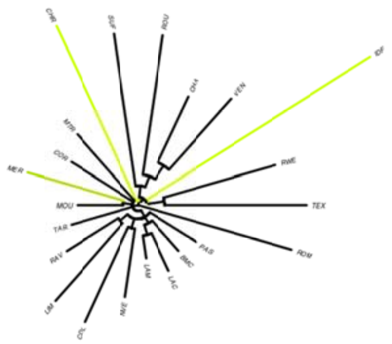

Haplotype cluster frequencies

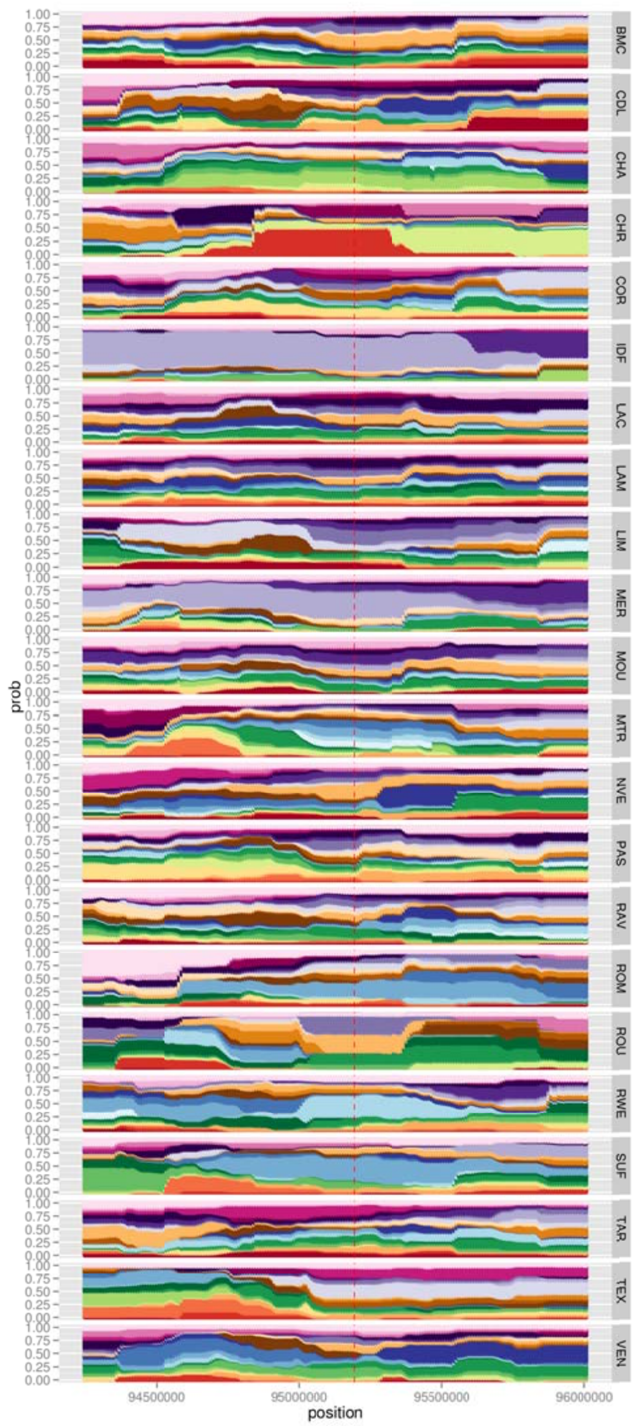

Region		
**Chr**	**Begin**	**End**
8	30158735	32611917

Best SNP: 31402984

SNP frequencies

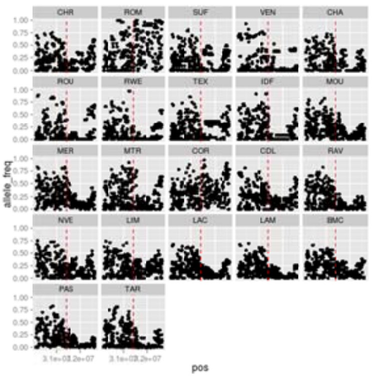

SNP local tree

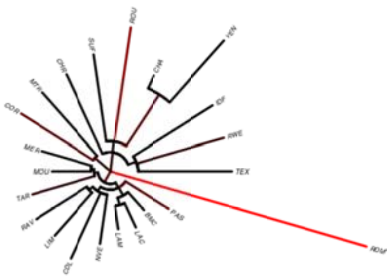

Haplotype local tree

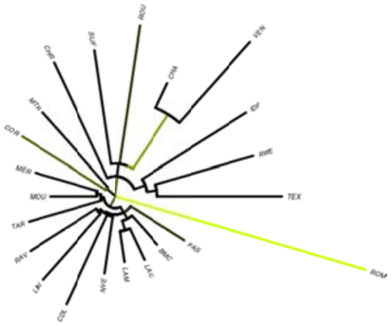

Haplotype cluster frequencies

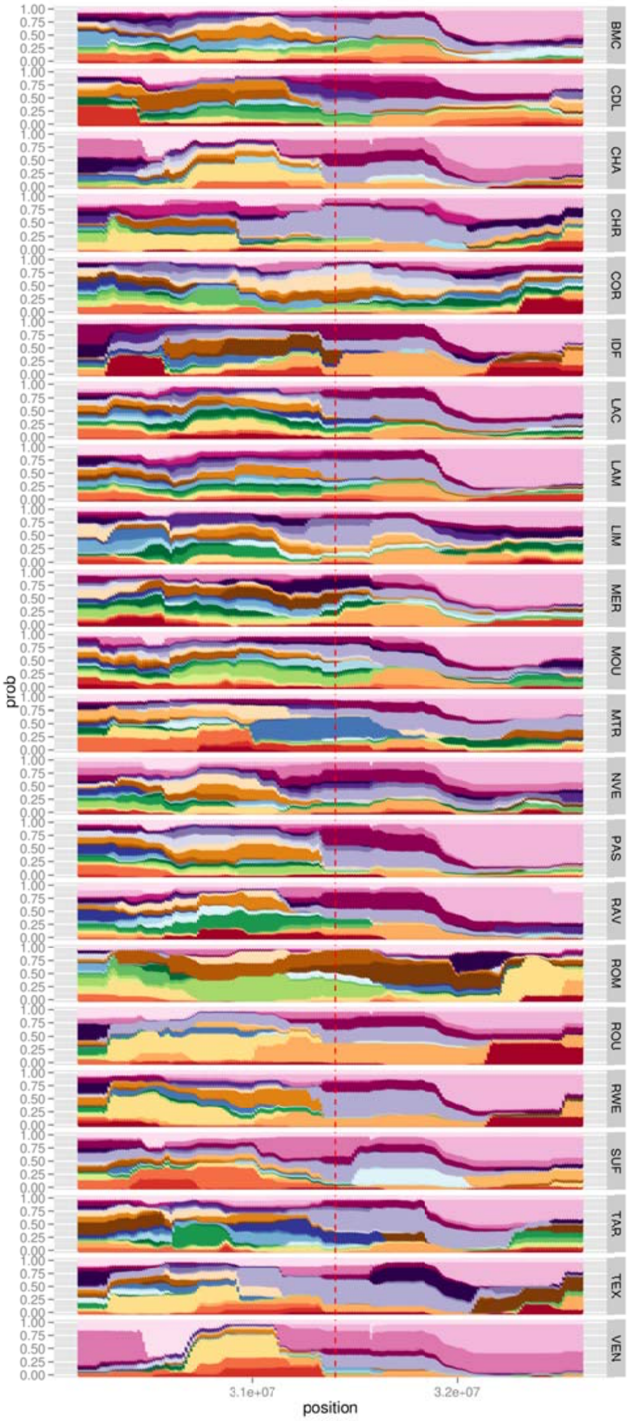

Region		
Chr	Begin	End
8	80676681	83105269

Best SNP: 80707878
Located in *zdhhc14* [1].

SNP frequencies

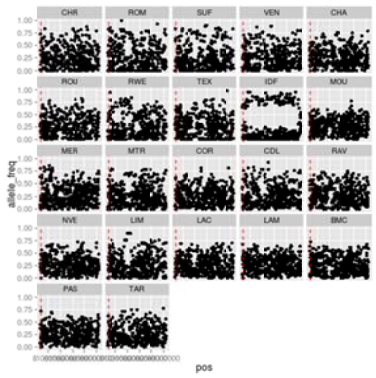

SNP local tree

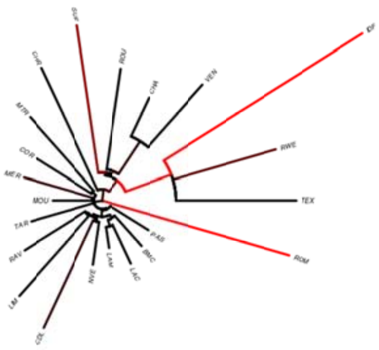

Haplotype local tree

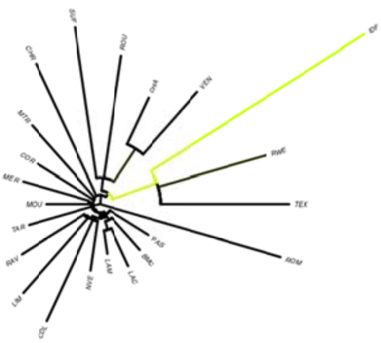

Haplotype cluster frequencies

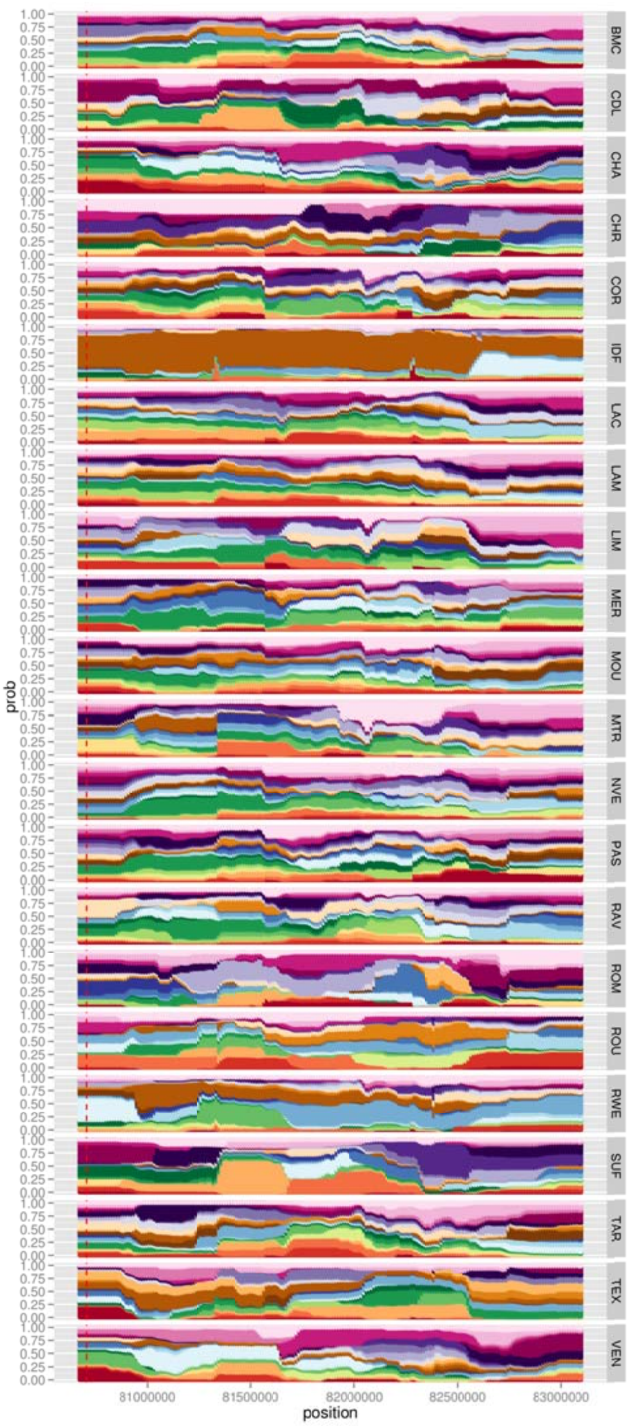

Region		
**Chr**	**Begin**	**End**
10	26935215	31039477

Best SNP: 29379118
Located in *Elongation factor 1-alpha* [1].
Candidate gene: *RXFP2* (associated with horn development in sheep [12]).

SNP frequencies

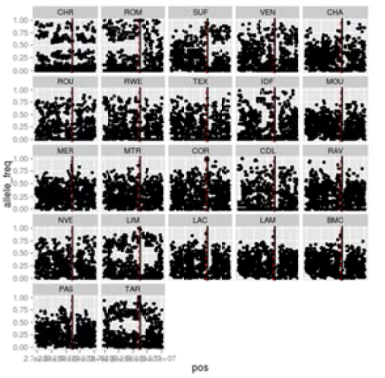

SNP local tree

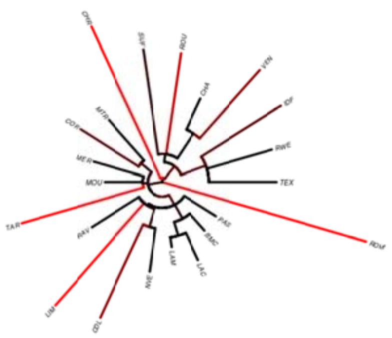

Haplotype local tree

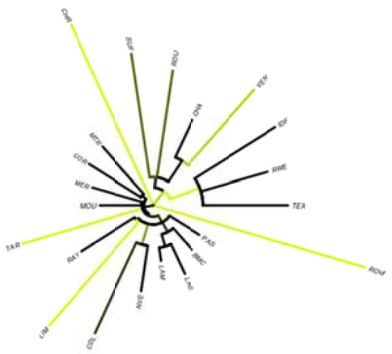

Haplotype cluster frequencies

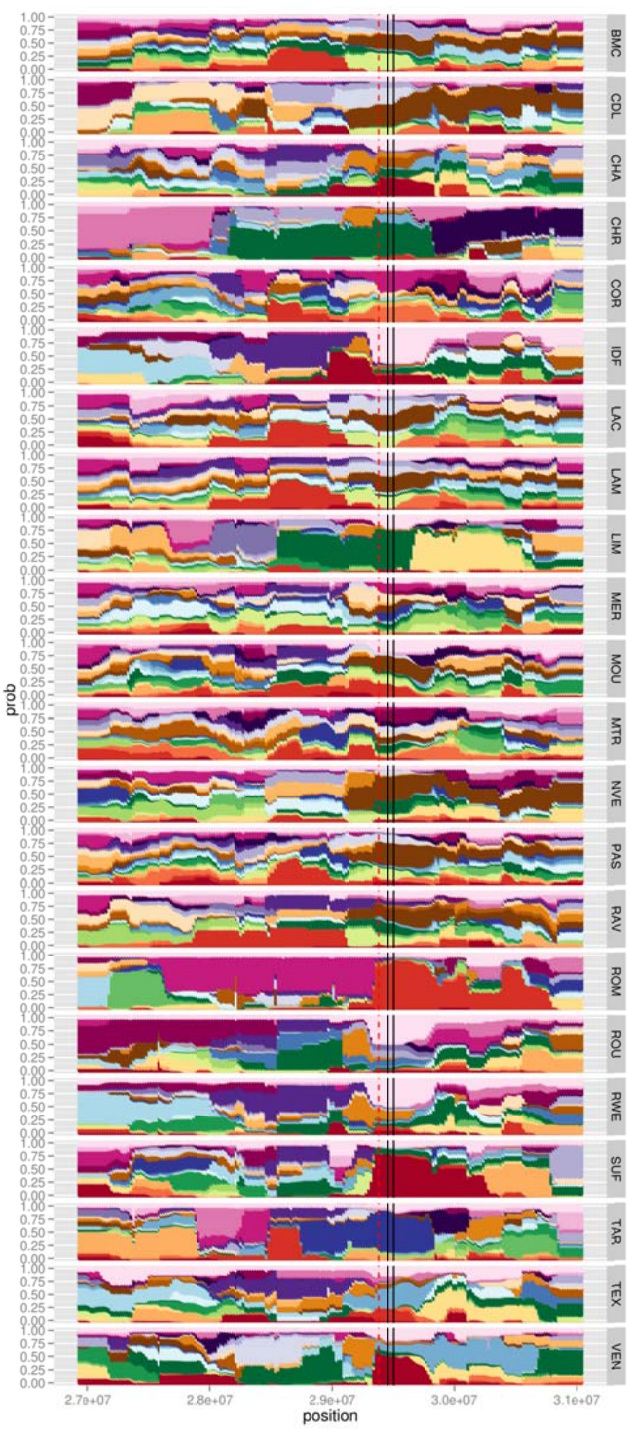

Region		
**Chr**	**Begin**	**End**
10	52563951	53714120

Best SNP: 53602818

SNP frequencies

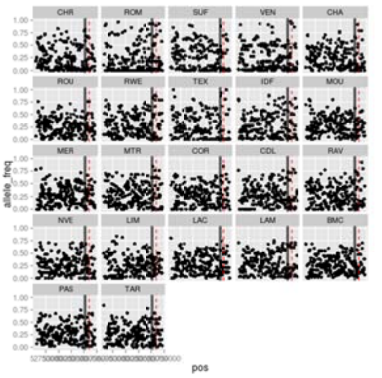

SNP local tree

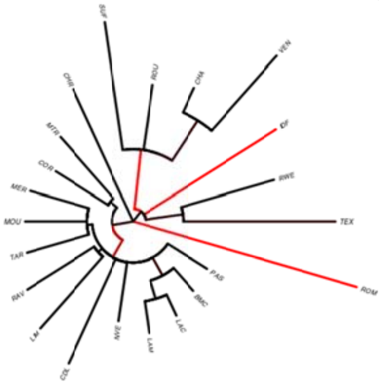

Haplotype local tree

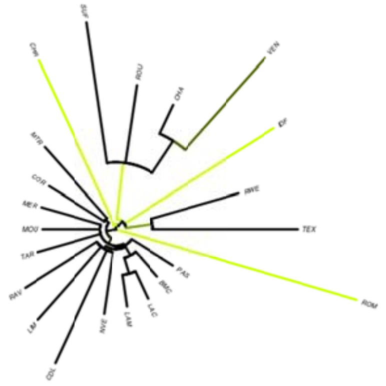

Haplotype cluster frequencies

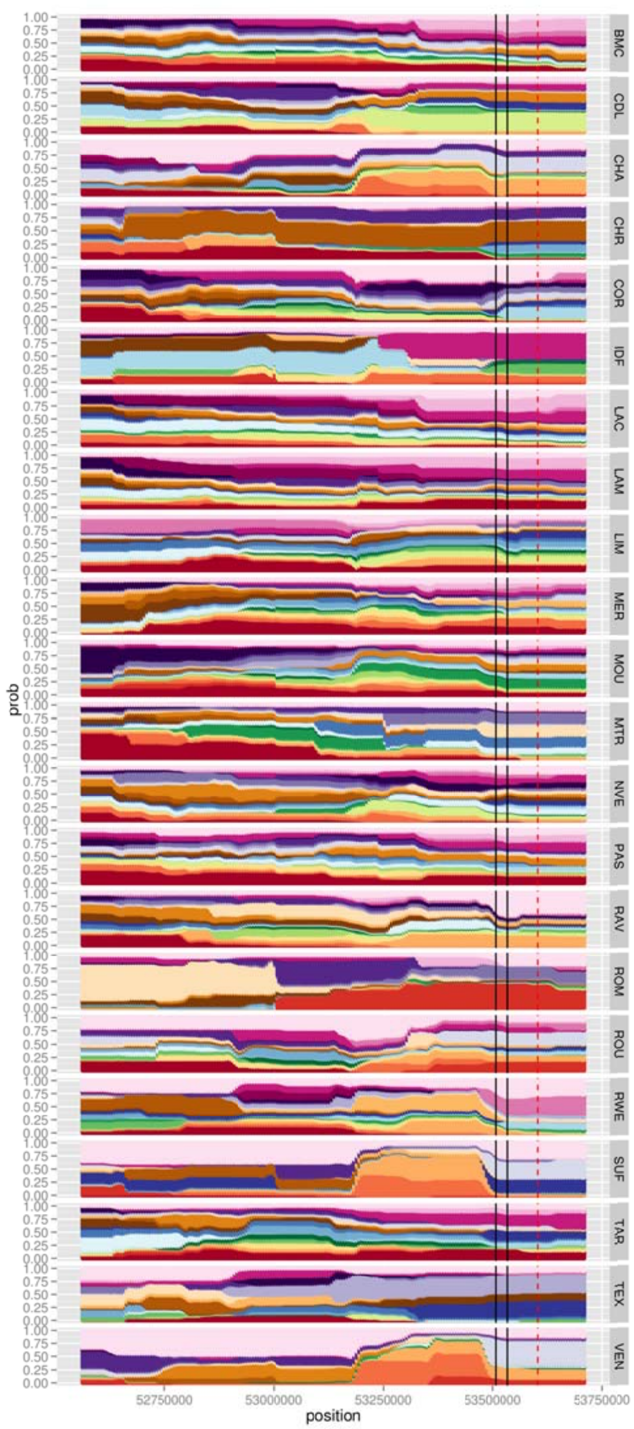

Region		
**Chr**	**Begin**	**End**
11	25369914	27740997

Best SNP: 26333173
Located in *ALOX12* [1].

SNP frequencies

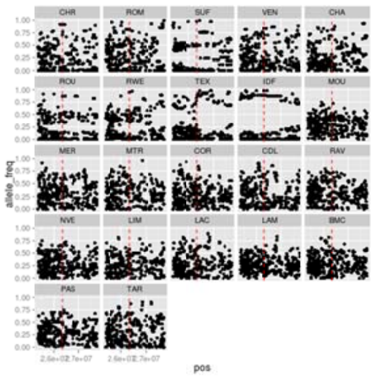

SNP local tree

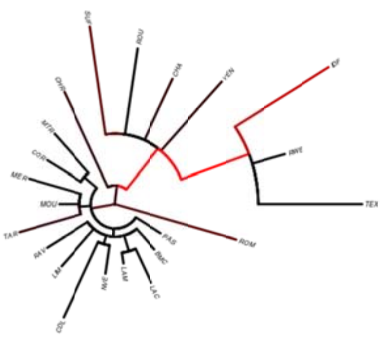

Haplotype local tree

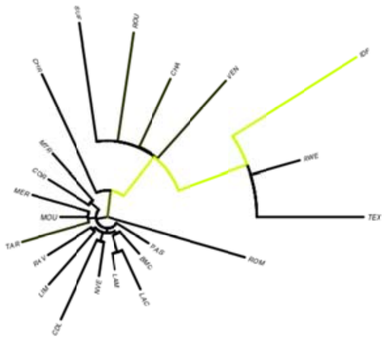

Haplotype cluster frequencies

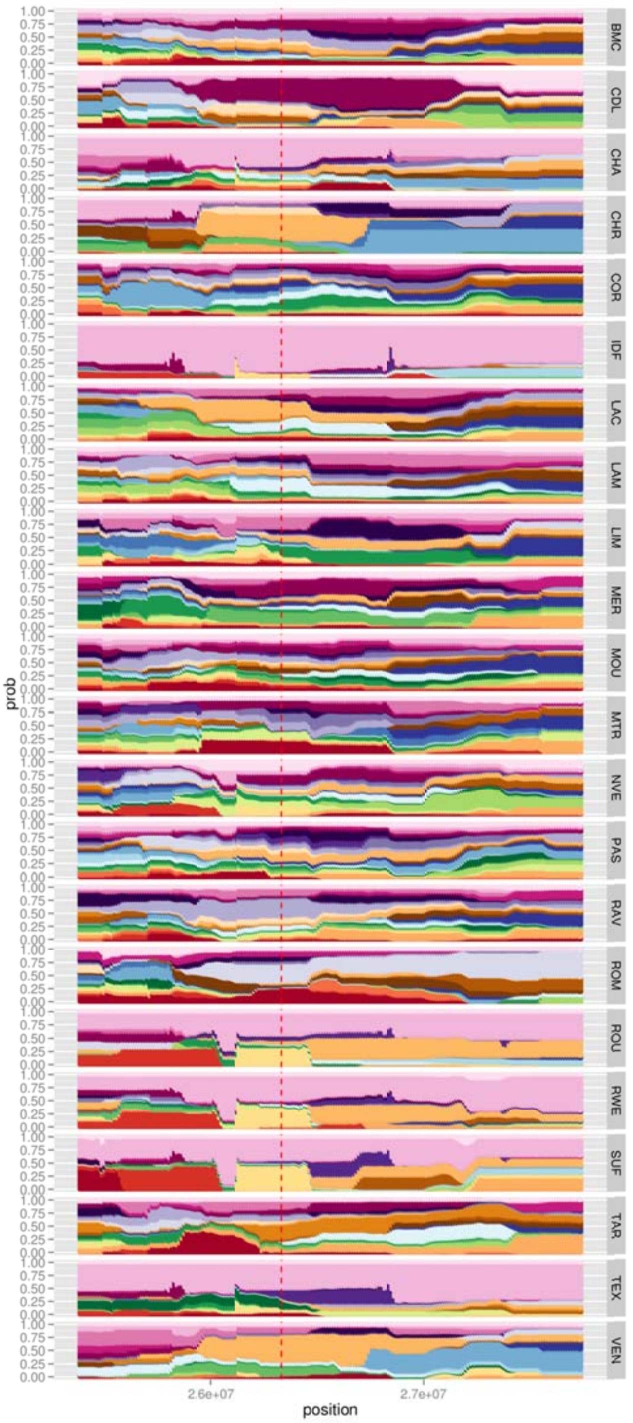

Region		
**Chr**	**Begin**	**End**
13	34108089	37740655

Best SNP: 37524442

SNP frequencies

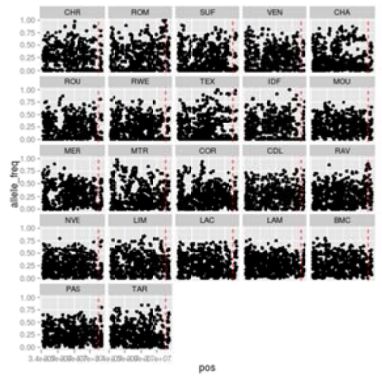

SNP local tree

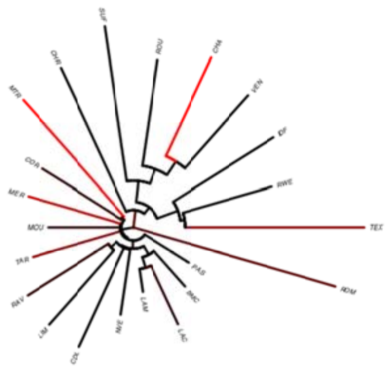

Haplotype local tree

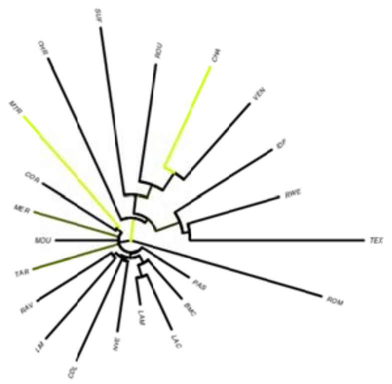

Haplotype cluster frequencies

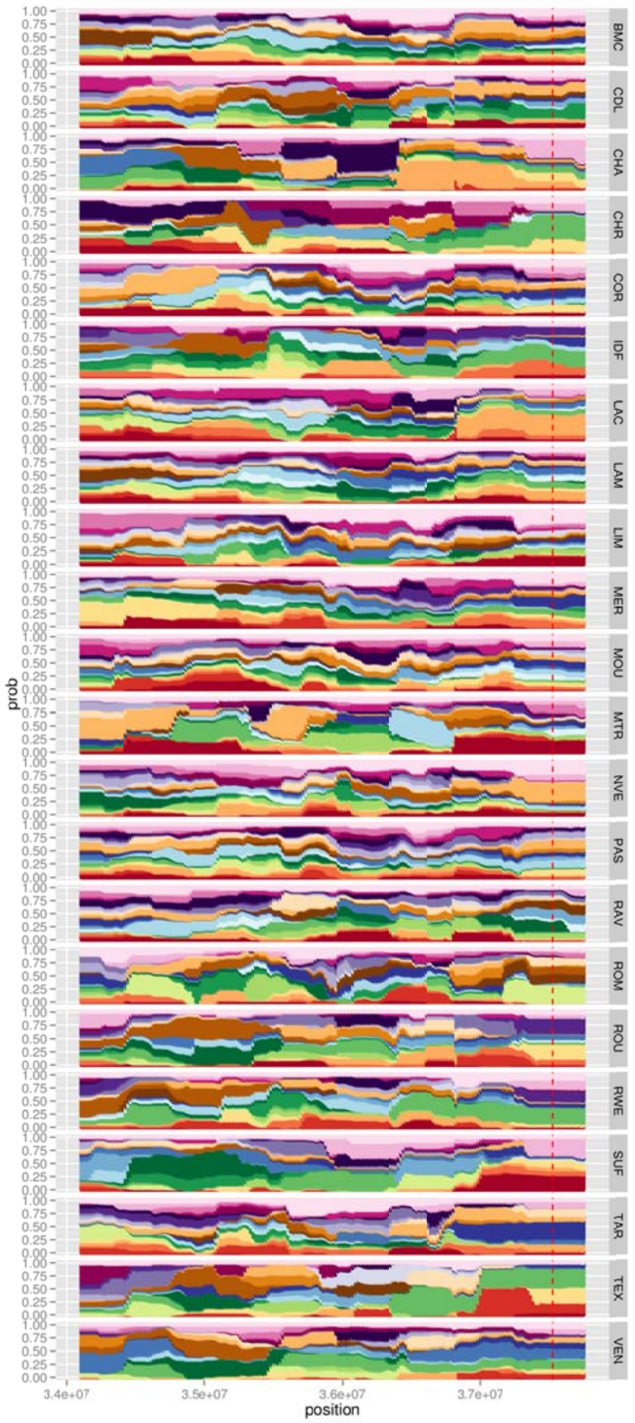

Region		
Chr	Begin	End
13	38955039	48646582

Best SNP: 46759682
Located in *pcna* [1].

SNP frequencies

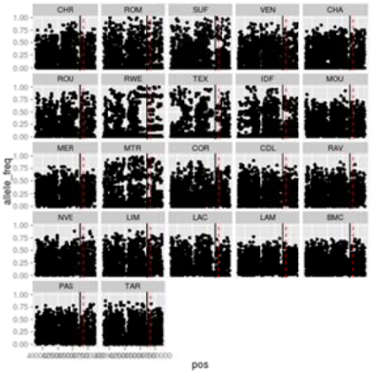

SNP local tree

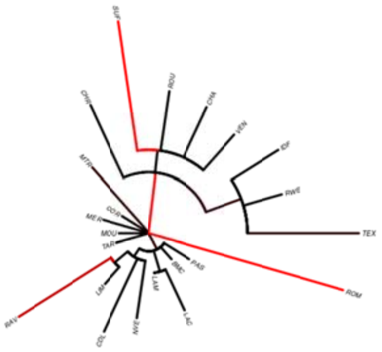

Haplotype local tree

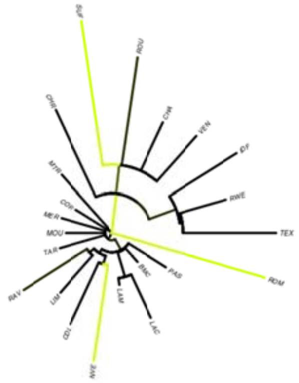

Haplotype cluster frequencies

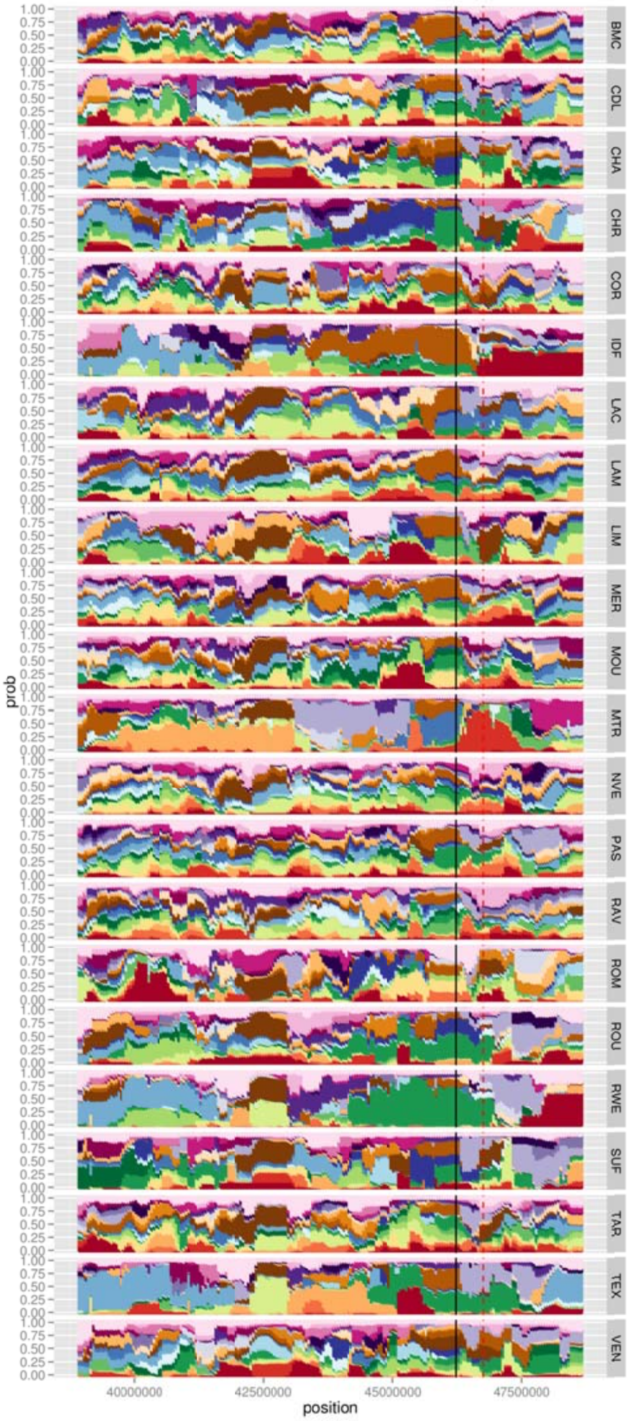

Region		
**Chr**	**Begin**	**End**
13	51357557	56499047

Best SNP: 56389183
Located in candidate gene *EDN3* [1] (involved in melanocyte development and differentiation [13]).

SNP frequencies

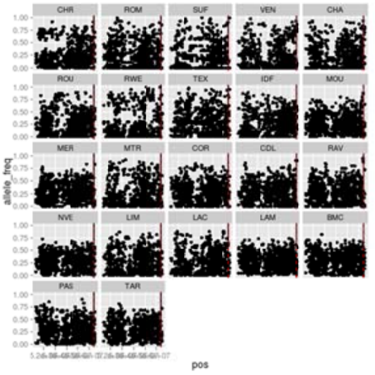

SNP local tree

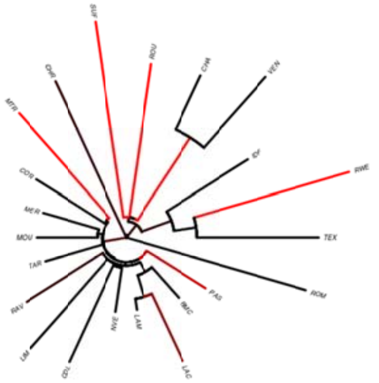

Haplotype local tree

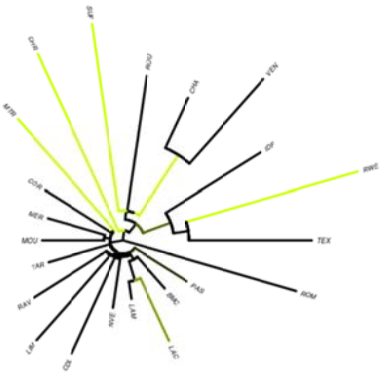

Haplotype cluster frequencies

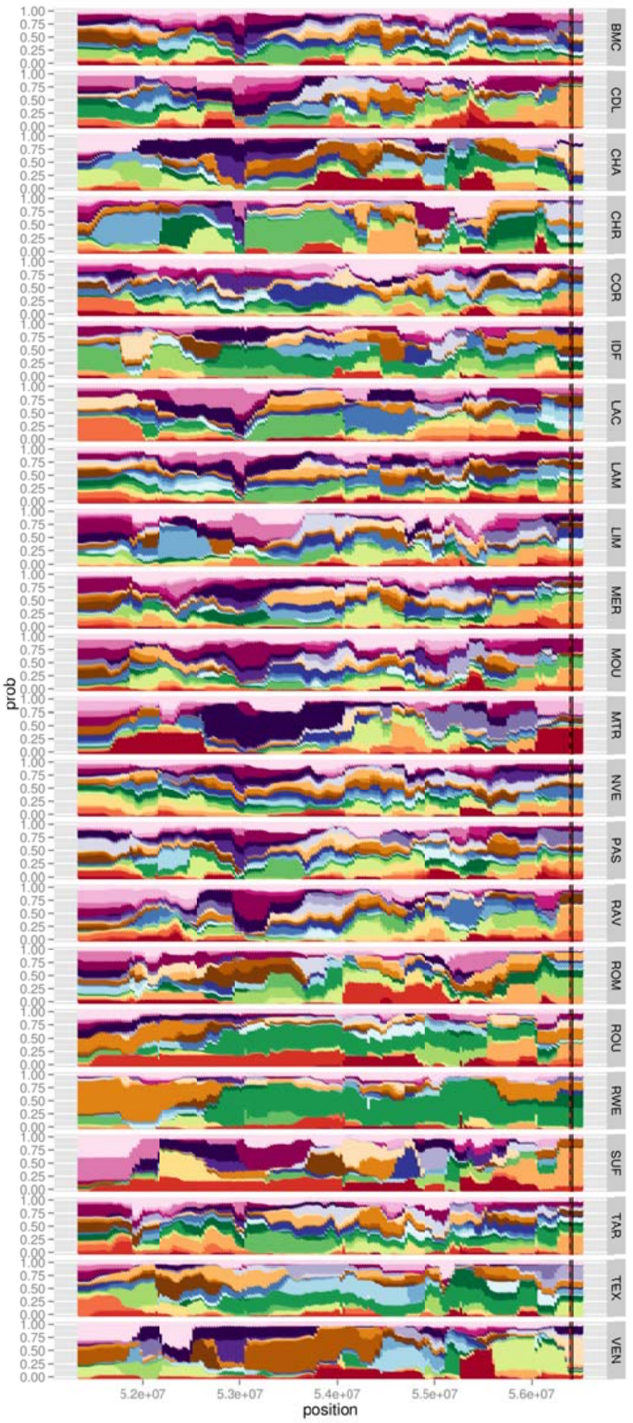

Region		
**Chr**	**Begin**	**End**
13	61688330	65155663

Best SNP: 62839711
Located in *RALY* [1].
Candidate gene: *ASIP* (involved in melanin production [14]).

SNP frequencies

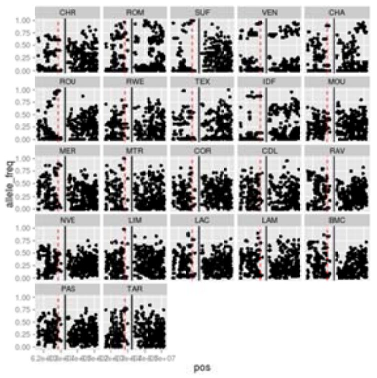

SNP local tree

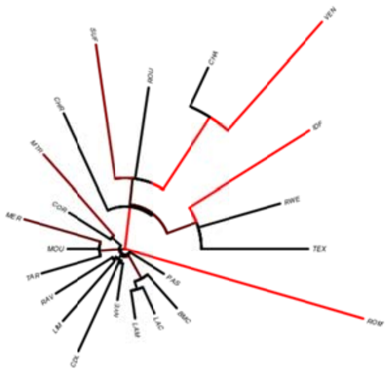

Haplotype local tree

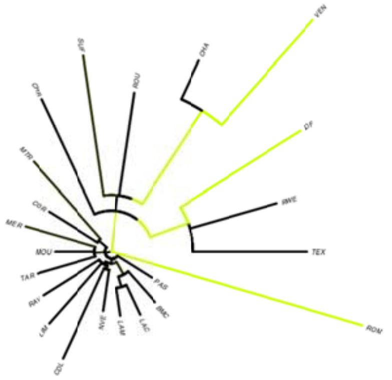

Haplotype cluster frequencies

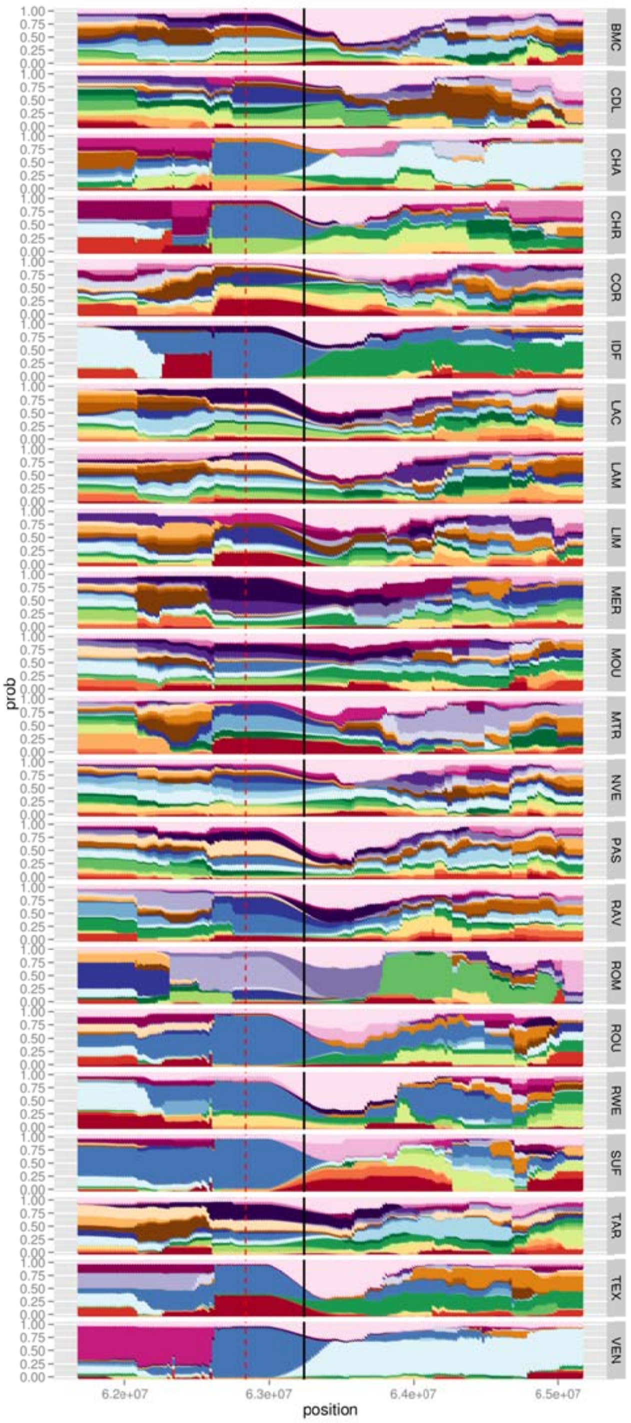

Region		
Chr	Begin	End
14	13538464	15205966

Best SNP: 14193369
Located in *SPIRE2* [1].
Candidate gene *MC1R* (involved in melanin production [14]).

SNP frequencies

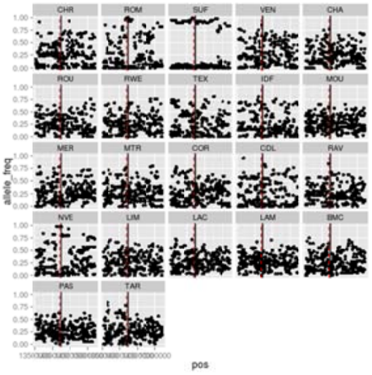

SNP local tree

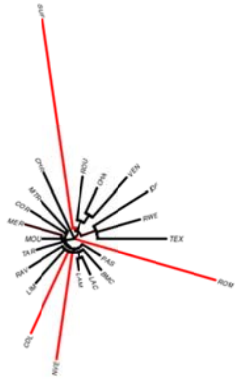

Haplotype local tree

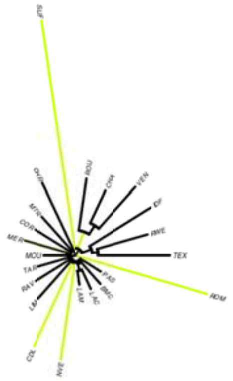

Haplotype cluster frequencies

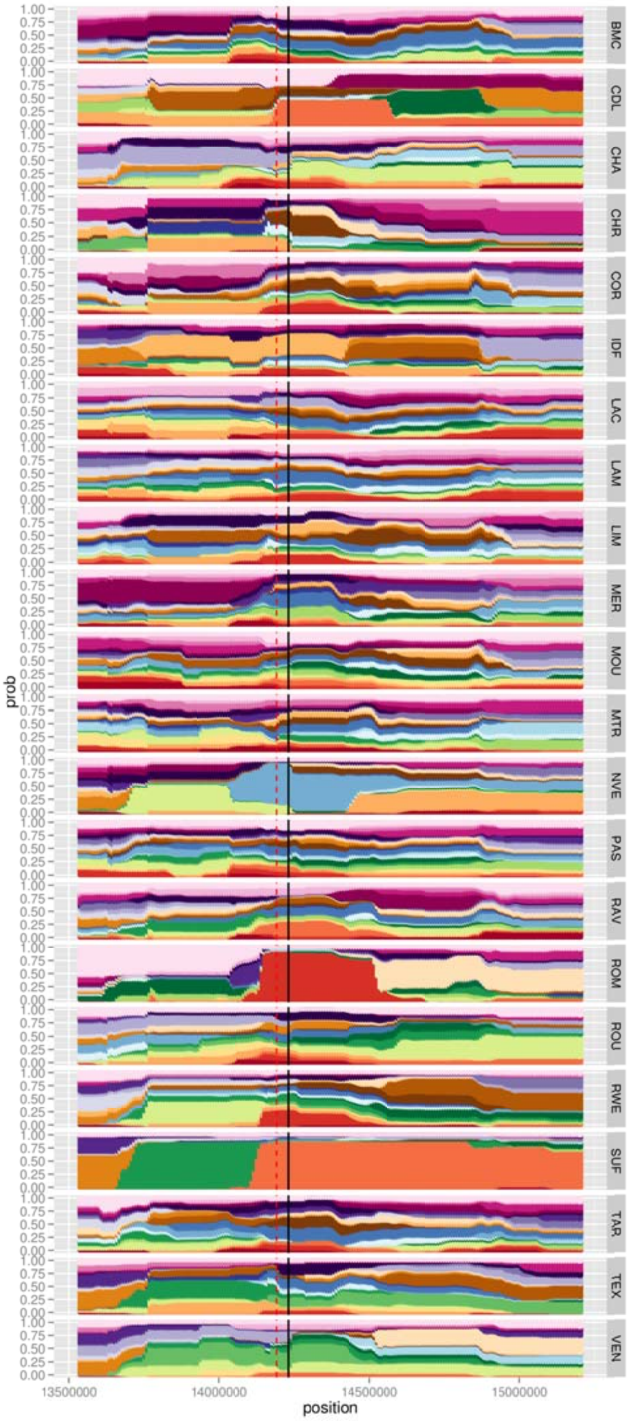

Region		
Chr	Begin	End
14	24961598	26974355

Best SNP: 26000318

SNP frequencies

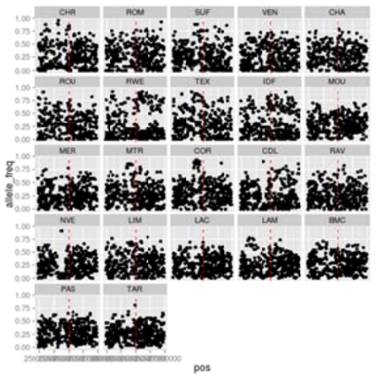

SNP local tree

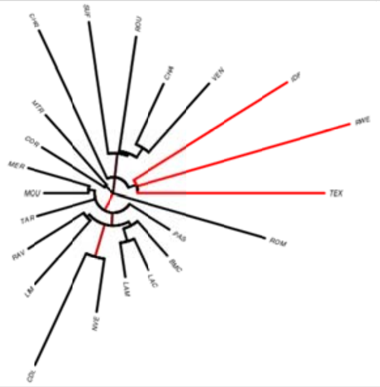

Haplotype local tree

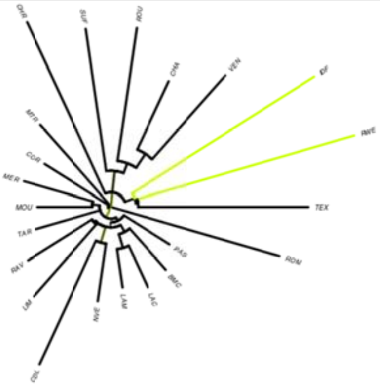

Haplotype cluster frequencies

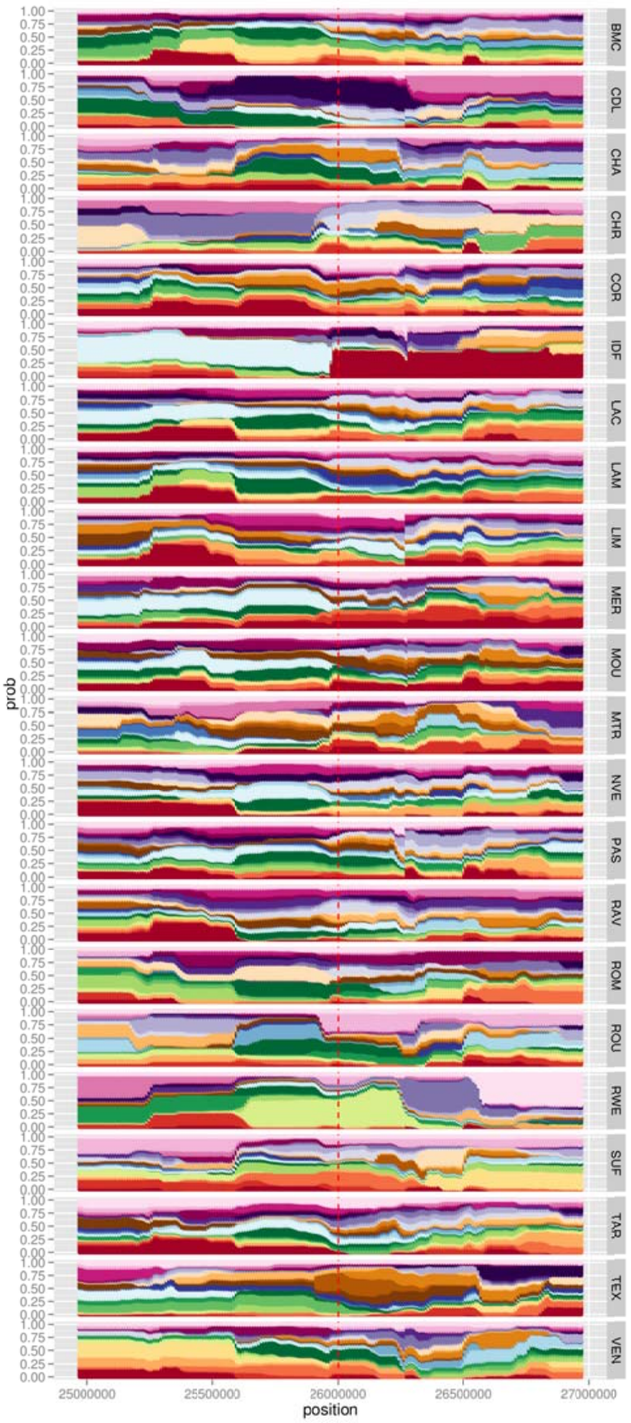

Region		
Chr	Begin	End
14	51491696	52169340

Best SNP: 51634415
Located in *BCAM* [1].

SNP frequencies

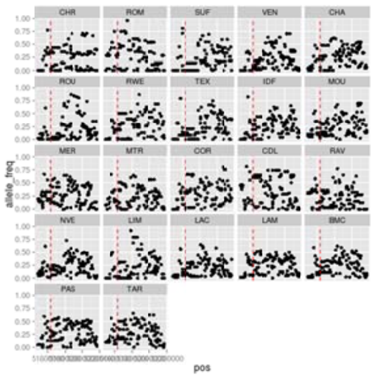

SNP local tree

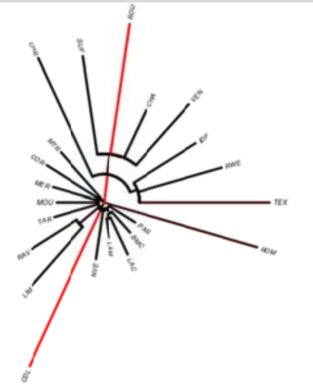

Haplotype local tree

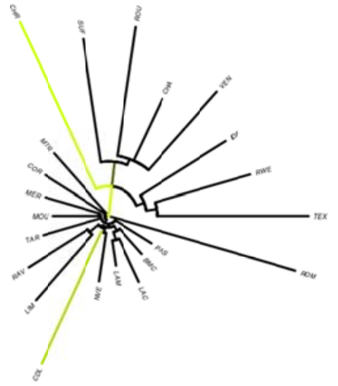

Haplotype cluster frequencies

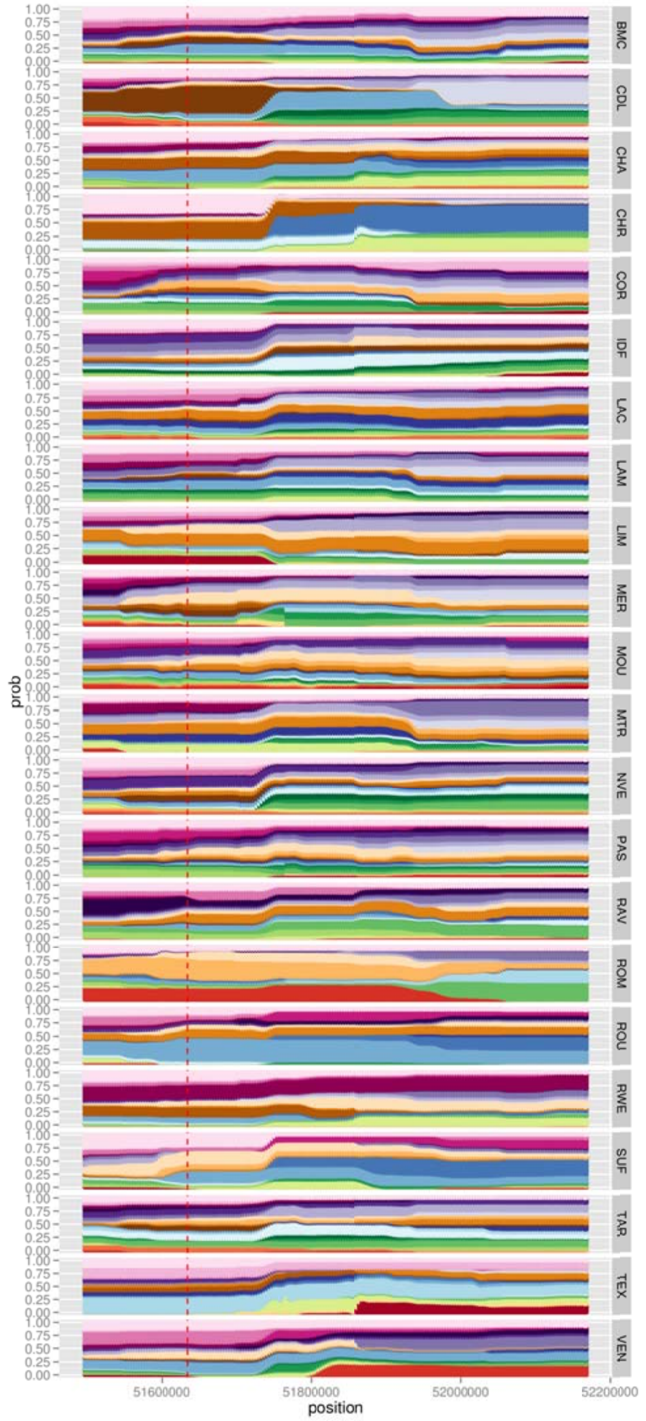

Region		
Chr	Begin	End
14	54580893	56319878

Best SNP: 55094151
Located in *IRF3* [1].

SNP frequencies

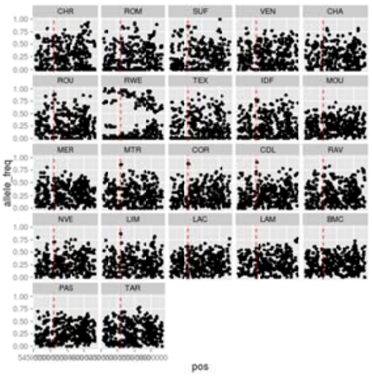

SNP local tree

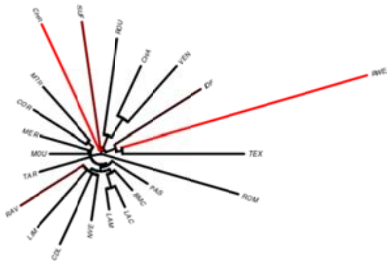

Haplotype local tree

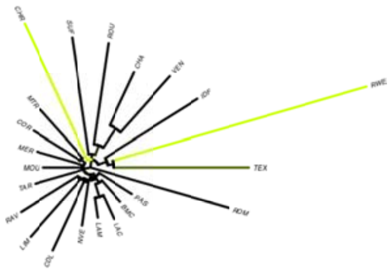

Haplotype cluster frequencies

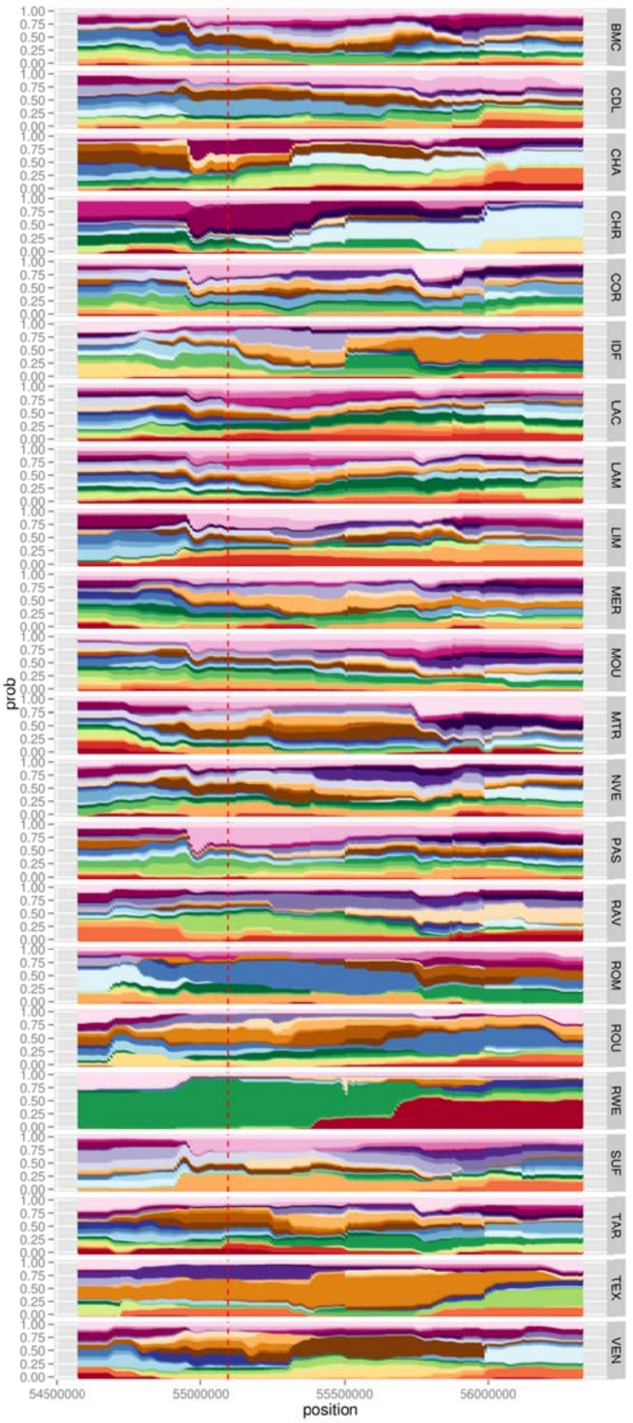

Region		
**Chr**	**Begin**	**End**
15	71351365	72991175

Best SNP: 72735040
Candidate genes: *ALX4* and *EXT2* (involved in limb and skeletal development [15]).

SNP frequencies

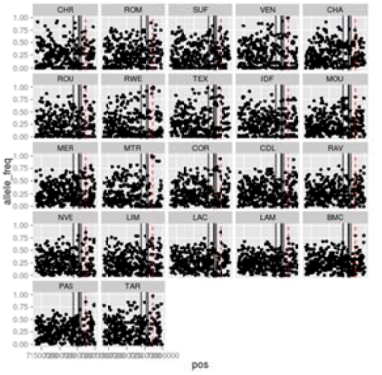

SNP local tree

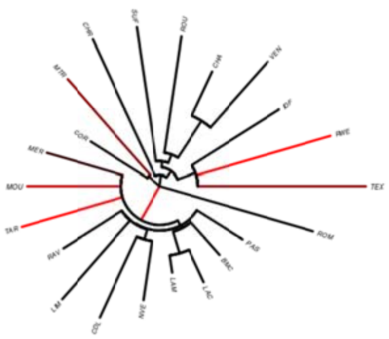

Haplotype local tree

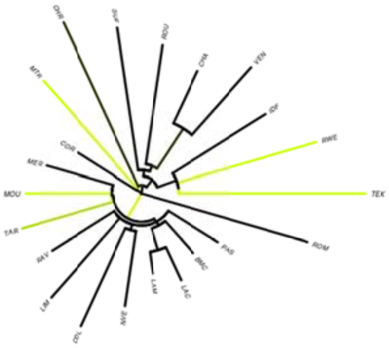

Haplotype cluster frequencies

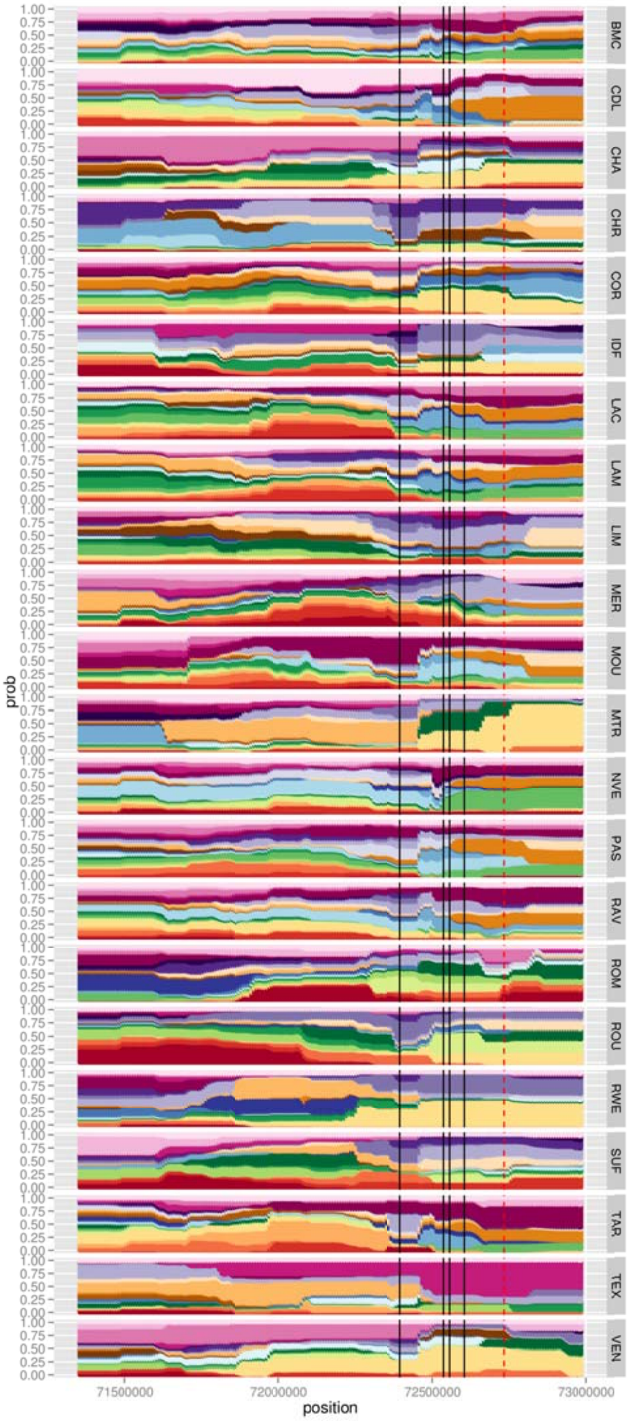

Region		
**Chr**	**Begin**	**End**
16	21691437	24960722

Best SNP: 23137683
Located in *Ankrd55* [1].

SNP frequencies

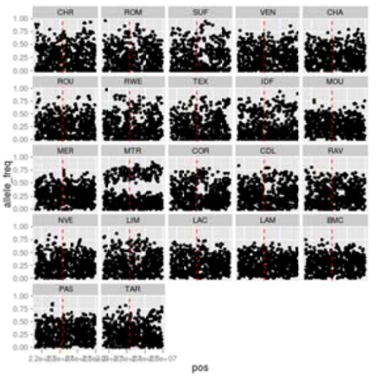

SNP local tree

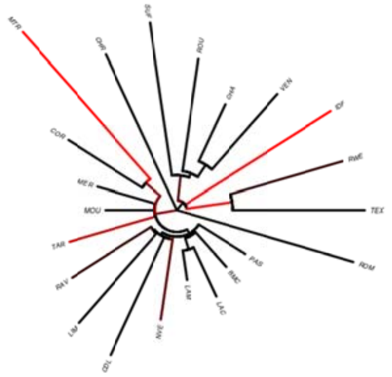

Haplotype local tree

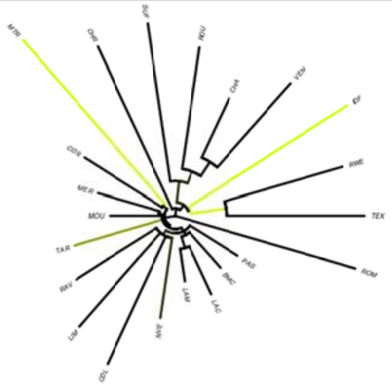

Haplotype cluster frequencies

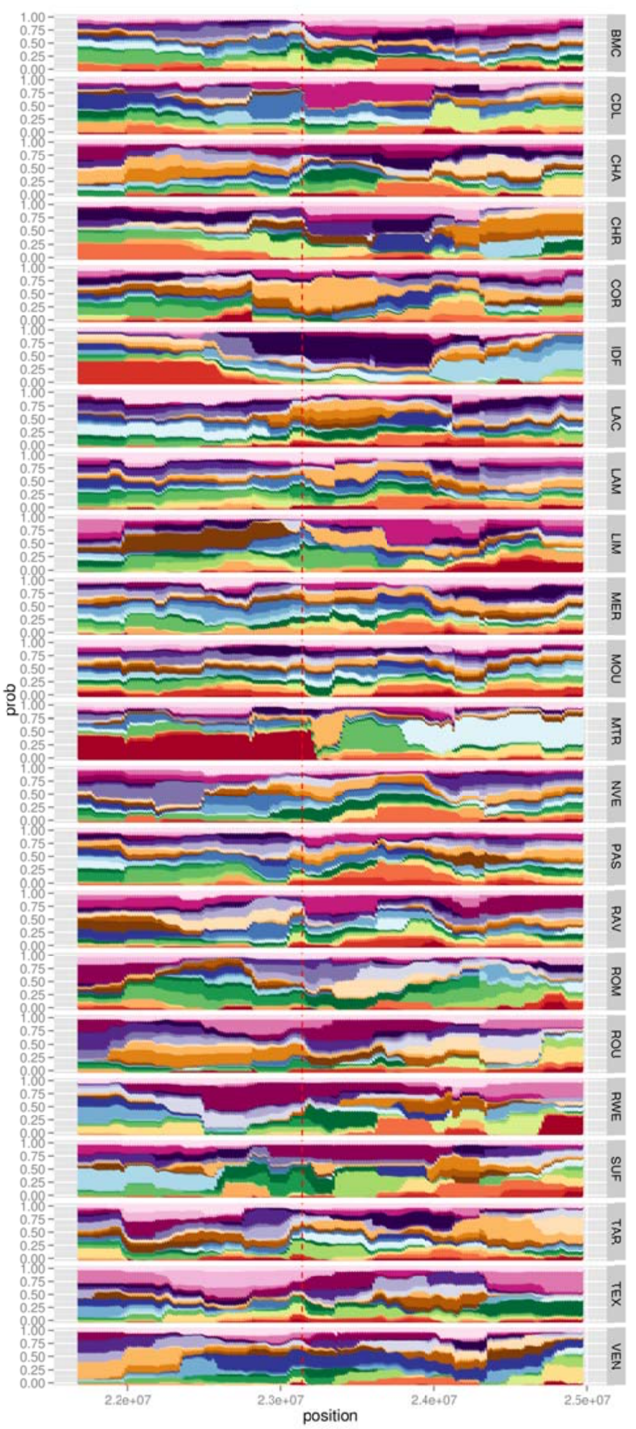

Region			SNP frequencies	Haplotype cluster frequencies
**Chr**	**Begin**	**End**		
16	31871071	33297458		

Best SNP: 32772136
Located in *OXCT1* [1]
(associated with milk fatty acid traits in dairy cattle [16]).

SNP frequencies

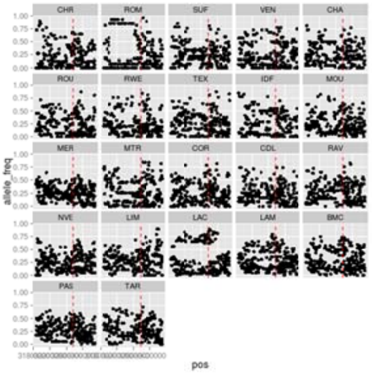

SNP local tree

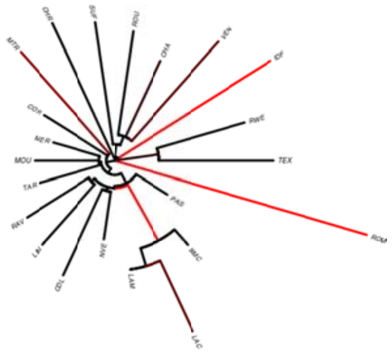

Haplotype local tree

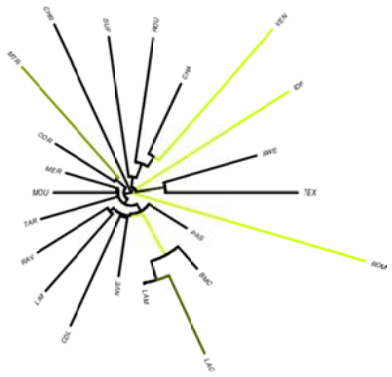

Haplotype cluster frequencies

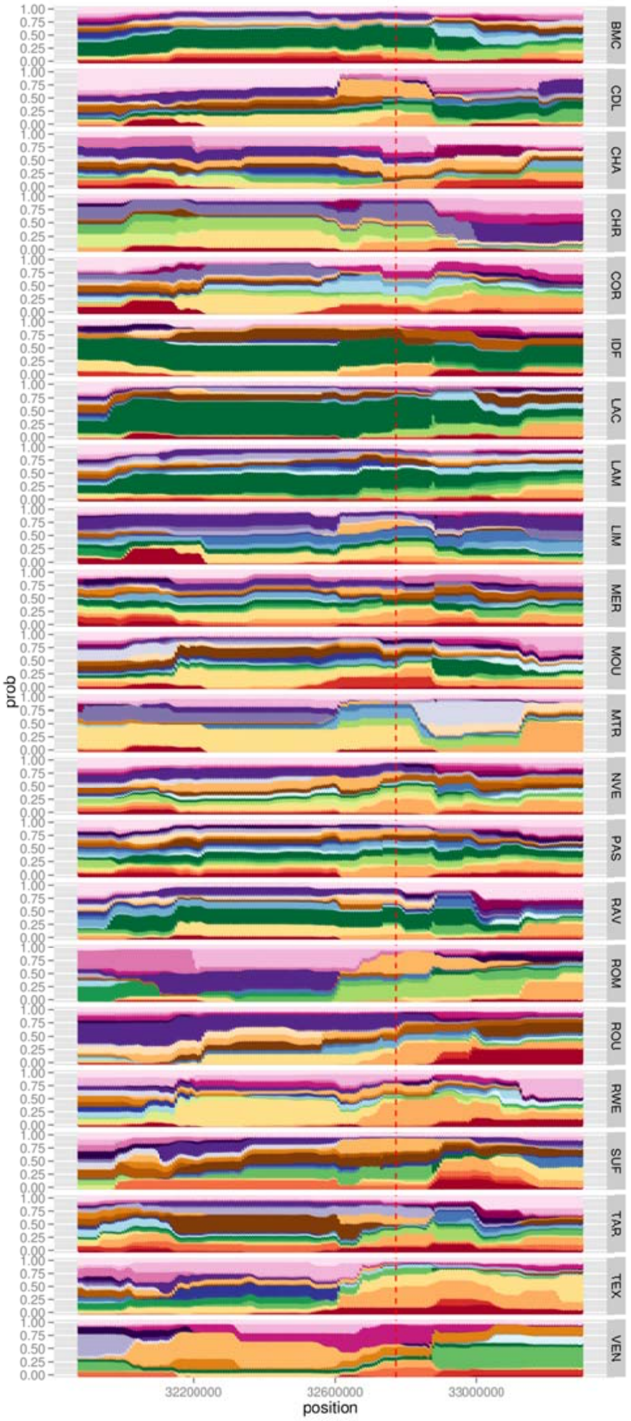

Region		
**Chr**	**Begin**	**End**
16	38429345	40246375

Best SNP: 38664205

SNP frequencies

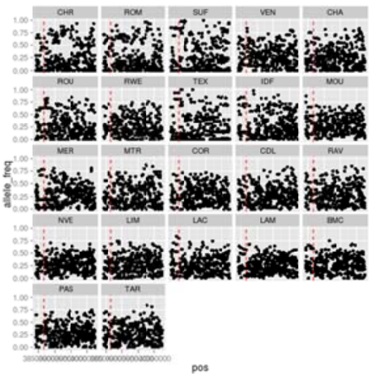

SNP local tree

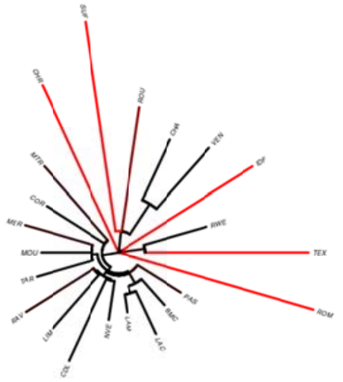

Haplotype local tree

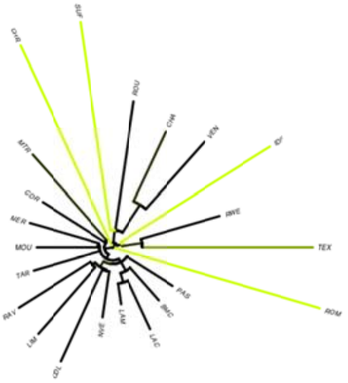

Haplotype cluster frequencies

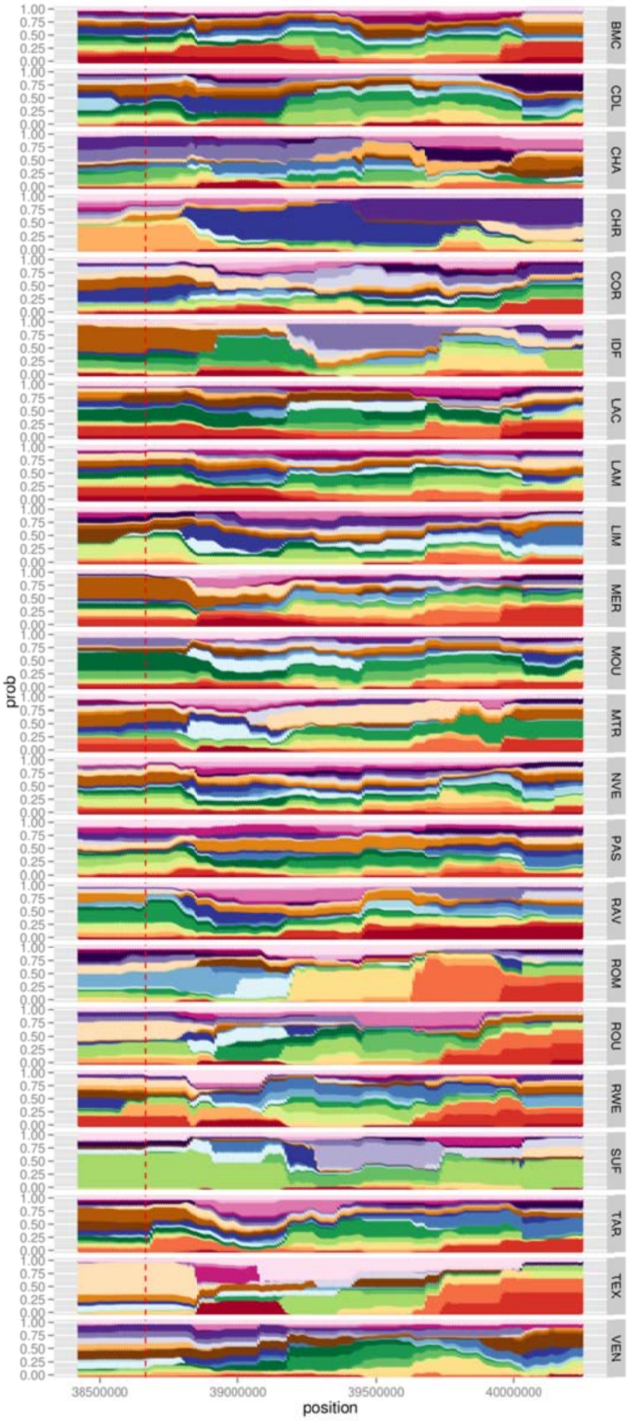

Region		
**Chr**	**Begin**	**End**
19	30571699	33417522

Best SNP: 31666785
Located in candidate gene: *MITF* (involved in melanocyte development [17]).

SNP frequencies

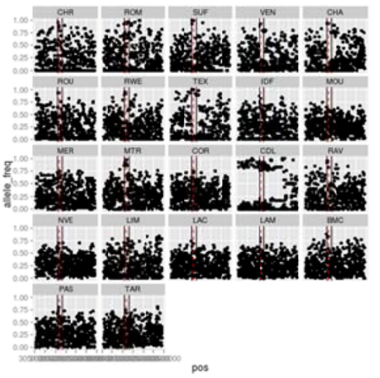

SNP local tree

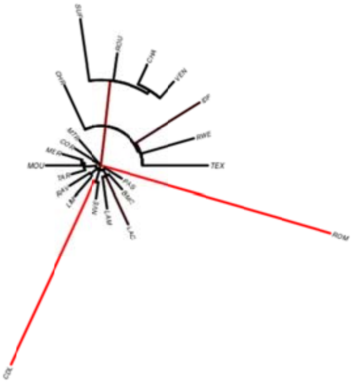

Haplotype local tree

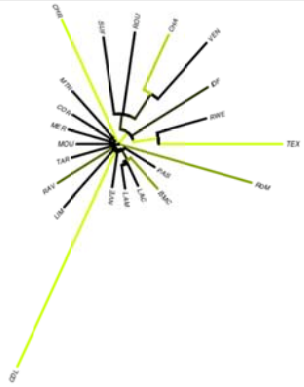

Haplotype cluster frequencies

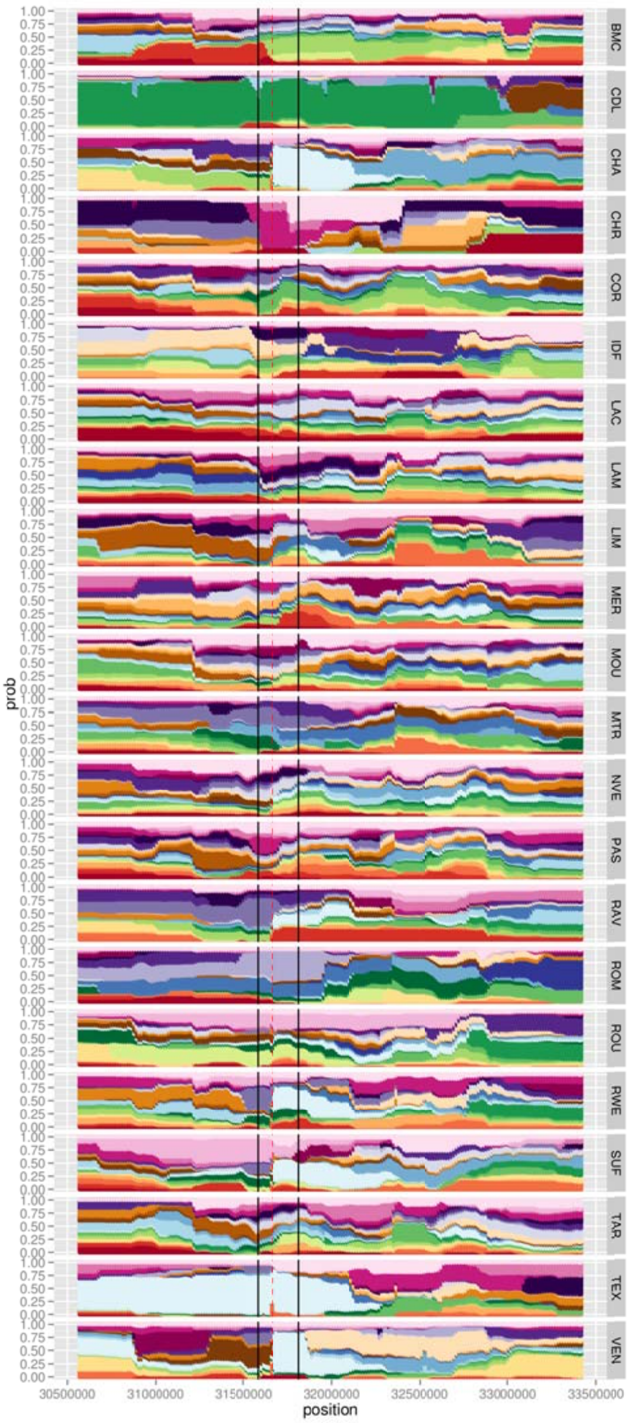

Region		
Chr	Begin	End
19	36538771	36657385

Best SNP: 36584288
Located in candidate gene *ADAMTS9* [1] (involved both in cardiovascular development and homeostasis [18]).

SNP frequencies

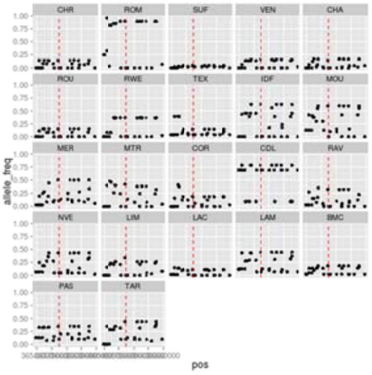

SNP local tree

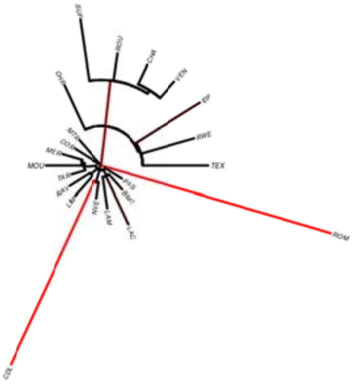

Haplotype local tree

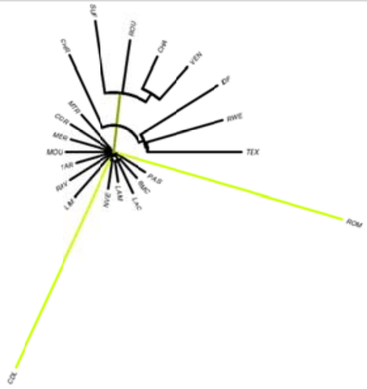

Haplotype cluster frequencies

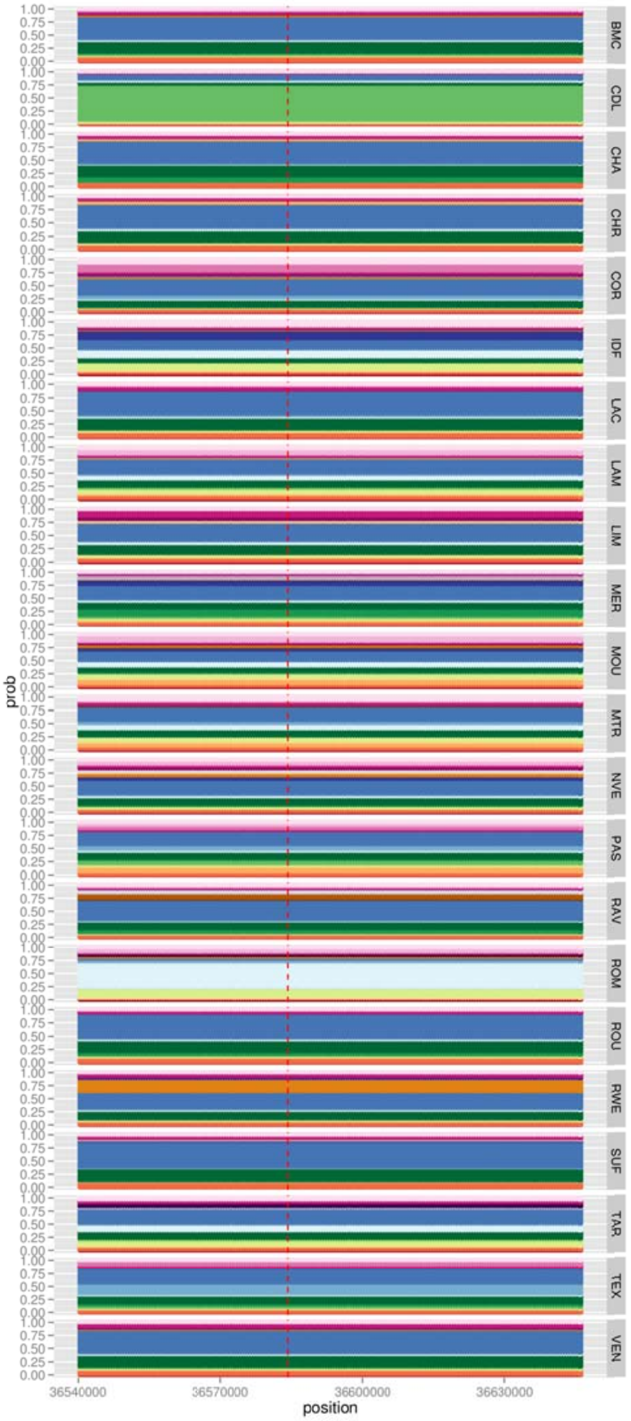

Region		
Chr	Begin	End
19	48086301	53558368

Best SNP: 48422940
Located in *SEMA3G* [1].

SNP frequencies

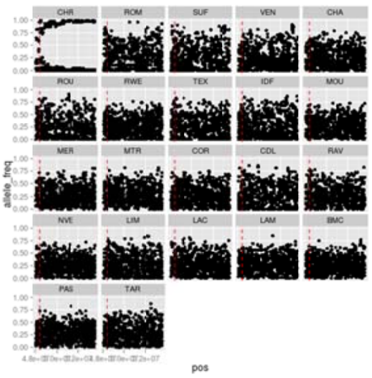

SNP local tree

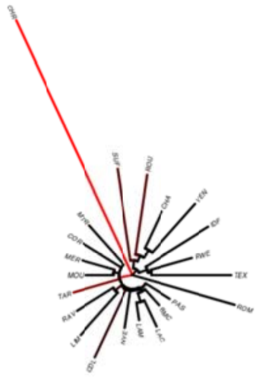

Haplotype local tree

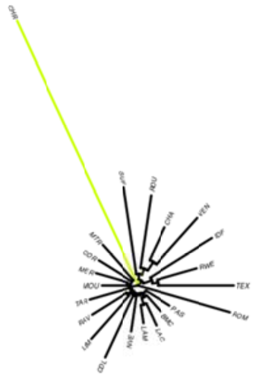

Haplotype cluster frequencies

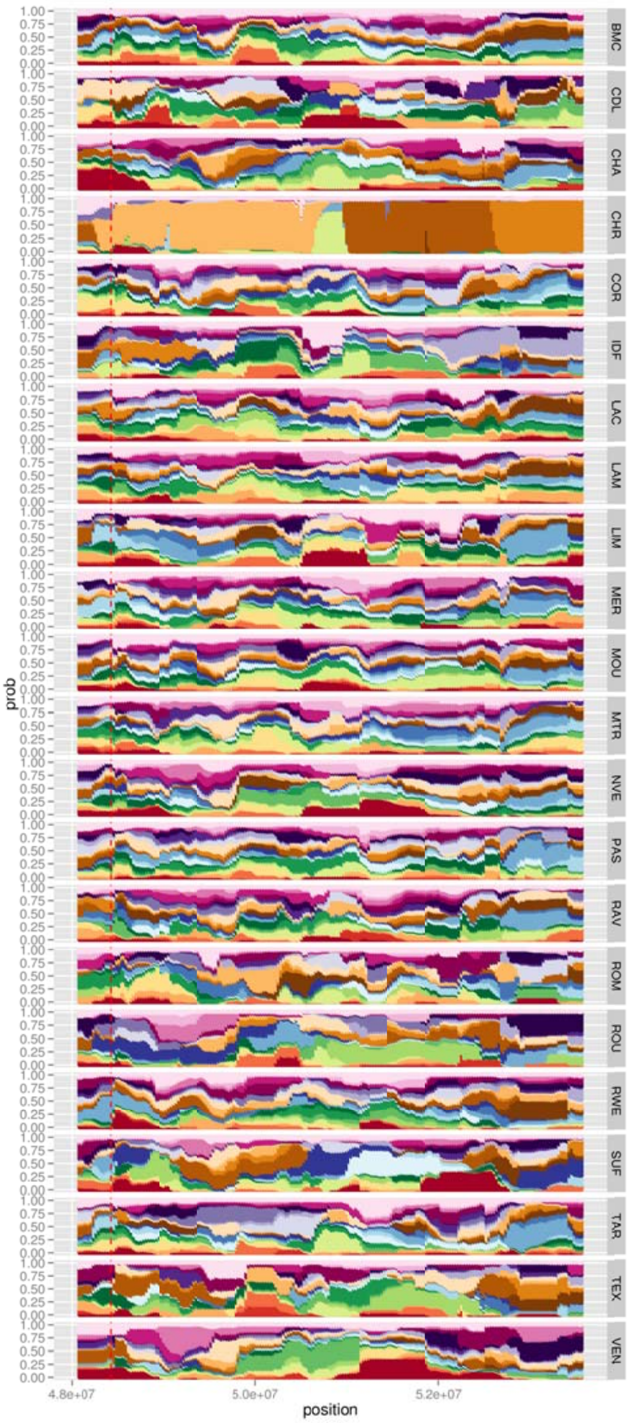

Region		
Chr	Begin	End
21	48425709	49872592

Best SNP: 49553506

SNP frequencies

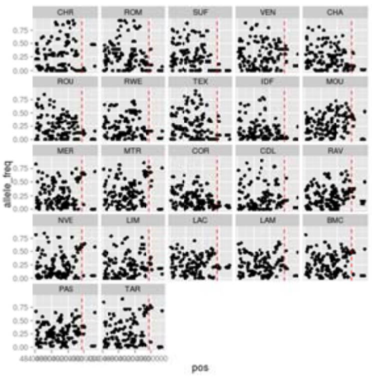

SNP local tree

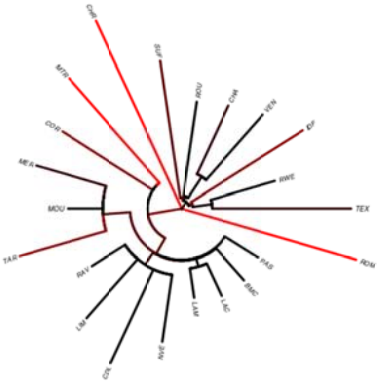

Haplotype local tree

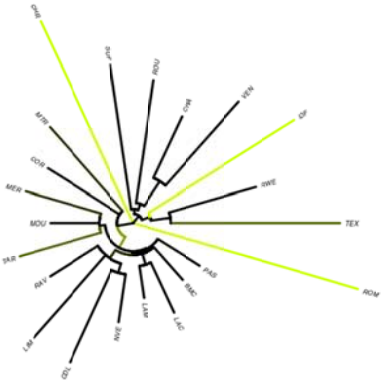

Haplotype cluster frequencies

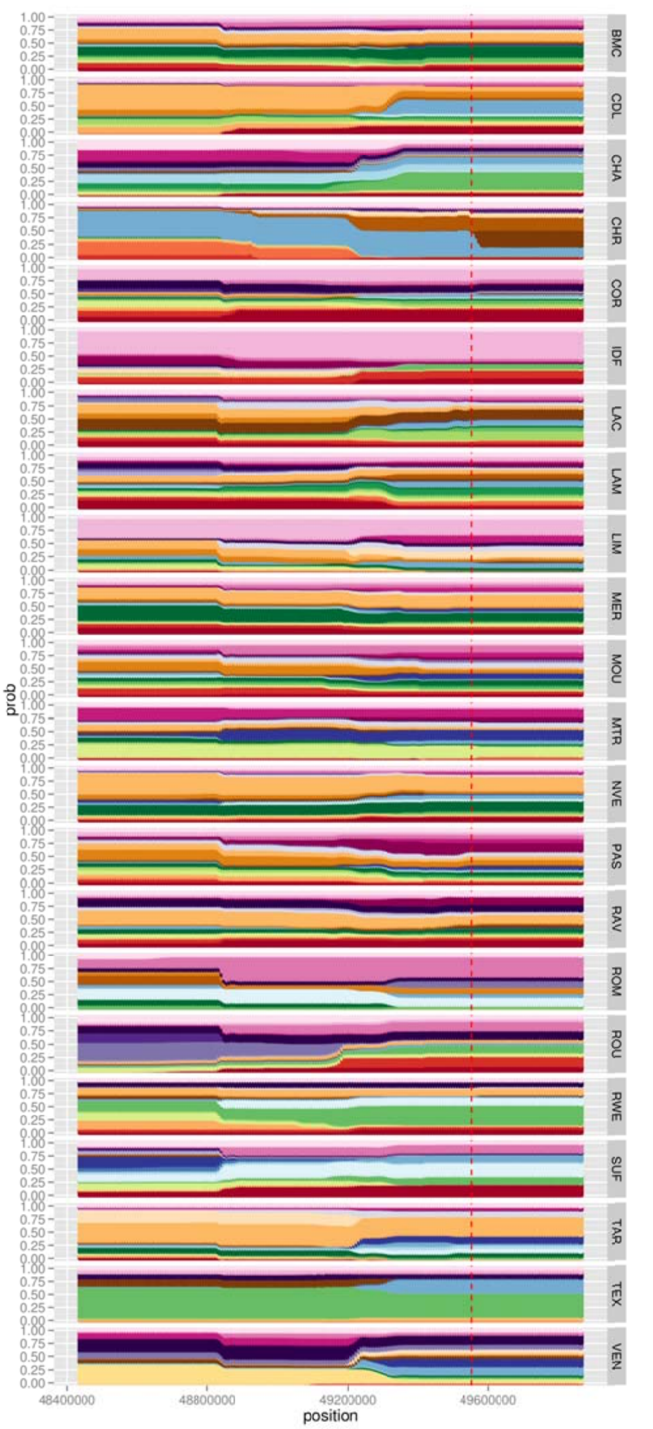

Region		
**Chr**	**Begin**	**End**
24	9946422	10609666

Best SNP: 10590023

SNP frequencies

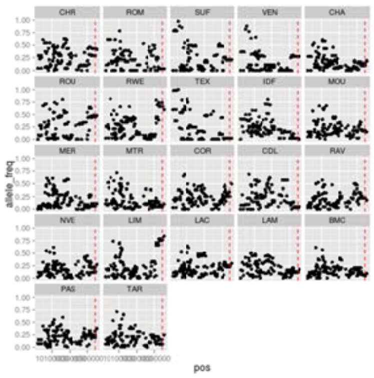

SNP local tree

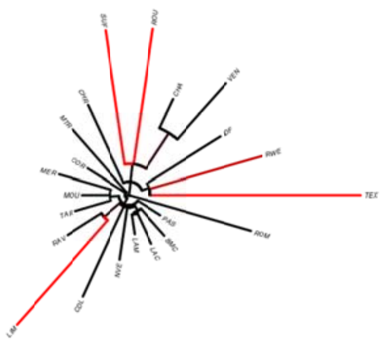

Haplotype local tree

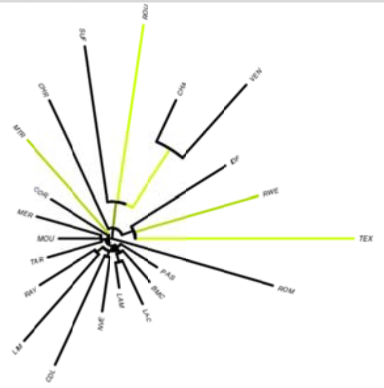

Haplotype cluster frequencies

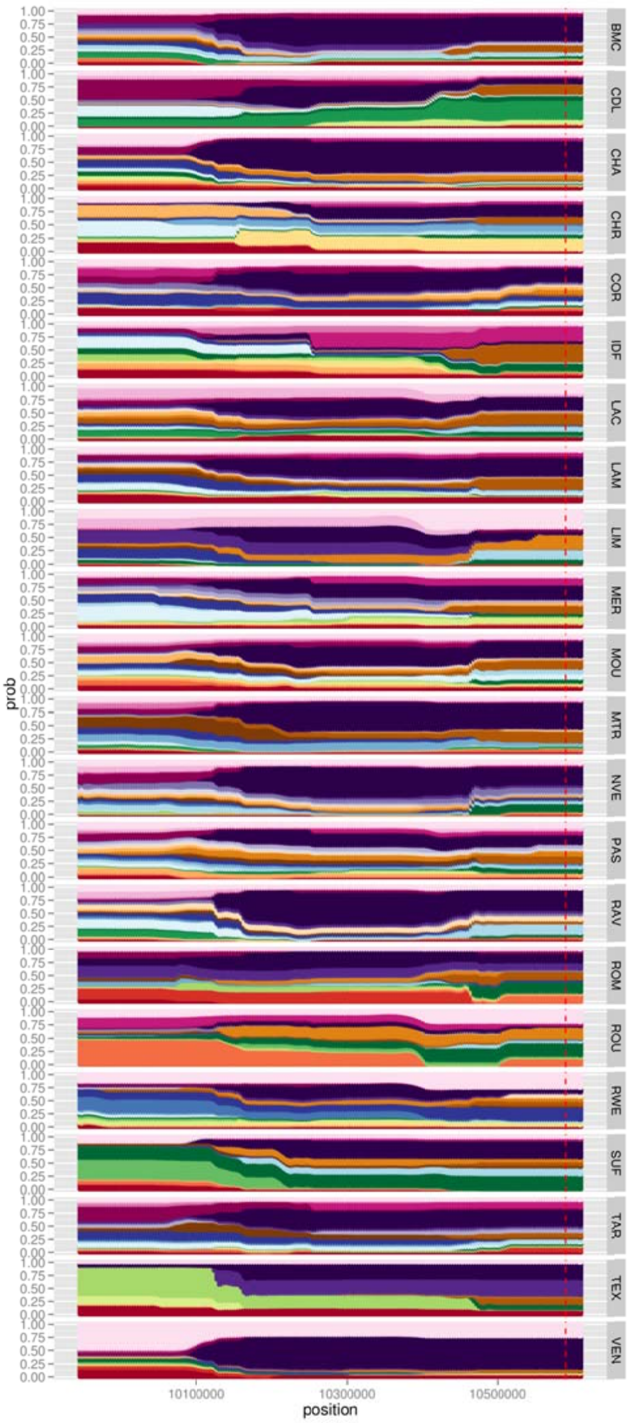

Region		
**Chr**	**Begin**	**End**
25	6698813	8658779

Best SNP: 7489792
Candidate gene *IRF2BP2* (associated with fleece variation in sheep [19]).

SNP frequencies

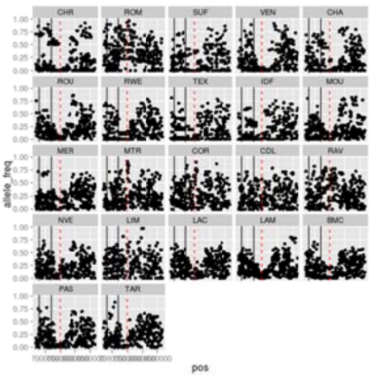

SNP local tree

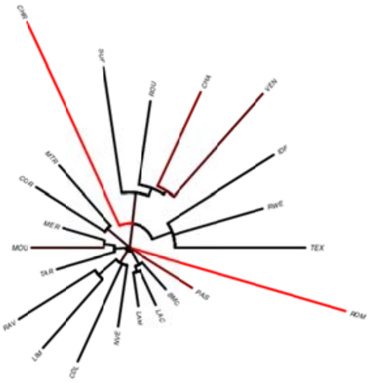

Haplotype local tree

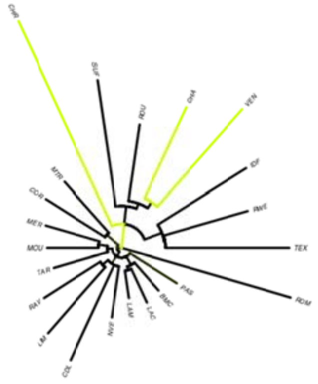

Haplotype cluster frequencies

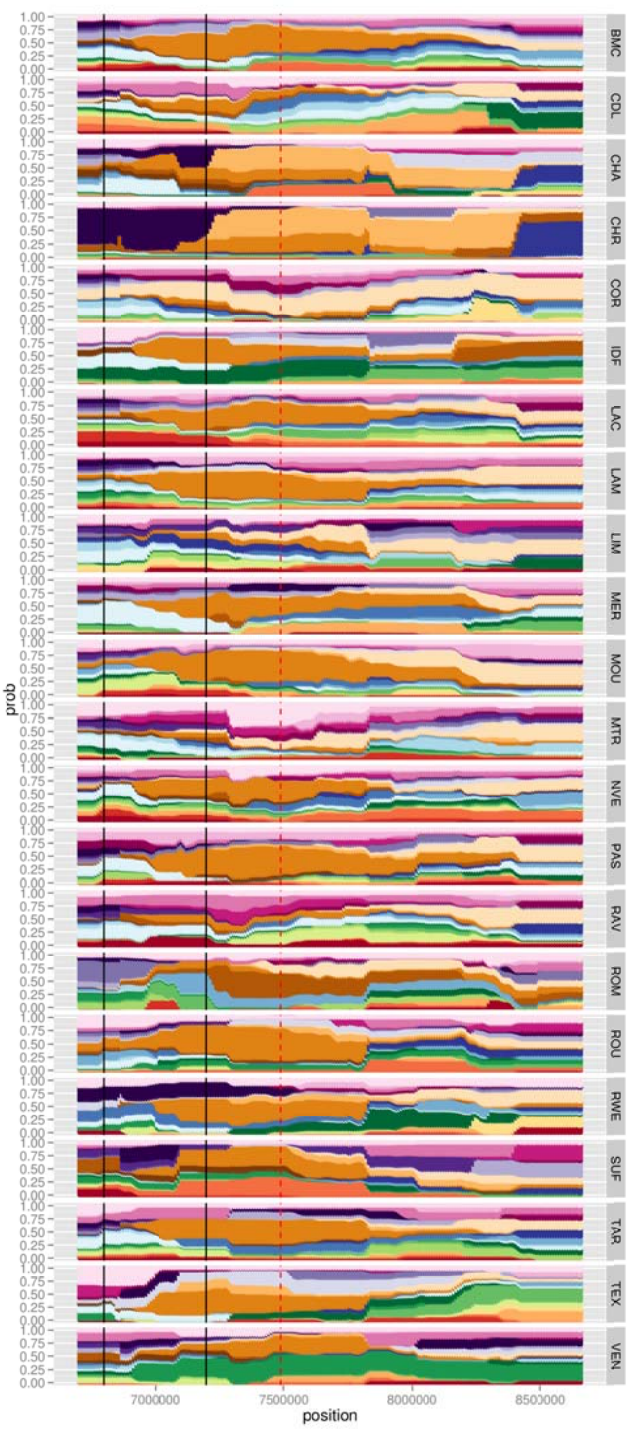

Region		
**Chr**	**Begin**	**End**
25	23213400	26042495

Best SNP: 24991189
Located in *STOX1* [1].

SNP frequencies

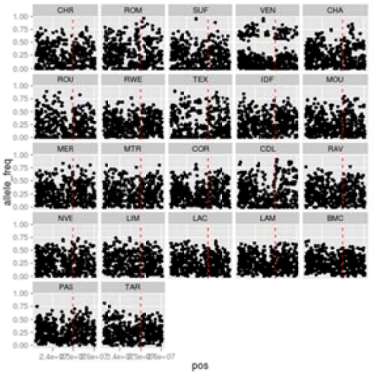

SNP local tree

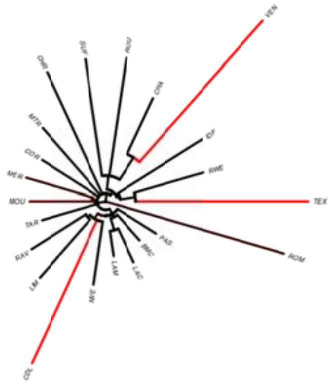

Haplotype local tree

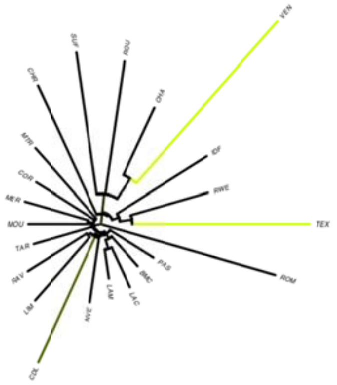

Haplotype cluster frequencies

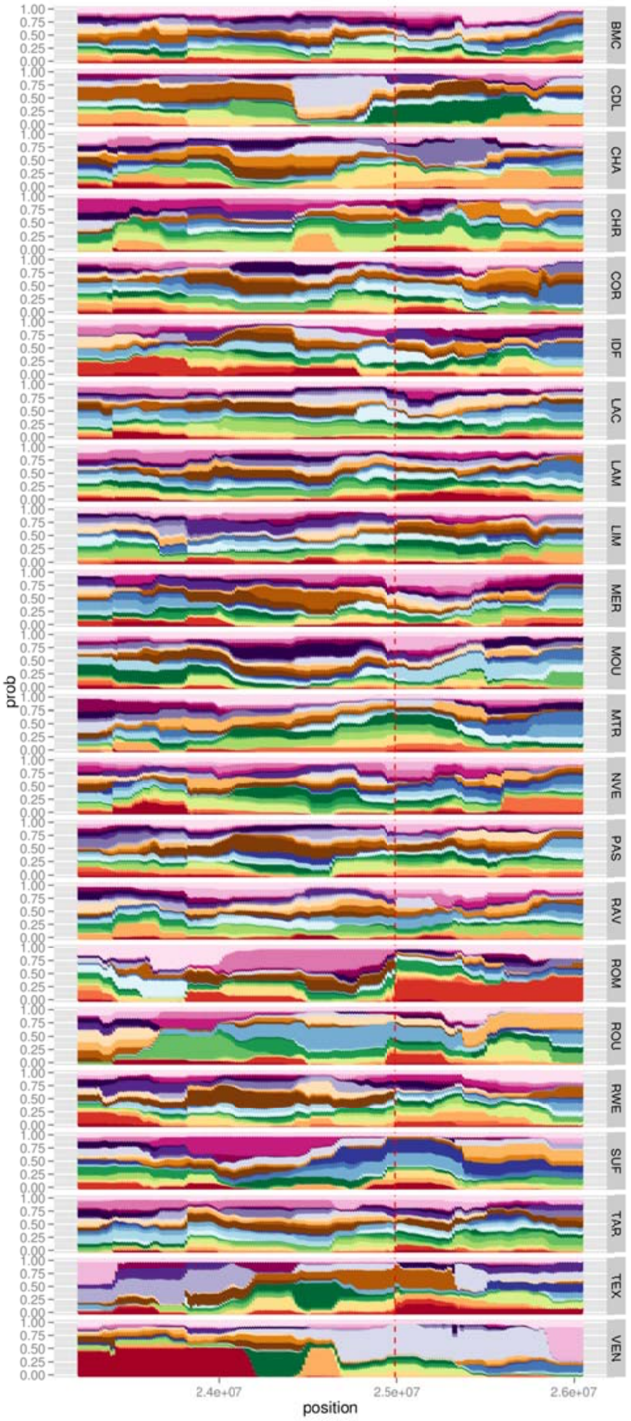

References

1. University of California Santa Cruz. UCSC Genome Browser Gateway. http://genome-euro.ucsc.edu/cgi-bin/hgGateway?redirect=manual&source=genome.ucsc.edu.

2. Rajakumari S, Wu J, Ishibashi J, Lim HW, Giang AH, Won KJ, et al. EBF2 determines and maintains brown adipocyte identity. Cell Metab. 2013;17:562–74. doi:10.1016/j.cmet.2013.01.015.

3. Vasques GA, Amano N, Docko AJ, Funari MFA, Quedas EPS, Nishi MY, et al. Heterozygous mutations in natriuretic peptide receptor-B (NPR2) gene as a cause of short stature in patients initially classified as idiopathic short stature. J Clin Endocrinol Metab. 2013;98:1636–44.

4. Gratten J, Beraldi D, Lowder B V, McRae a F, Visscher PM, Pemberton JM, et al. Compelling evidence that a single nucleotide substitution in TYRP1 is responsible for coat-colour polymorphism in a free-living population of Soay sheep. Proc Biol Sci. 2007;274:619–26.

5. Jacobs LC, Wollstein A, Lao O, Hofman A, Klaver CC, Uitterlinden AG, et al. Comprehensive candidate gene study highlights UGT1A and BNC2 as new genes determining continuous skin color variation in Europeans. Hum Genet. 2013;132:147–58.

6. Rico-Bautista E, Flores-Morales A, Fernández-Pérez L. Suppressor of cytokine signaling (SOCS) 2, a protein with multiple functions. Cytokine Growth Factor Rev. 2006;17:431–9.

7. Rupp R, Senin P, Sarry J, Allain C, Tasca C, Ligat L, et al. A Point Mutation in Suppressor of Cytokine Signalling 2 (Socs2) Increases the Susceptibility to Inflammation of the Mammary Gland while Associated with Higher Body Weight and Size and Higher Milk Production in a Sheep Model. PLoS Genet. 2015;11:1–19. doi:10.1371/journal.pgen.1005629.

8. Jones P, Chase K, Martin A, Davern P, Ostrander EA, Lark KG. Single-nucleotide-polymorphism-based association mapping of dog stereotypes. Genetics. 2008;179:1033–44.

9. Zhang L, Liang J, Luo W, Liu X, Yan H, Zhao K, et al. Genome-wide scan reveals LEMD3 and WIF1 on SSC5 as the candidates for porcine ear size. PLoS One. 2014;9:1–9.

10. García-Fernández M, Gutiérrez-Gil B, Sánchez JP, Morán JA, García-Gámez E, Álvarez L, et al. The role of bovine causal genes underlying dairy traits in Spanish Churra sheep. Anim Genet. 2011;42:415–20.

11. Sahana G, Höglund JK, Guldbrandtsen B, Lund MS. Loci associated with adult stature also affect calf birth survival in cattle. BMC Genet. 2015;16:47. doi:10.1186/s12863-015-0202-3.

12. Johnston SE, McEwan JC, Pickering NK, Kijas JW, Beraldi D, Pilkington JG, et al. Genome-wide association mapping identifies the genetic basis of discrete and quantitative variation in sexual weaponry in a wild sheep population. Mol Ecol. 2011;20:2555–66.

13. Cieslak M, Reissmann M, Hofreiter M, Ludwig A. Colours of domestication. Biol Rev. 2011;86:885–99.

14. Ducrest AL, Keller L, Roulin A. Pleiotropy in the melanocortin system, coloration and behavioural syndromes. Trends Ecol Evol. 2008;23:502–10.

15. Fariello M-I, Servin B, Tosser-Klopp G, Rupp R, Moreno C, San Cristobal M, et al. Selection signatures in worldwide sheep populations. PLoS One. 2014;9:e103813.

16. Li C, Sun D, Zhang S, Wang S, Wu X, Zhang Q, et al. Genome wide association study identifies 20 novel promising genes associated with milk fatty acid traits in Chinese Holstein. PLoS One. 2014;9.

17. Levy C, Khaled M, Fisher DE. MITF: master regulator of melanocyte development and melanoma oncogene. Trends Mol Med. 2006;12:406–14.

18. Kern CB, Wessels A, McGarity J, Dixon LJ, Alston E, Argraves WS, et al. Reduced versican cleavage

due to Adamts9 haploinsufficiency is associated with cardiac and aortic anomalies. Matrix Biol. 2010;29:304–16.

19. Demars J, Cano M, Drouilhet L, Plisson-Petit F, Bardou P, Fabre S, et al. Genome-wide identification of the mutation underlying fleece variation and discriminating ancestral hairy species from modern woolly sheep. Mol Biol Evol. 2017;msx114.
