# Supplementary material for: Revealing the selection history of adaptive loci using genome-wide scans for selection: an example from domestic sheep
Source: BMC Genomics. 2018 Jan 23;19:71. doi: 10.1186/s12864-018-4447-x (PMC5778797; doi:10.1186/s12864-018-4447-x)
Supplement: Supplementary file 12 — Mutations in MC1R. Mutations found in MC1R region in Noire du Velay, Romanov and Suffolk sheep. (PDF 187 kb) [file 12864_2018_4447_MOESM12_ESM.pdf]

Seq. name	Matrix Family	Detailed Family Information	Matrix	Detailed Matrix Information	Tissue	Opt.	Start position	End position	Anchor position	Strand	Core sim.	Matrix sim.	Mat. sim. - opt.	Evidence #	Sequence
MC1R_ROM	O$MTEN	Core promoter motif ten elements	O$DMTE.01	Drosophila motif ten element		0.77	1096	1116	1106	+	0.875	0.785	0.015	0	gtgaccaATCGcagctcggcg
MC1R_SUF_A-G-del	V$HASF	HIF-1 ancillary sequence family	V$HAS.01	HIF-1 ancillary sequence		0.91	1212	1222	1217	+	1	0.91	0	0	tctCACGccct
MC1R_SUF_A-G-ins	V$CP2F	CP2-erythrocyte Factor related to drosophila Elf1	V$TCFCP2L1.01	Transcription factor CP2-like 1 (LBP-9)	Blood Cells	0.87	1372	1390	1381	+	1	0.877	0.007	0	ggCTGGttcagggctgagg
MC1R_SUF_A-G-ins	V$RXRF	RXR heterodimer binding sites	V$VDR_RXR.03	Bipartite binding site of VDR/RXR heterodimers, DR1 sites	Bone and Bones, Brain, Central Nervous System, Connective Tissue, Digestive System, Ear, Endocrine System, Integumentary System, Liver, Nervous System, Parathyroid Glands, Pituitary Gland, Skeleton, and Thyroid Gland	0.74	1369	1393	1381	+	1	0.746	0.006	0	catggctGGTTcagggctgaggtcc
MC1R_SUF_A-G-ins	V$PAX5	PAX-2/5/8 binding sites	V$PAX5.01	B-cell-specific activator protein	Antibody-Producing Cells, Endocrine System, Immune System, and Thyroid Gland	0.79	1372	1400	1386	+	0.81	0.793	0.003	0	ggctggTTCAgggctgaggtcccagcaca
